# Exploring 1,3,4-oxadiazolyl sulfide derivatives as antidiabetic candidates: synthesis, antioxidant activity, SAR-study, molecular docking, and DFT-insights

**DOI:** 10.1186/s13065-025-01678-w

**Published:** 2025-11-25

**Authors:** Norhan A. Khalaf, Gehad E. Said, Ehab Abdel-Latif, Heba M. Metwally

**Affiliations:** Department of Chemistry, Faculty of Science, Mansoura University, Mansoura, 35516 Egypt

**Keywords:** 1,3,4-Oxadiazole, Antioxidant, Molecular docking, SAR-study, DFT.

## Abstract

1,3,4-Oxadiazoles are a vital class of heterocyclic compounds known for their diverse biological activities. In this study, a series of eight novel 1,3,4-oxadiazolyl sulfide derivatives **4a**–**h** were synthesized and characterized using IR, NMR, and elemental analysis. The antioxidant activity of these derivatives was evaluated via DPPH and ABTS assays, revealing promising radical scavenging capabilities Compound **4 h** emerged as the most potent antioxidant with SC_50_ values of 9.88 µM (ABTS) and 12.34 µM (DPPH), outperforming standard antioxidants surpassing standard antioxidants such as ascorbic acid (SC₅₀ = 23.92 µM) and gallic acid (SC₅₀ = 21.24 µM). The impact of electron-donating and electron-withdrawing substituents on activity was demonstrated through a comprehensive structure-activity relationship (SAR) study. Molecular docking against *α*-glucosidase (PDB: 3W37) validated the potential of these compounds as enzyme inhibitors, with docking scores ranging from − 8.59 to -9.81 kcal/mol and similar modes of binding. Insights into electronic properties were obtained through density functional theory (DFT) calculations, emphasizing that compound with the lowest HOMO–LUMO energy gaps (**4b**) exhibited higher polarizability and enhanced reactivity, which correlates with their biological antioxidant performance. This integrated study underscores the therapeutic potential of these derivatives as antioxidants and enzyme inhibitors, offering paths for further drug development.

**Supplementary Information:**

The online version contains supplementary material available at 10.1186/s13065-025-01678-w.

## Introduction

Normal aerobic cellular metabolism frequently produces free radicals. An excess of free radicals can harm biomolecules (lipids, proteins, and DNA) through oxidative stress, which can ultimately result in tissue damage and cellular death. Free radicals contribute to the onset of chronic inflammation, atherosclerosis, cancer, diabetes, cardiovascular illnesses, rheumatoid arthritis, stroke, and various other degenerative conditions in humans [1–5]. The body’s antioxidant system is essential for preventing any damage brought on by free radicals. Also, antioxidants are able to scavenge free radical species [6]. In order to combat environmental and clinical factors that have accumulated in the body, such as oxidative stress, radiation, pressure, smoking, and contaminated particles, a number of recent studies have looked into the administration of strong antioxidants [7, 8].

Therefore, it is recommended that we enhance the antioxidant content of our bodies through a natural, balanced diet. Correspondingly, medicinal chemistry attempts to create antioxidant agents that are practical and effective for clinical use [6, 7, 9]. Type 2 diabetes mellitus is one of the most prevalent chronic metabolic disorders, characterized by persistent hyperglycemia resulting from impaired insulin action or secretion [10–12]. A key factor in postprandial hyperglycemia is the enzyme α-glucosidase, which catalyzes the hydrolysis of dietary oligosaccharides and disaccharides into glucose [13]. Inhibition of α-glucosidase slows carbohydrate digestion and glucose absorption, thereby reducing postprandial blood glucose spikes. Clinically approved α-glucosidase inhibitors such as acarbose, miglitol, and voglibose demonstrate the therapeutic relevance of this approach in type 2 diabetes management [14]. Developing novel α-glucosidase inhibitors with concurrent antioxidant activity may therefore provide dual benefits—improving glycemic control while reducing oxidative stress, a key contributor to diabetic complications.

**Fig. 1:**
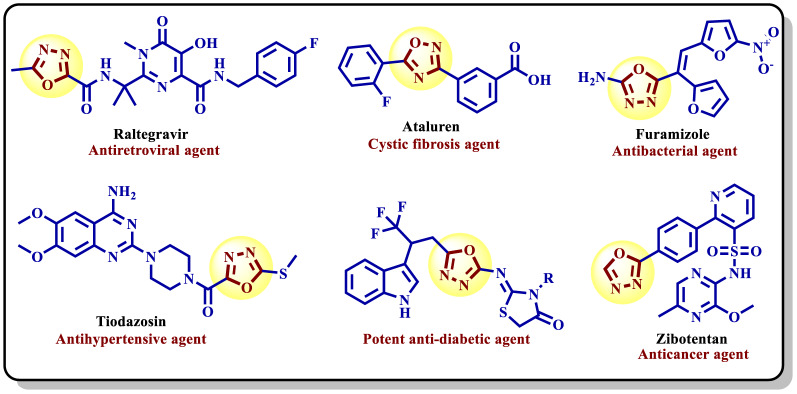
Commercial drugs with the privilege oxadiazole nucleus

Heterocyclic pharmacophores such as 1,2,4-oxadiazole-substituted scaffolds were used to develop strong antiproliferative drugs [6, 15–17]. They have therefore lately been developed as potential medications to combat these various illnesses, including aging in aerobic organisms, inflammation, atherogenesis, and carcinogenesis [18, 19]. Because of their significant biological characteristics, 1,3,4-oxadiazoles are a special type of heterocyclic system that has been observed in recent decades. These nitrogen-containing heterocycles with five members are essential, particularly in medicinal chemistry [20–23]. 1,3,4-Oxadiazole and its derivatives have demonstrated a wide array of biological activities, including antitubercular [24, 25], anti-inflammatory [26], analgesic [27], anticancer [28], antimalarial [29], anti-allergic agent [30], antibacterial [31, 32], anticonvulsant [33], and vasodilator activities [34] (Fig. 1). The oxadiazole nucleus is a fundamental component of many marketed drugs, such as the anti-cancer drug zibotentan, the HIV integrase inhibitor raltegravir, and the hypertension drug nesapidil [35–37] (Fig. 1**)**. Moreover, some 1,3,4-oxadiazole derivatives showed promising *α*-glucosidase inhibition [22, 32].

**Fig. 2:**
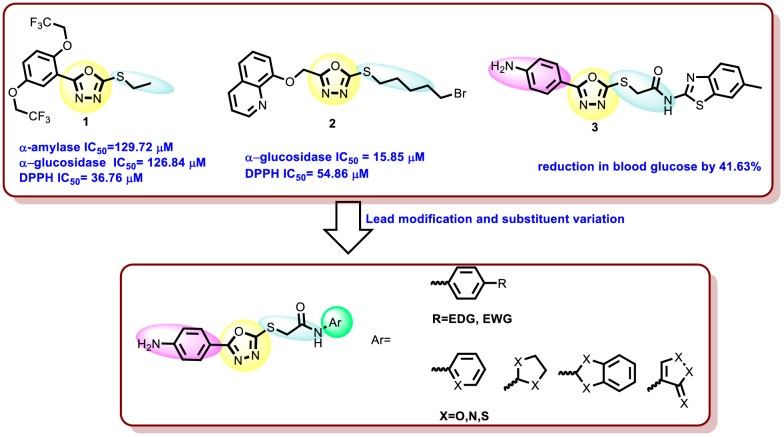
Potent antidiabetic agents with α-glucosidase inhibition, antioxidant properties and design methodology of the novel compounds

The 1,3,4-oxadiazolyl sulphide derivative **1** exhibited a potent inhibitory effect against *α*-glucosidase and α-amylase (IC_50_ = 126.84 and 129.72 µM in comparison to acarbose IC_50_ = 53.82 µM), in addition to potent antioxidant scavenging activity (DPPH IC_50_ = 36.76 µM compared to reference ascorbic acid IC_50_ = 50.30 µM) [38] (Fig. 2). Also, The 1,3,4-oxadiazolyl sulphide derivative **2** showed *α*-glucosidase(IC_50_ = 15.85 µM in comparison to acarbose IC_50_ = 17.85 µM), in addition to a relevant antioxidant activity with an IC_50_ of 54.86 µM in comparison to gallic acid (IC_50_ = 26.23 µM) [39]. Interestingly, the The 1,3,4-oxadiazolyl sulphide derivative **3** significantly decreased the blood glucose level in diabetic rat [40].

Scientists have created a number of novel techniques in recent years for the synthesis of 1,3,4-oxadiazole derivatives. Using an acyl hydrazine derivative, Harish et al. have reported synthesizing 2-monosubstituted-1,3,4-oxadiazole from triethylorthoformate [41]. Modern synthetic methods for 1,3,4-oxadiazole, rely on the oxidation of acylhydrazones and the cyclization of hydrazides or acylthiosemicarbazides [42–46] using a range of reagents such as sulfuric acid, phosphorus oxychloride, or thionyl chloride, typically under harsh reaction conditions.

Stimulatingly, the literature review highlighted 1,3,4-oxadiazole sulphide derivatives exhibited significant antidiabetic and antioxidant activity. As such, his cores serve as promising framework for developing novel antioxidants. Building on our continued interest in this field, we designed and synthesized a range of derivatives featuring these core structures (Fig. 2), incorporating the mecaptoacetamide as a diverse substituent. The resulting compounds were assessed for their antioxidant activity, and the findings were further validated through molecular docking and DFT studies.

## Results and discussion

### Chemistry

**Scheme 1:**
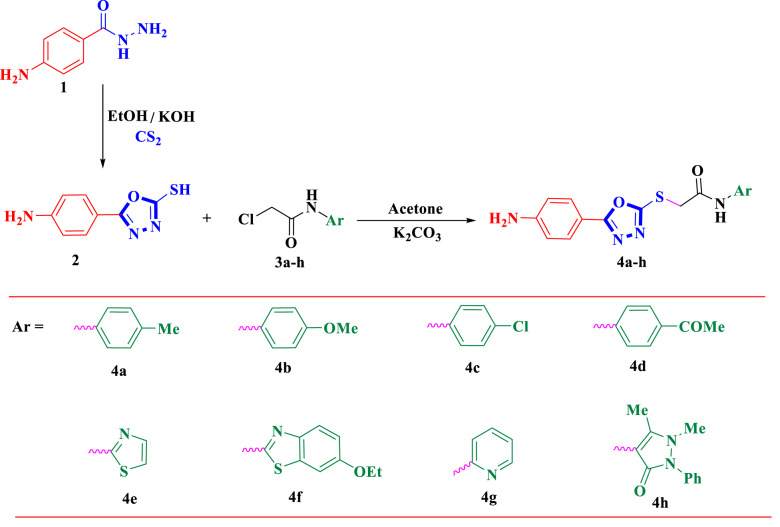
Synthesis of 1,3,4-oxadiazolyl sulfide derivatives **4a-h**

The multifunctional precursor 5-(4-aminophenyl)-1,3,4-oxadiazole-2-thiol (**2**) has been prepared according to the reported conditions [47] through a cyclization reaction of 4-aminobenzohydrazide (**1**) with carbon disulfide. Compound (**2**) has a great affinity to undergo alkylation reaction with numerous aromatic and heterocyclic 2-chloroacetamide derivatives (Scheme 1). Therefore, a series of linked sulfur scaffolds bearing a 1,3,4-oxadiazole moiety (**4a-h**) was constructed. The reaction started through nucleophilic attack of compound (**2**) on various aromatic and heterocyclic chloroacetamide derivatives (**3a-h**), namely, *p*-toluidine, *p*-anisidine, *p*-chloroaniline, *p*-aminoacetophenone, chloroacetamide thiazole, chloroacetamide ethoxybenzothiazole, chloroacetamide pyridine, and chloroacetamide antipyrine. The reaction proceeded by stirring a mixture of compound **2** and chloroacetamide derivatives (**3a-h**) in dry acetone and K_2_CO_3_ to afford the corresponding 1,3,4-oxadiazolyl sulfide scaffolds **4a–h**, respectively (Scheme 1). The chemical structure of the newly synthesized 1,3,4-oxadiazolyl sulfide derivatives **4a–h** was confirmed based on their elemental and spectral data. The distinctive absorption bands at 3405, 3319, and 3265 cm^− 1^ that correspond to the stretching vibrations of amino and imino groups and at 1702 and 1674 cm^− 1^ that correspond to two carbonyl groups (acetyl and amidic) were found in compound **4d**’s infrared spectrum. Singlet signals at δ 2.52 and 4.29 ppm in the ^1^H-NMR spectrum were attributed to the protons of the methyl (CH_3_) and methylene (CH_2_) groups. The ^13^C-NMR spectrum showed distinct signals for the amidic and acetyl carbonyl groups at δ 160.90 and 165.75 ppm, respectively. The distinctive absorption bands at 3470, 3380, 3084, and 1671 cm^− 1^ in the infrared spectrum of **4f** were found to correspond to the stretching vibrations of amino, imino, and amidic carbonyl groups, respectively. Methyl protons showed a triplet signal at δ 1.33 ppm in the ^1^H-NMR spectrum, while methylene protons showed quartet and singlet signals at δ 4.03 and 4.37 ppm. At δ 5.94 ppm, the amino group’s protons showed up as a singlet signal. Characteristic methyl and two methylene carbon signals were detected in the ^13^C-NMR spectrum at δ = 14.70, 35.56, and 63.61 ppm, respectively. The amidic carbonyl group carbon was represented by the typical signal at δ 166.16 ppm.

### In vitro antioxidant (DPPH and ABTS) radical scavenging activities

The synthetic 1,3,4-oxadiazolyl sulfide derivatives **4a-h** were calculated for their in vitro antioxidant activity using DPPH and ABTS assays according to the previously described procedure [48]. The antioxidant capacity for the compounds was expressed as SC_50_ (µM) for both DPPH and ABTS assays. The radical scavenging activity results were calculated from the absorbance values of the oxidized ABTS.^+^ or DPPH.^+^, detected by color and used to calculate radical scavenging concentration SC_50_ (Table 1, **Fig. S19**,** S20**). The obtained results were compared to standard ascorbic acid with SC_50_ = 23.92 µM (for DPPH) and gallic acid with SC_50_ = 21.24 µM (for ABTS), respectively. The compounds showed DPPH & ABTS scavenging activities in the range of SC_50_ = 12.34 to 87.89 µM (for DPPH) and 9.88 to 74.42 µM (for ABTS), respectively. According to the ABTS assay, the best synthetic compound was **4 h**, since it showed the lowest SC_50_ value of 9.88 µM. The scavenging activities for the rest of the compounds were arranged as **4 h** < **4b** < **4f** < **4a** < **4c** < **4e** < **4d** < **4 g**, with SC_50_ values 9.88, 12.84, 14.02, 17.48, 39.64, 55.95, 68.06 and 74.42 µM. While for the DPPH assay, **4 h**, **4a**, **4b** and **4c** showed superior scavenging activity than ascorbic acid. The compounds are arranged as **4 h** < **4a** < **4b** < **4c** < **4d** < **4f** < **4e** < **4 g** with SC_50_ values of 12.34, 12.63, 13.19, 22.38, 25.56, 26.20, 37.89, and 87.89 µM. Although the present study demonstrates the antioxidant and α-glucosidase inhibitory potential of oxadiazolyl sulfide derivatives through in vitro and in silico approaches, further validation is required.

**Table 1:** ABTS & DPPH Inhibition of 1,3,4-oxadiazole derivatives **4a**-**h**

Compounds	ABTS (SC_50_ value, µM)	DPPH data (SC_50_ value, µM)
4a	17.48	12.63
4b	12.84	13.19
4c	39.64	22.38
4d	68.06	25.56
4e	55.95	37.89
4f	14.02	26.20
4 g	74.42	87.89
4 h	9.88	12.34
Ascorbic acid		23.92
Gallic acid	21.24	

### Structure-activity relationship (SAR)

It was reported that 1,3,4-oxadiazole derivatives to have measurable DPPH/ABTS scavenging. Sulfur substituents (thioether / thioamide) frequently enhance radical reactivity hydrogen-atom transfer (HAT) and single-electron transfer (SET) pathways. The synthesized compounds generally exhibited almost equal activity against DPPH radicals compared to ABTS radicals.

**Fig. 3:**
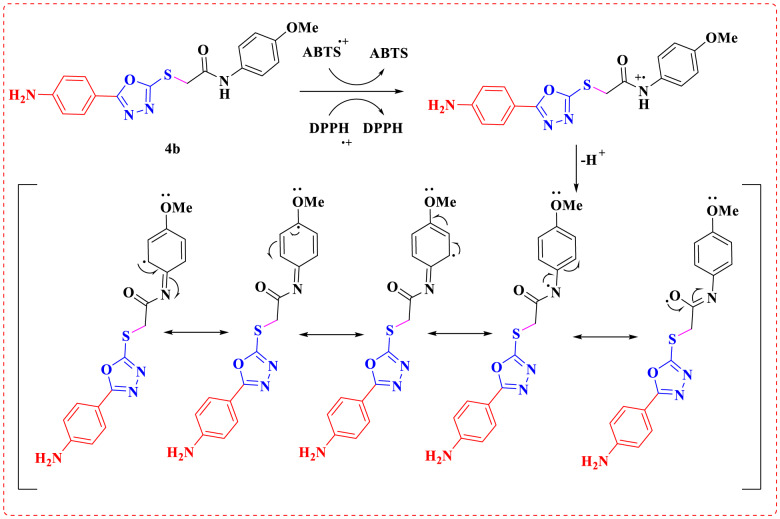
The proposed mechanism of compound **4b** for the antioxidant DPPH assay

The activity varies according to the substituents and the mechanistic drivers. Overall rank, **4 h** derivative containing pyrazolone group anchored on the oxadiazole ring through mercapto acetamide linker exhibited the strongest antioxidant activities (SC_50_ = 12.34 µM for DPPH, SC_50_ = 9.88 µM for ABTS, respectively). This may be attributed to the strong resonance stabilization of the resulting radical across the compound with the privilege of the phenyl ring hanged on pyrazolone moiety. The proposed mechanism by which the synthesized compounds function as scavengers is illustrated in Figs. 2 and 3 [49]. Additionally, the structure–activity relationship (SAR) insights outlined below can be derived from the data presented in Table 1:

1. In *p*-substituted phenyl series, compound **4a**,**4b** containing methyl and methoxy groups, improved the scavenging activity among the tested compounds (SC_50_ = 17.48 and 12.84 µM for DPPH and SC_50_ = 12.63 and 13.19 µM for ABTS, respectively). This can be attributed to the strong electron-donating properties of methyl and methoxy groups which stabilize the formed radical through resonance and inductive effects, which reduce the N-H bond energy and subsequently enhance radical scavenging activity. On the other hand, the antioxidant activity was diminished by the presence of Cl- or COCH_3_ group, as demonstrated by the SC_50_ values of compound **4d**,** 4c** (22.38 and 25.56 µM for DPPH and 39.64 and 68.06 µM for ABTS). The e donation was hindered by the effect of electron withdrawing effect of the groups, destabilizing the radical intermediate.
2. Replacing the phenyl ring with heterocyclic thiazole ring **4e** shows a slight decrease in the antioxidant activity (SC_50_ = 37.89 µM and 55.95 µM for DPPH and ABTS, respectively). The fused benzothiazole with ethoxy substituent derivative **4f** shows imminent scavenging activity compared to the thiazole ring (SC_50_ = 26.20 µM and 14.02 µM for DPPH and ABTS, respectively). Providing larger conjugation and better radical delocalization than simple thiazole.
3. Replacing the phenyl ring with pyridine ring **4 g** markedly exhibit the weakest antioxidant activity (SC_50_ = 87.89 µM and 74.42 µM for DPPH and ABTS, respectively). The pyridine electron practice the withdrawing effect lowering the radical donation ability.

**Fig. 4:**
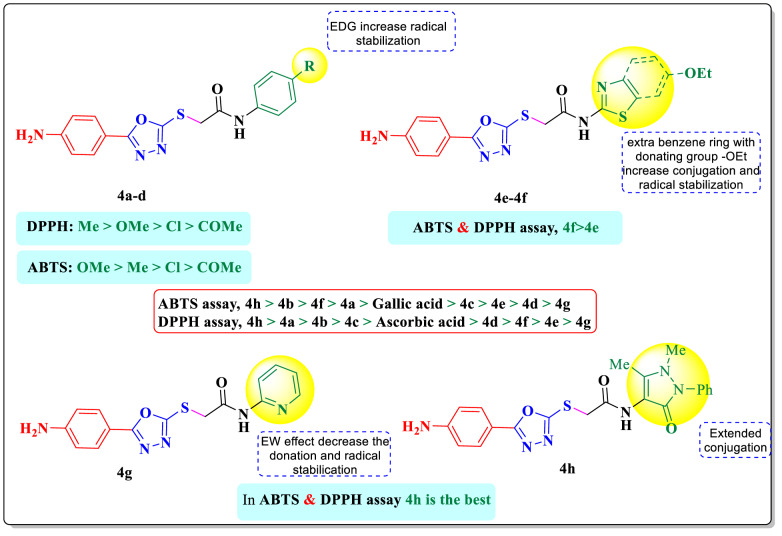
SAR of the synthesized 1,3,4-oxadiazolyl sulfide derivatives **4a-h**

### Molecular docking

Molecular docking was conducted to examine the interactions and binding behavior of the synthesized compounds with the *α*-glucosidase enzyme. This will directly contribute to the SAR and biological relevance of the findings reported. The enzyme crystallographic coordinates were retrieved from www.rcsb.org using the PDB ID of 3w37 for *α*-glucosidase [50, 51]. The docking protocol was validated, proving its reliability for the intended study. This was evident in Fig. 4, where the alignment between the native co-crystallized ligand and its re-docked pose within the active pocket showed a minimal RMSD value of 0.5233 Å. The docking analysis demonstrated that the 1,3,4-oxadiazolyl sulfide derivatives **4a-h** exhibited significant potential against the *α*-glucosidase enzyme. These compounds consistently formed three key interactions with three identical amino acids, which are likely crucial for stabilizing the ligand inside the active site. The docking scores (kcal/mol) and comprehensive binding interactions are outlined in Table 2, with binding energies ranging from − 8.59 to -9.81 kcal/mol.

**Table 2:** The molecular Docking results for the synthesized 1,3,4-oxadiazolyl sulfide derivatives targeting the protein structure with PDB ID: 3W37

Code	S (energy score) (Kcal/mol)	Moiety	Residue	Interactions	Distance (A)
**4a**	– 8.73	*N*-aniline Oxadiazole ring *N*-Oxadiazole *N*-carboxamide Ph-ring Ph-ring	Asp 357 Arg 552 Met 470 Asp 232 Ile 233 Ala 234	H-donor π –cation H-donor H-donor π-H interaction π-H interaction	3.13 4.23 3.34 3.44 4.52 4.55
**4b**	– 9.07	*N*-aniline *N*-Oxadiazole Ph-ring	Asp 357 Arg 552 Ile 233	H-donor H-acceptor π-H interaction	2.80 3.25 4.45
**4c**	– 8.77	*N*-aniline Oxadiazole ring *N*-Oxadiazole *N*-carboxamide Ph-ring Ph-ring	Asp 357 Arg 552 Met 470 Asp 232 Ile 233 Ala 234	H-donor π-cation H-donor H-donor π-H interaction π-H interaction	3.14 4.23 3.34 3.43 4.52 4.56
**4d**	– 9.00	*N*-aniline Oxadiazole ring *N*-Oxadiazole *N*-carboxamide Ph-ring Ph-ring Ph-ring	Asp 357 Arg 552 Met 470 Asp 232 Ile 233 Ile 233 Ala 234	H-donor π-cation H-donor H-donor π-H interaction π-H interaction π-H interaction	3.14 4.23 3.34 3.35 4.48 4.71 4.60
**4e**	– 8.64	*N*-aniline Oxadiazole ring *N*-Oxadiazole *S*-thiazole	Asp 357 Arg 552 Met 470 Asp 232	H-donor π-cation H-donor H-donor	3.08 4.27 3.48 3.48
**4f**	– 9.35	*N*-aniline Oxadiazole ring *N*-Oxadiazole Thiazole ring Thiazole ring Thiazole ring *N*-carboxamide *S*-thiazole	Asp 357 Arg 552 Met 470 Ile 233 Ile 233 Ala 234 Asp 232 Asp 232	H-donor π -cation H-donor π-H interaction π-H interaction π-H interaction H-donor H-donor	3.15 4.23 3.34 4.39 4.72 4.53 3.25 3.58
**4 g**	– 8.59	*N*-aniline Oxadiazole ring *N*-Oxadiazole	Asp 357 Arg 552 Met 470	H-donor π-cation H-donor	3.11 4.46 3.48
**4 h**	– 9.81	*N*-aniline Oxadiazole ring *N*-Oxadiazole *O*-pyrazolone Pyrazolone ring	Asp 357 Arg 552 Met 470 Ala 234 Ile 233	H-donor π-cation H-donor H-acceptor π-H interaction	3.19 4.28 3.40 3.57 4.33

**Fig. 5:**
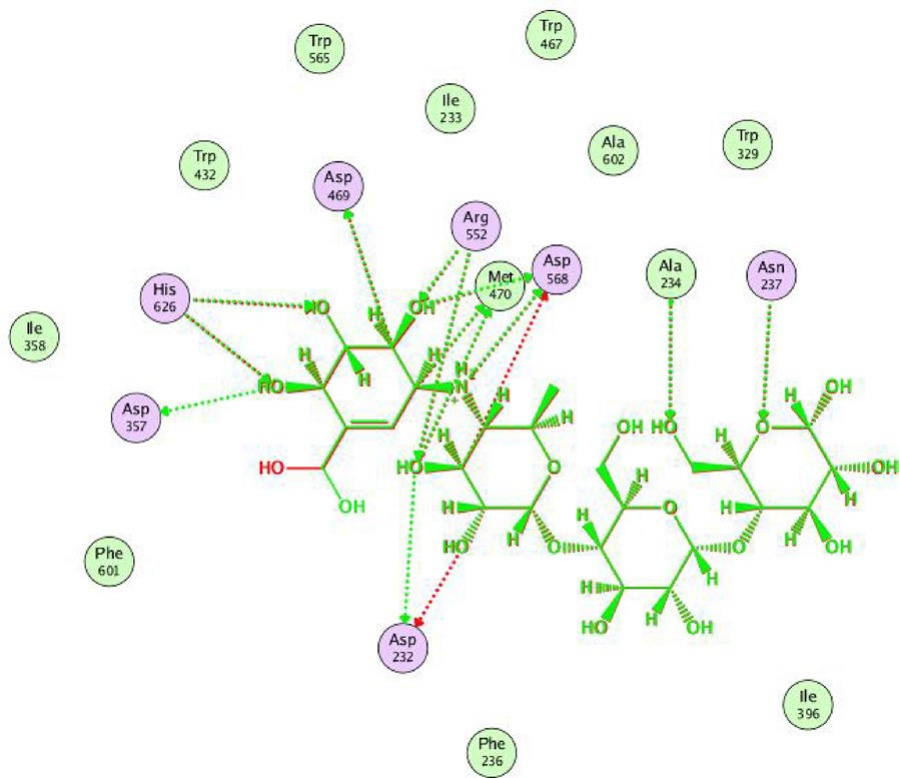
The 2D figure shows the overlay between the cocrystallized ligand (acarbose) (red) and redocked acarbose (green)

**Fig. 6:**
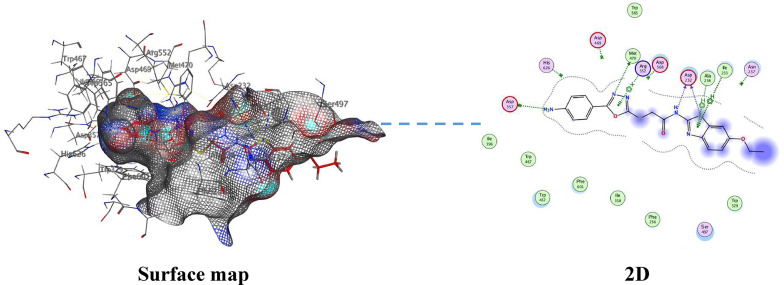
The binding interaction of the 1,3,4-oxadiazolyl sulfide derivative **4f** with (PDB ID: 3W37)

The main scaffold in all synthesized compounds oxadiazole ring, is hydrogen bonded to Arg552 as presented in Fig. 5 and **Figs. S21-S28**. The *N*-atom of the oxadiazole ring attached to Met 470 through a hydrogen bond in all derivatives except **4b**. All derivatives show the same *π*–cation interaction between the amino group and Asp 357. The NH of the carboxamide group shows a hydrogen bond interaction with Asp 232 in **4a**, **4c**, **4d**, and **4f**. The phenyl ring in derivatives **4a-d** shows a number of hydrophobic interactions with two amino acids present in the active site, Ile 233 and Ala 234. In derivative **4e**, the thiazole ring is attached to Asp 232 present in the active site through a hydrogen bond, while derivative **4f** shows enforced binding to the thiazole ring through hydrophobic interactions with Ile 233 and Ala 234. Also, in derivative 4 h, the pyrazolone ring is attached to the active site through a hydrogen bond between the O-atom of the pyrazolone ring with Ala 234 and a hydrophobic bond between the pyrazolone ring and Ile 233. The alignment of all derivatives **4a-h** inside the active pocket of α-glucosidase, showing similar binding mode of action (Fig. 6).

**Fig. 7:**
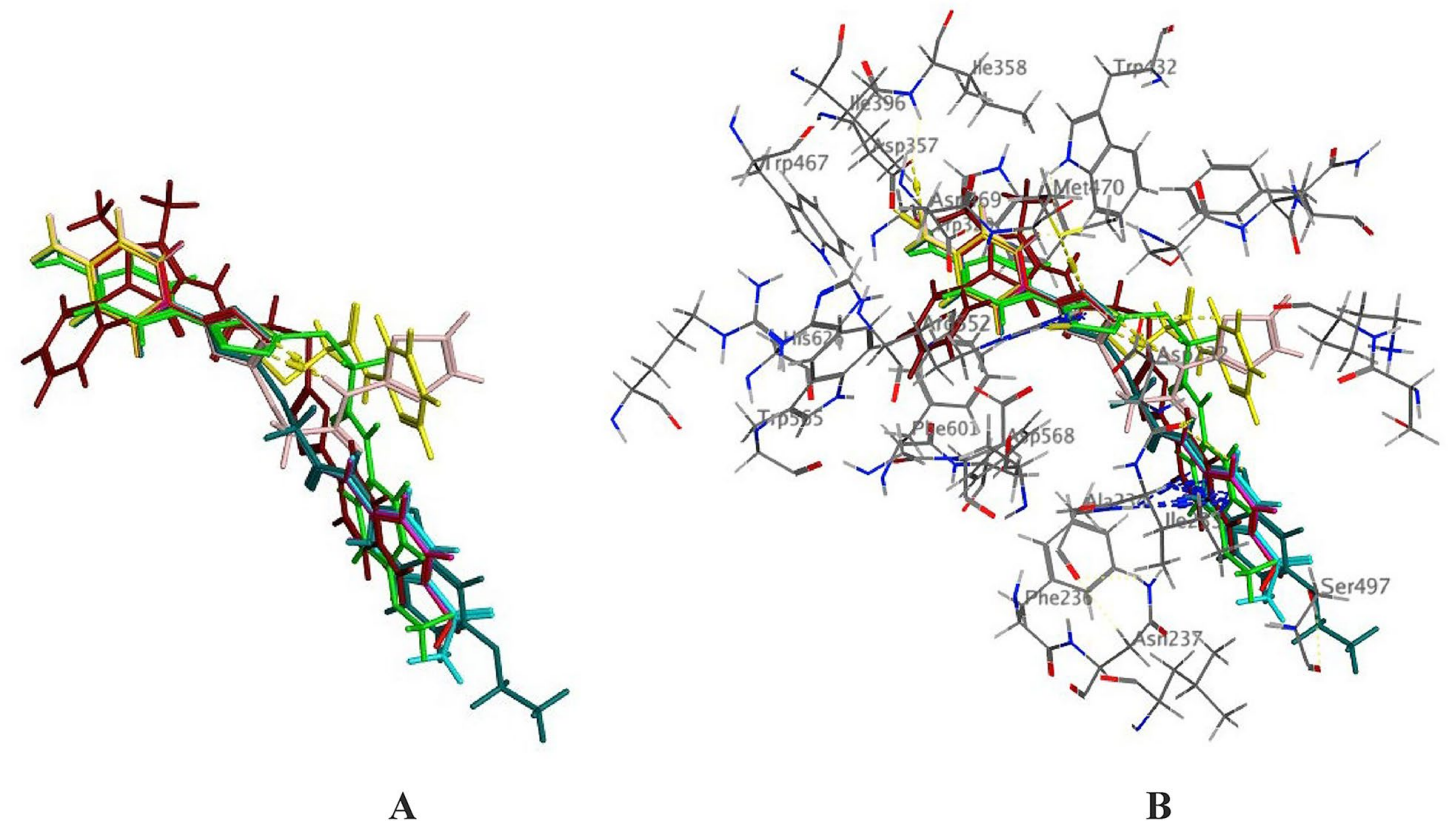
3D visualization for the alignment of docked poses of 1,3,4-oxadiazolyl sulfide derivatives **4a-h** inside the active pocket of (PDB ID: 3W37) **A** without showing a receptor. **B** showing binding with receptor

### Frontier molecular orbitals (FMOs)

The 1,3,4-oxadiazole derivatives’ **4a-h** electrical features and structural differences are determined by DFT simulations. An application of molecular orbital theory is Frontier Molecular Orbitals (FMOs), it can determine the electronic properties and the chemical reactivity of the synthesized derivatives [52, 53]. Through examining Fig. 7, **Fig. S29-S36** and Table 3, optimized structures, HOMO and LUMO (highest occupied and lowest unoccupied molecular orbitals) distributions of the investigated molecules can be deduced.

**Table 3:**
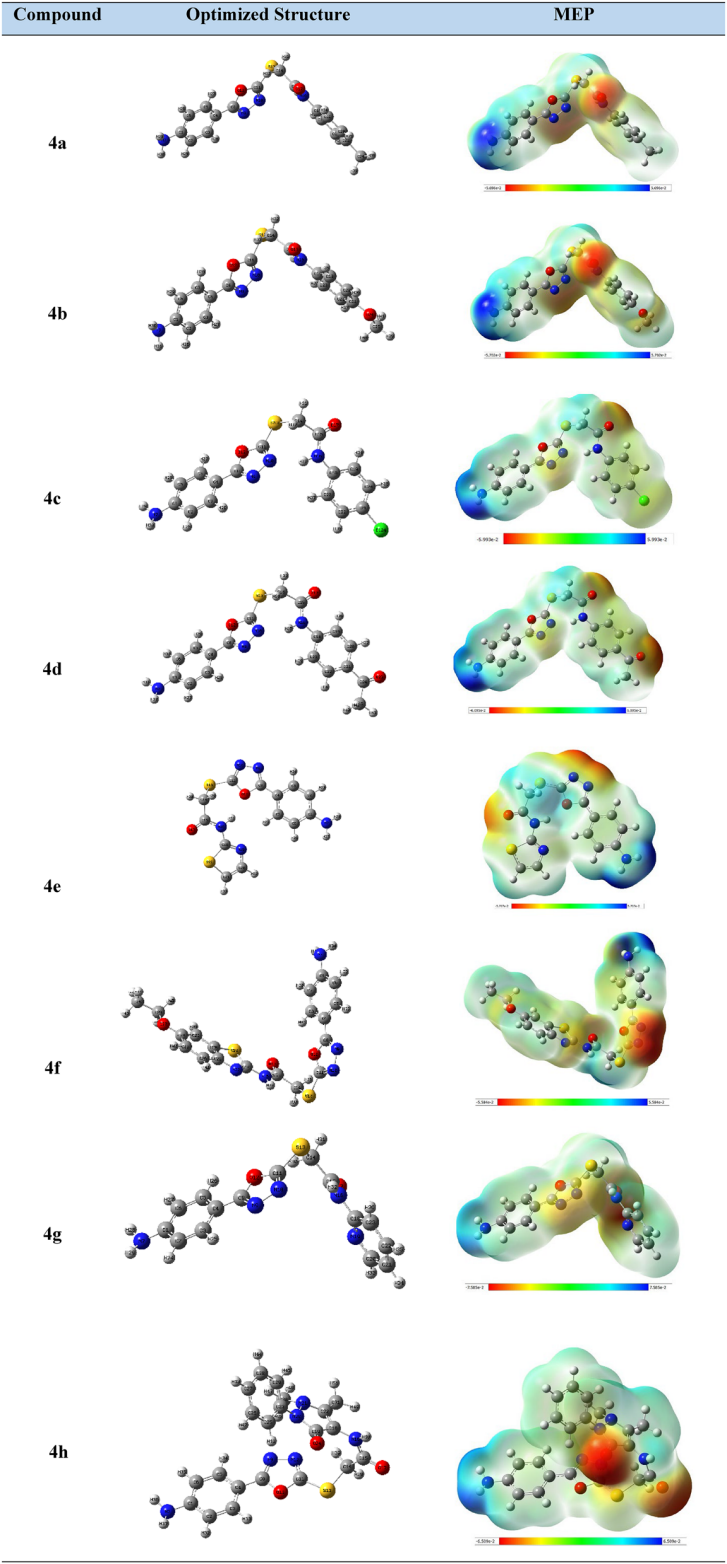
Optimized structures, electron density of the 1,3,4-oxadiazolyl sulfide derivatives 4a-h

**Fig. 8:**
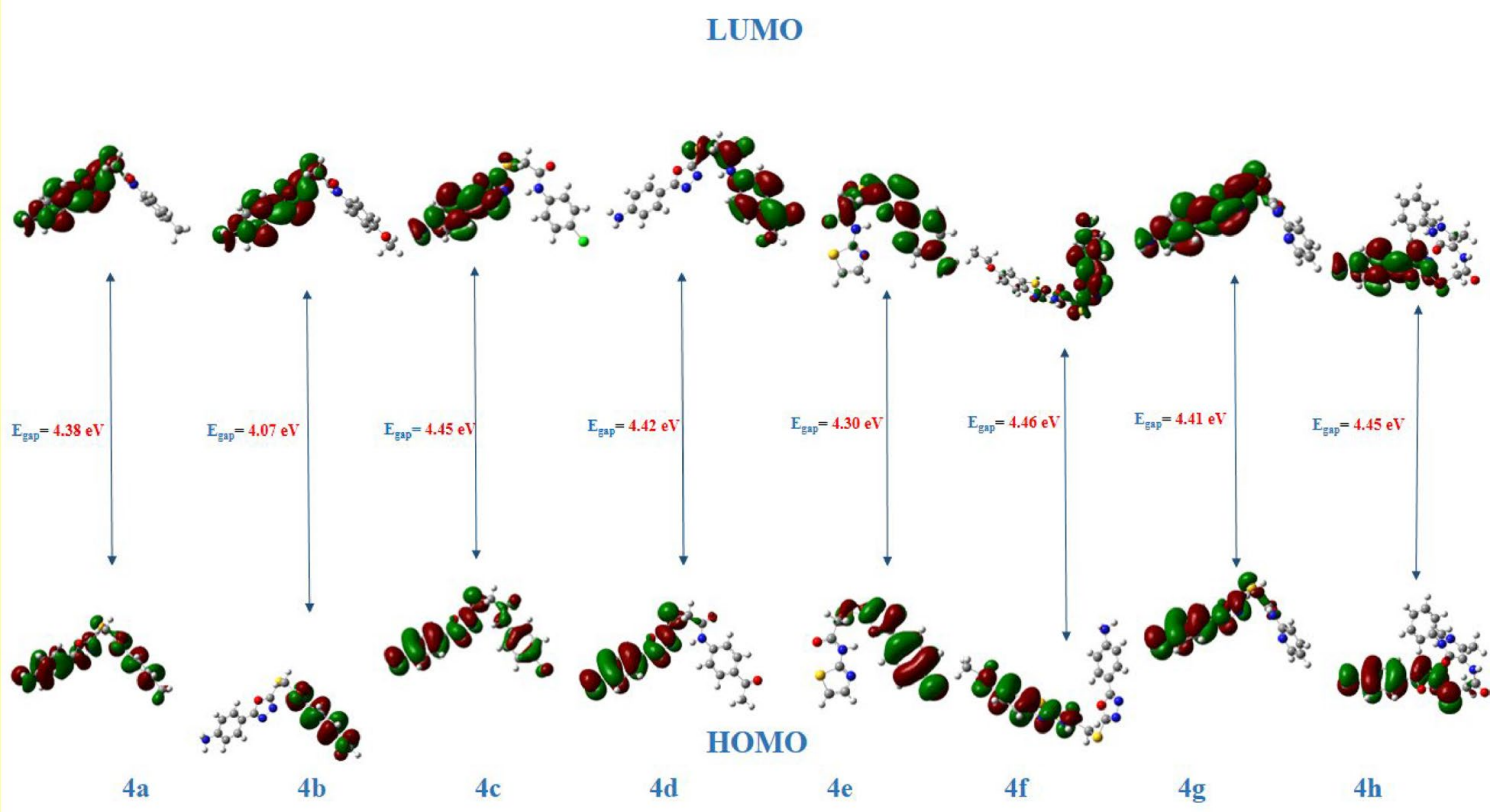
FMO energies of 1,3,4-oxadiazolyl sulfide derivatives **4a-h**

As presented in Table 4, the findings were reflected in the energy values (E_HOMO_ and E_LUMO_). The E_HOMO_ values were relatively similar, ranging from 6.21 to 5.78 eV, while the E_LUMO_ values fell within the range of 1.84 to 1.52 eV. Furthermore, the 4-acetylphenyl derivative demonstrated the highest E_HOMO_, where **4d** >**4c** >**4a** >**4b**. The values of the HOMO–LUMO energy gap (ΔE_H−L_) ranged between 4.46 − 4.07 eV. They are arranged in the order: **4f** < **4 h** < **4c** < **4d** < **4 g** < **4a** < **4e** < **4b**. The HOMO–LUMO energy gap correlate the relative low ΔE value of derivate **4b** with its scavenging activity in both ABTS and DPPH with immenant SC_50_ value 12.84 and 13.19 µM. A small band gap means the compound is more polarizable, linked to low kinetic stability and high chemical reactivity, and the compound is termed soft [54]. **4b** derivative is the softest compound in the series (Fig. 8).

**Table 4:** The HOMO energy (E_HOMO_), LUMO energy (E_LUMO_), HOMO-LUMO energy gap (E_gap_) in eV, electronegativity (χ), global hardness (η), softness (δ), and electrophilicity (ω)

Molecules	E_HOMO_ eV	E_LUMO_ eV	E_gap_	χ	η	δ	ω
4a	– 6.09	– 1.71	4.38	3.90	2.19	0.46	3.46
4b	– 5.78	– 1.71	4.07	3.74	2.03	0.49	3.45
4c	– 6.16	– 1.71	4.45	3.93	2.23	0.45	3.47
4d	– 6.21	– 1.78	4.42	3.99	2.21	0.45	3.61
4e	– 6.14	– 1.84	4.30	3.99	2.15	0.47	3.71
4f	– 5.90	– 1.44	4.46	3.67	2.23	0.45	3.01
4 g	– 6.08	– 1.68	4.41	3.88	2.20	0.45	3.42
4 h	– 5.98	– 1.52	4.45	3.75	2.23	0.45	3.16

## Experimental

### Chemistry

The supplementary data contains information on all devices utilized in the chemistry section’s analytical experiments. Compounds **2** [47] and **3a**-**h** [55–62] were prepared as reported.

### Synthesis of 2-((5-(4-aminophenyl)-1,3,4-oxadiazol-2-yl)thio)-N- acetamide derivatives 4a-h:

A suspension of chloroacetamide-based compounds **3a-h** (10 mmol), 2-mercapto-1,3,4-oxadiazole compound **2** (1.93 g, 10 mmol), and potassium carbonate (1.38 g, 10 mmol) was stirred overnight in 30 mL dry acetone. The resulting solid was obtained by pouring the mixture into ice water. After collection, the solid underwent recrystallization using absolute ethanol, yielding the corresponding pure oxadiazole sulfide compounds **4a-h**.

### 2-((5-(4-Aminophenyl)-1,3,4-oxadiazol-2-yl)thio)-N-(p-tolyl)acetamide (4a)

Yellow crystals; yield 44%; m.p. = 230–231 °C. TLC solvent [pet. ether: ethyl acetate (1:3)]. IR (ν/cm^− 1^): 3356, 3300, 3190 (NH_2_, NH), 1665 (C = O). ^1^H NMR (500 MHz) (DMSO): δ 2.23 (s, 3 H, CH_3_), 4.29 (s, 2 H, S-CH_2_), 7.11 (d, *J* = 8.50 Hz, 2 H), 7.44 (t, *J* = 8.50 Hz, 3 H), 7.70–7.78 (m, 3 H, Ar-H, NH_2_), 7.84 (d, *J* = 8.50 Hz, 1H, ), 7.90 (d, *J* = 9.00 Hz, 1H), 10.39 ppm (s, 1H, N-H). ^13^C NMR (125 MHz): δ 20.71, 40.40, 56.20, 113.72, 119.35 (2 C), 120.32 (2 C), 120.42, 126.86, 127.05, 127.78 (2 C), 128.03, 129.37, 129.39, 129.44 ppm. *Anal*. Calcd for C_17_H_16_N_4_O_2_S (340.10): C, 59.98; H, 4.74; N, 16.46%. Found: C, 59.86; H, 4.76; N, 16.58%. HPLC: rt 14.41 min (purity 97,24%).

### 2-((5-(4-Aminophenyl)-1,3,4-oxadiazol-2-yl)thio)-N-(4 methoxyphenyl)acetamide (4b)

White crystals; yield 35%; m.p. = 240–241 °C. TLC solvent [pet. ether: ethyl acetate (1:2)]. IR (ν/cm^− 1^): 3368, 3062 (NH_2_, NH), 1634 (C = O). ^1^H NMR (500 MHz) (DMSO): δ 3.71 (s, 3 H, O-CH_3_), 4.21 (s, 2 H, S-CH_2_), 5.96 (s, 2 H, NH_2_), 6.62 (d, *J* = 8.50 Hz, 2 H), 6.80–6.93 (m, 2 H), 7.44–7.52 (m, 2 H), 7.56 (d, *J* = 6.50 Hz, 2 H), 10.26 ppm (s, 1H, N-H). ^13^C NMR (125 MHz): δ 36.67, 55.30, 113.68 (4 C), 114.09 (2 C), 120.89 (2 C), 128.05 (3 C), 155.74 (2 C), 165.01 ppm (2 C). *Anal*. Calcd for C_17_H_16_N_4_O_3_S (356.09): C, 57.29; H, 4.53; N, 15.72%. Found: C, 57.48; H, 4.55; N, 15.84%. HPLC: rt 13,38 min (purity 93,62%).

### 2-((5-(4-Aminophenyl)-1,3,4-oxadiazol-2-yl)thio)-N-(4-chlorophenyl)acetamide (4c)

Golden yellow crystals; yield 60%; m.p. = 228–229 °C. TLC solvent [pet. ether: ethyl acetate (1:2)]. IR (ν/cm^− 1^): 3343, 3308 (NH_2_, NH), 1650 (C = O). ^1^H NMR (500 MHz) (DMSO): δ 4.32 (s, 2 H, S-CH_2_), 7.31–7.43 (m, 4 H), 7.50–7.62 (m, 4 H), 7.76 (s, 2 H), 10.56 ppm (s, 1H, NH). ^13^C NMR (125 MHz): δ 36.50, 114.02 (2 C), 121.23 (2 C), 121.34, 123.17, 127.77, 128.39, 129.11, 129.26 (2 C), 138.28, 166.50, 167.34, 168.18 ppm. *Anal*. Calcd for C_16_H_13_ClN_4_O_2_S (360.04): C, 53.26; H, 3.63; N, 15.53%. Found: C, 53.29; H, 3.65; N, 15.48%.%. HPLC: rt 14.75 min (purity 93,87%).

### N-(4-Acetylphenyl)-2-((5-(4-aminophenyl)-1,3,4-oxadiazol-2-yl)thio)acetamide (4d)

White crystals; yield 78%; m.p. = 234–235 °C. TLC solvent [pet. ether: ethyl acetate (1:3)]. IR (ν/cm^− 1^): 3405, 3319, 3265 (NH_2_, NH), 1702, 1674 (2 C = O). ^1^H NMR (500 MHz) (DMSO): δ 2.52 (s, 3 H, CH_3_), 4.29 (s, 2 H, S-CH_2_), 5.92 (s, 2 H, NH_2_), 6.61 (d, *J* = 8.00 Hz, 2 H), 7.56 (d, *J* = 9.00 Hz, 2 H), 7.7 (d, *J* = 9.00 Hz, 2 H), 7.93 (d, *J* = 8.50 Hz, 2 H), 10.72 (s, 1H, N-H). ^13^C NMR (125 MHz): δ 26.46, 36.83, 109.17, 113.48 (2 C), 116.07, 118.43 (2 C), 127.90 (2 C), 129.58 (2 C), 132.06, 142.92, 152.40, 160.90, 165.75, 196.52 ppm. MS, *m/z* (%) 368 (M^+^, 38.30%), 359.81 (61.03%), 346.07 (90.57%), 326.35 (60.67%), 314.64 (94.85%), 308.35 (52.42%), 283.01 (63.09%), 249.68 (50.82%), 220.58 (80.31%), 193.95 (51.34%), 184.95 (62.58%), 174.87 (72.68%), 100.99 (92.16%), 92.00 (100.00%). *Anal*. Calcd for C_18_H_16_N_4_O_3_S (368.09): C, 58.68; H, 4.38; N, 15.21%. Found: C, 58.61; H, 4.40; N, 15.26%. HPLC: rt 14,55 min (purity 99,32%).

### 2-((5-(4-Aminophenyl)-1,3,4-oxadiazol-2-yl)thio)-N-(thiazol-2-yl)acetamide (4e)

White crystals; yield 78%; m.p. = 200–201 °C. TLC solvent [pet. ether: ethyl acetate (1:2)]. IR (ν/cm^− 1^): 3463, 3357, 3225 (NH_2_, NH), 1690 (C = O). ^1^H NMR (500 MHz) (DMSO): δ 4.32 (s, 2 H, S-CH_2_), 6.61 (d, *J* = 8.50 Hz, 2 H), 7.24 (d, *J* = 3.00 Hz, 1H), 7.48 (d, *J* = 3.00 Hz, 1H), 7.55 (d, *J* = 9.00 Hz, 2 H), 12.51 ppm (s, 1H, N-H). ^13^C NMR (125 MHz): δ 35.46, 109.57, 113.94 (2 C), 114.19, 128.16 (2 C), 137.99, 152.32, 157.92, 160.94, 165.84, 166.36 ppm. *Anal*. Calcd for C_13_H_11_N_5_O_2_S_2_ (333.38): C, 46.84; H, 3.33; N, 21.01%. Found: C, 46.89; H, 3.35; N, 21.11%. HPLC: rt 13,06 min (purity 100%).

### 2-((5-(4-Aminophenyl)-1,3,4-oxadiazol-2-yl)thio)-N-(6-ethoxybenzo[d]thiazol-2-yl)acetamide (4f)

Gray crystals; yield 60%; m.p. = 240–241 °C. TLC solvent [pet. ether: ethyl acetate (1:1)]. IR (ν/cm^− 1^): 3470, 3380, 3084 (NH_2_, NH), 1671 (C = O). ^1^H NMR (500 MHz) (DMSO): δ 1.33 (t, *J* = 7.00 Hz, 3 H, -OCH_2_*CH*_*3*_), 4.03 (q, *J* = 7.00 Hz, 2 H, -O*CH*_*2*_CH_3_), 4.37 (s, 2 H, S-CH_2_), 5.94 (s, 2 H, NH_2_), 6.60 (d, *J* = 8.00 Hz, 2 H), 7.00–7.03 (m, 1H), 7.54 (d, *J* = 2.50 Hz, 1H), 7.66 (d, *J* = 9.00 Hz, 2 H), 7.64 (d, *J* = 9.00 Hz, 1H), 12.62 ppm (s, 1H, N-H). ^13^C NMR (125 MHz): δ 14.70, 35.56, 63.61, 105.36, 113.56 (2 C), 115.44 (3 C), 121.32, 127.95 (2 C), 132.77, 142.49, 152.35, 155.51, 160.63, 166.16, 166.33 ppm. *Anal*. Calcd for C_19_H_17_N_5_O_3_S_2_ (427.08): C, 53.38; H, 4.01; N, 16.38%. Found: C, 53.57; H, 4.03; N, 16.26%. HPLC: rt 14,40 min (purity 99,86%).

### 2-((5-(4-Aminophenyl)-1,3,4-oxadiazol-2-yl)thio)-N-(pyridin-2-yl)acetamide (4 g)

Brown crystals; yield 71%; m.p. = 195–196 °C. TLC solvent [pet. ether: ethyl acetate (1:2)]. IR (ν/cm^− 1^): 3463, 3308, 3195 (NH_2_, NH), 1690 (C = O). ^1^H NMR (500 MHz) (DMSO): δ 4.37 (s, 2 H, S-CH_2_), 5.93 (s, 2 H, NH_2_), 6.61 (d, *J* = 8.50 Hz, 2 H), 7.12 (t, *J* = 6.50 Hz, 1H), 7.56 (d, *J* = 9.00 Hz, 1H), 7.76–7.79 (m, 2 H, Ar-H), 8.02 (s, 1H, Ar-H), 8.32 (d, *J* = 4.50 Hz, 1H, Ar-H), 10.88 ppm (s, 1H, NH). ^13^C NMR (125 MHz): δ 36.6, 109.36, 113.65, 113.68, 120.03, 128.10 (2 C), 138.56, 148.26 (2 C), 151.69, 152.52, 161.07, 166.22, 166.33 ppm. *Anal*. Calcd for C_15_H_13_N_5_O_2_S (327.08): C, 55.04; H, 4.00; N, 21.39%. Found: C, 55.12; H, 4.05; N, 21.45%. HPLC: rt 16,40 min (purity 89,94%).

### 2-((5-(4-Aminophenyl)-1,3,4-oxadiazol-2-yl)thio)-N-(1,5-dimethyl-3-oxo-2-phenyl-2,3-dihydro-1 H-pyrazol-4-yl)acetamide (4 h)

White crystals; yield 30%; m.p. = 225–226 °C. TLC solvent [pet. ether: ethyl acetate (1:1)]. IR (ν/cm^− 1^): 3412, 3295, 3181 (NH_2_, NH), 1693 (2 C = O). ^1^H NMR (500 MHz) (DMSO): δ 2.08 (s, 3 H, CH_3_), 3.03 (s, 3 H, CH_3_), 4.27 (s, 2 H, S-CH_2_), 7.25–7.38 (m, 3 H), 7.41–7.55 (m, 2 H), 7.77 (d, *J* = 9.00 Hz, 1H), 7.95 (d, *J* = 9.00 Hz, 2 H), 9.58 (s, 2 H, NH_2_), 10.43 (s, 1H, N-H). ^13^C NMR (100 MHz): δ 14.28, 24.58, 39.28, 113.58, 114.93 (2 C), 115.00, 115.10, 118.63, 127.48, 127.83, 129.03, 129.14 (2 C), 132.16, 132.33, 147.91, 161.22, 162.98, 164.70, 169.44 ppm. *Anal*. Calcd for for C_21_H_20_N_6_O_3_S (436.13): C, 57.79; H, 4.62; N, 19.25%. Found: C, 57.98; H, 4.64; N, 19.13%. HPLC: rt 15,60 min (purity 95,43%).

### In vitro antioxidant (DPPH and ABTS) radical scavenging activities

The radical scavenging activity of the targeting oxadiazole sulfide compounds was tested using ABTS or DPPH, referring to the reported methods [48, 63]. The details of the experimental methods are provided in the Supporting Information.

### Molecular docking

Docking experiments were performed *via* Molecular Operating Environment (MOE) docking program version 2019. Crystal structures of *α*-glucosidase, PDB: 3W37, were selected for docking.

### DFT calculations

The compounds were subjected to DFT simulations to verify the intended geometries of the molecules under inquiry using Gaussian 09 W suite program [64]. The DFT calculations were carried out utilizing the Becke3–Lee–Yang– Parr (B3LYP) exchange–correlation functional [65–67] with standard 6–311 + + G(dp) basis set. The HOMO–LUMO plots and mep data were obtained using the GaussView program [68].

## Conclusion

The synthesized 1,3,4-oxadiazolyl sulfide derivatives **4a**-**h** demonstrated notable antioxidant activities, with compound **4f** exhibiting superior radical scavenging capabilities in both DPPH and ABTS assays. The structure-activity relationship analysis highlighted the role of electron-donating and withdrawing groups in modulating activity. Molecular docking studies revealed significant binding interactions with *α*-glucosidase, supporting their potential as enzyme inhibitors. Furthermore, DFT calculations provided a deeper understanding of the electronic properties of the derivatives, correlating their HOMO-LUMO gaps with biological activity. These findings collectively establish the synthesized derivatives as promising candidates as antioxidants and enzyme inhibitors against diabetes. Future investigations in cellular and in vivo models will be necessary to evaluate the anti-interference ability of these derivatives in the presence of diverse bioactive substances within complex biological systems.

## Supplementary Information

Below is the link to the electronic supplementary material.

Supplementary Material 1.

## Data Availability

The datasets used and/or analysed during the current study are available from the corresponding author on reasonable request.
