# Supplementary material for: Exploring 1,3,4-oxadiazolyl sulfide derivatives as antidiabetic candidates: synthesis, antioxidant activity, SAR-study, molecular docking, and DFT-insights
Source: BMC Chem. 2025 Nov 25;19(1):316. doi: 10.1186/s13065-025-01678-w (PMC12670869; doi:10.1186/s13065-025-01678-w)
Supplement: Supplementary file 1 — Supplementary Material 1. [file 13065_2025_1678_MOESM1_ESM.docx]

**Supporting Information**

**Exploring 1,3,4-oxadiazolyl sulfide Derivatives as Antidiabetic candidates: Synthesis, Antioxidant activity, SAR-study, Molecular Docking, DFT-insights**

Norhan A. Khalaf^1^, Gehad E. Said,^1,*^ Ehab Abdel-Latif^1^, Heba M. Metwally,^1,*^

*^1^Department of Chemistry, Faculty of Science, Mansoura University, 35516 Mansoura, Egypt.*

*** *Corresponding authors E-mail addresses:* [*hebama@mans.edu.eg*](mailto:hebama@mans.edu.eg) *(HMM),* [*gehadsaid@mans.edu.eg*](mailto:gehadsaid@mans.edu.eg) *(GES)*

**3. Experimental**

***3.1. Materials***

All reagents and chemicals were purchased from commercial sources and used without further purification. Potassium carbonate (99%) was obtained from Merck. Acetone and absolute ethanol (99%) were obtained from Sigma-Aldrich.

***3.2. Chemistry general remarks:***

All melting points were evaluated using electric Gallenkamp (Germany) apparatus. The IR was performed on a Bruker Invenio D FTIR spectrometer, Germany. The elemental microanalytical data (C, H, and N) were measured at the Microanalytical Unit, Cairo University, on a Vario, Elemental apparatus (Shimadzu), and the experimental results coincided with the calculated values of the proposed molecular structural formulas. Jeol Resonance (Japan) apparatus (500 MHz) and Bruker Avance III (Germany) apparatus (400 MHz) were utilized in the determination of ^1^H-NMR data using DMSO-*d*_6_ and CDCl_3_ as solvents. Chemical shifts are reported in ppm (*δ*) downfield from TMS as an internal standard. Electron impact mass spectra were recorded on 70 eV EI Ms-QP 1000 EX Shimadzu apparatus (Japan). TLC was used to monitor the reaction mixtures using silica gel-coated plates and irradiation with a UV-Lamp for visualization. Purity was measured by UV absorbance at 254 nm.

1. **^1^H NMR and ^13^C NMR spectrum of the synthesized 1,3,4-oxadiazolyl sulfide derivatives** **4a–h.**

**
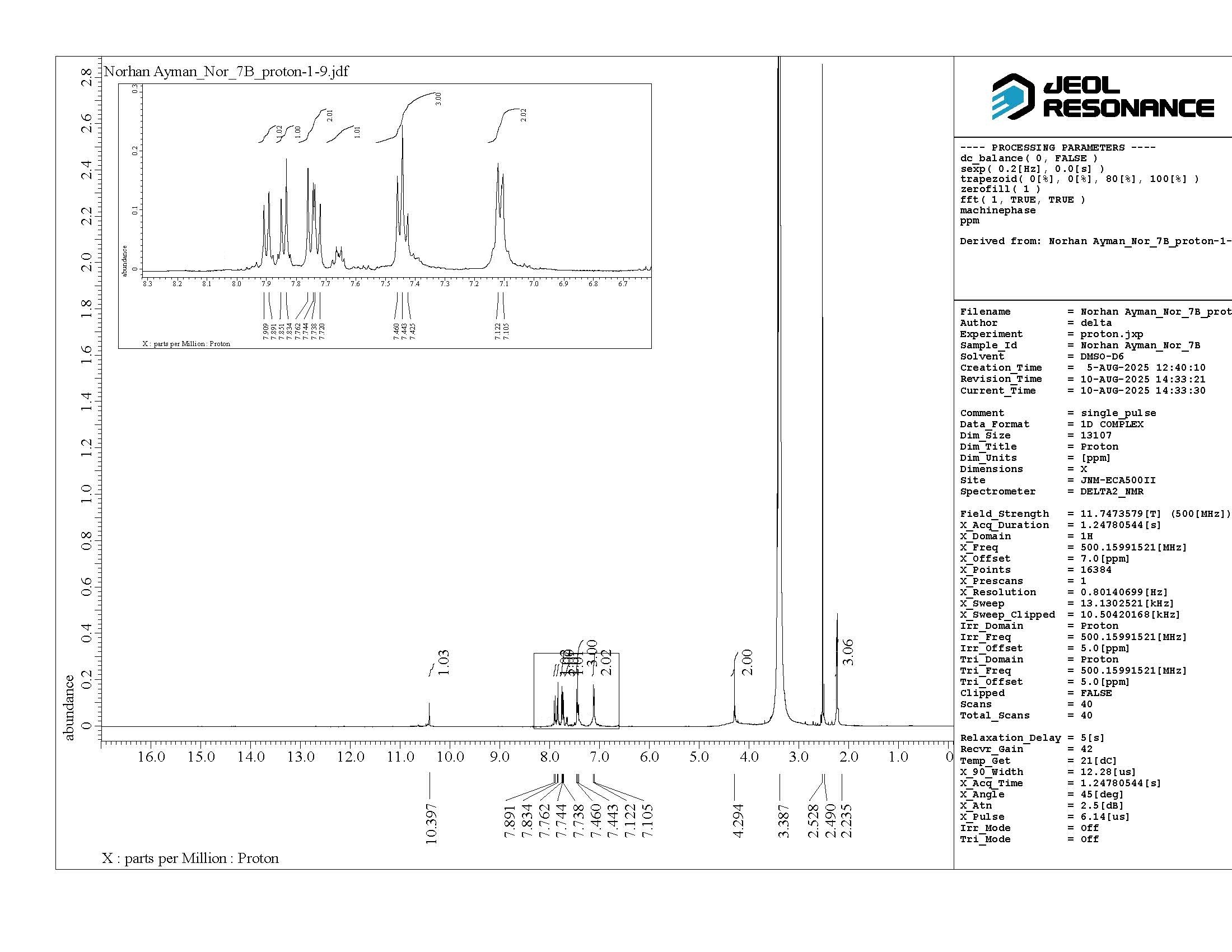
**

**Fig.S1.** **^1^H NMR spectrum of compound 4a**


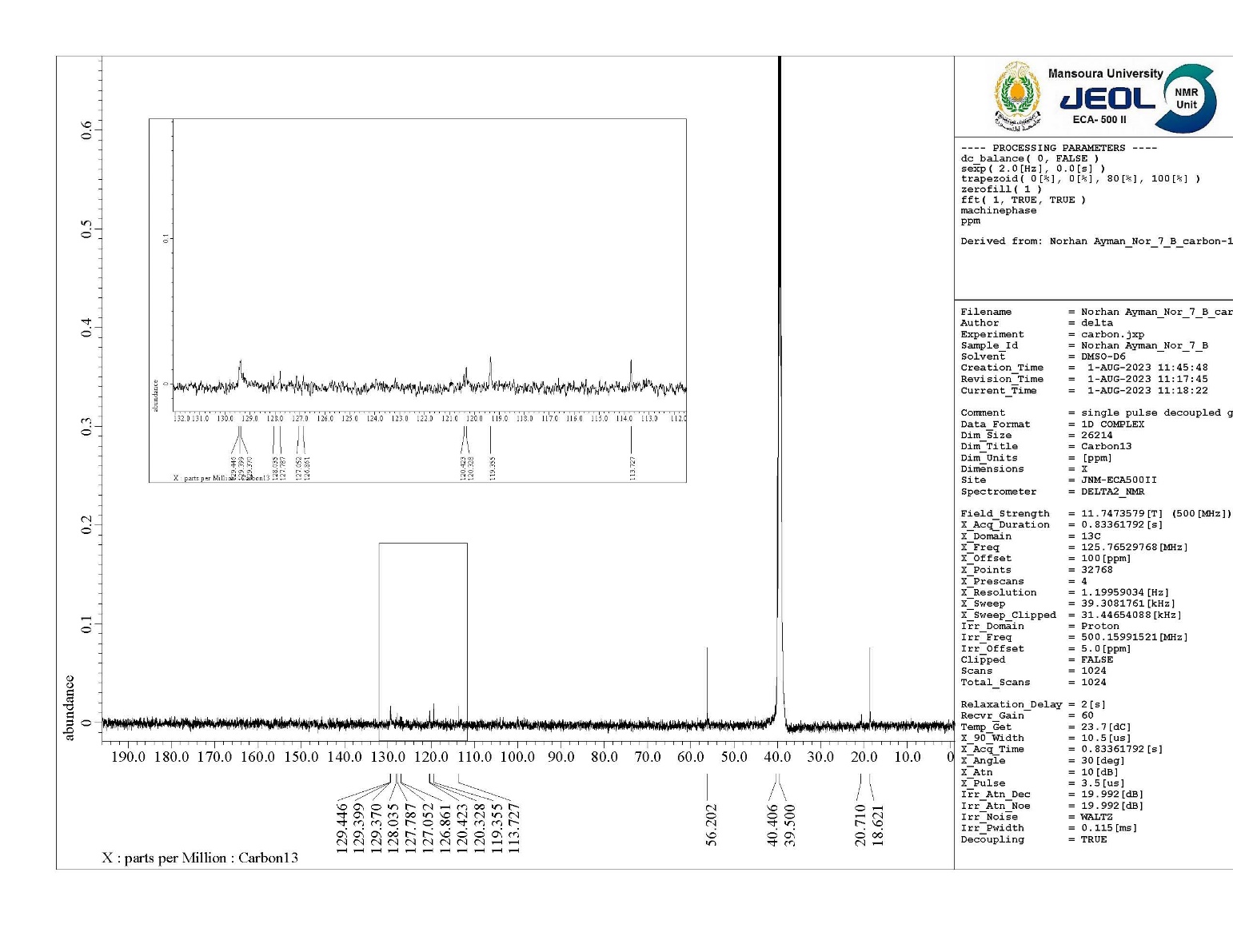

**Fig.S2.** **^13^CNMR spectrum of compound 4a**


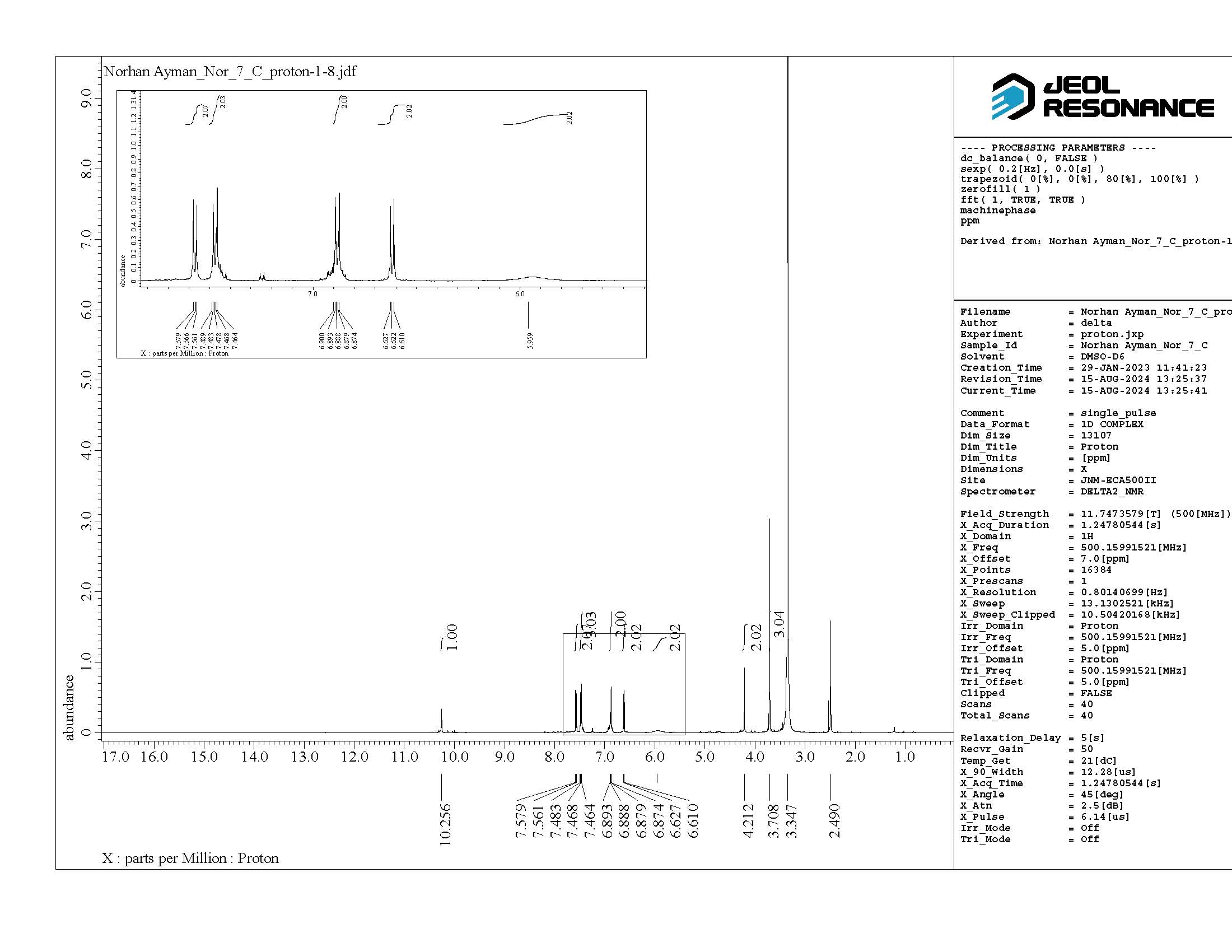

**Fig.S3.** **^1^H NMR spectrum of compound 4b**


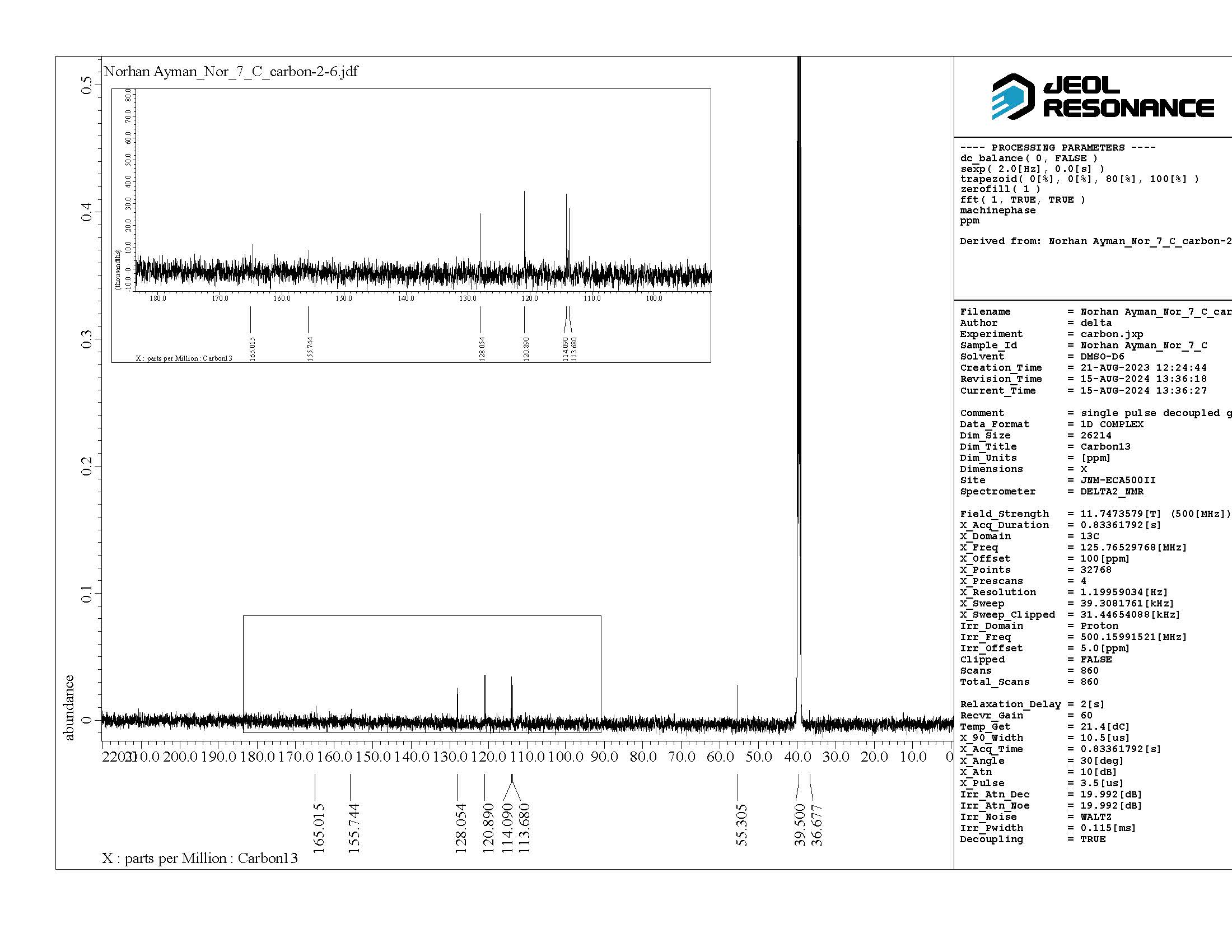

**Fig.S4.** **^13^CNMR spectrum of compound 4b**


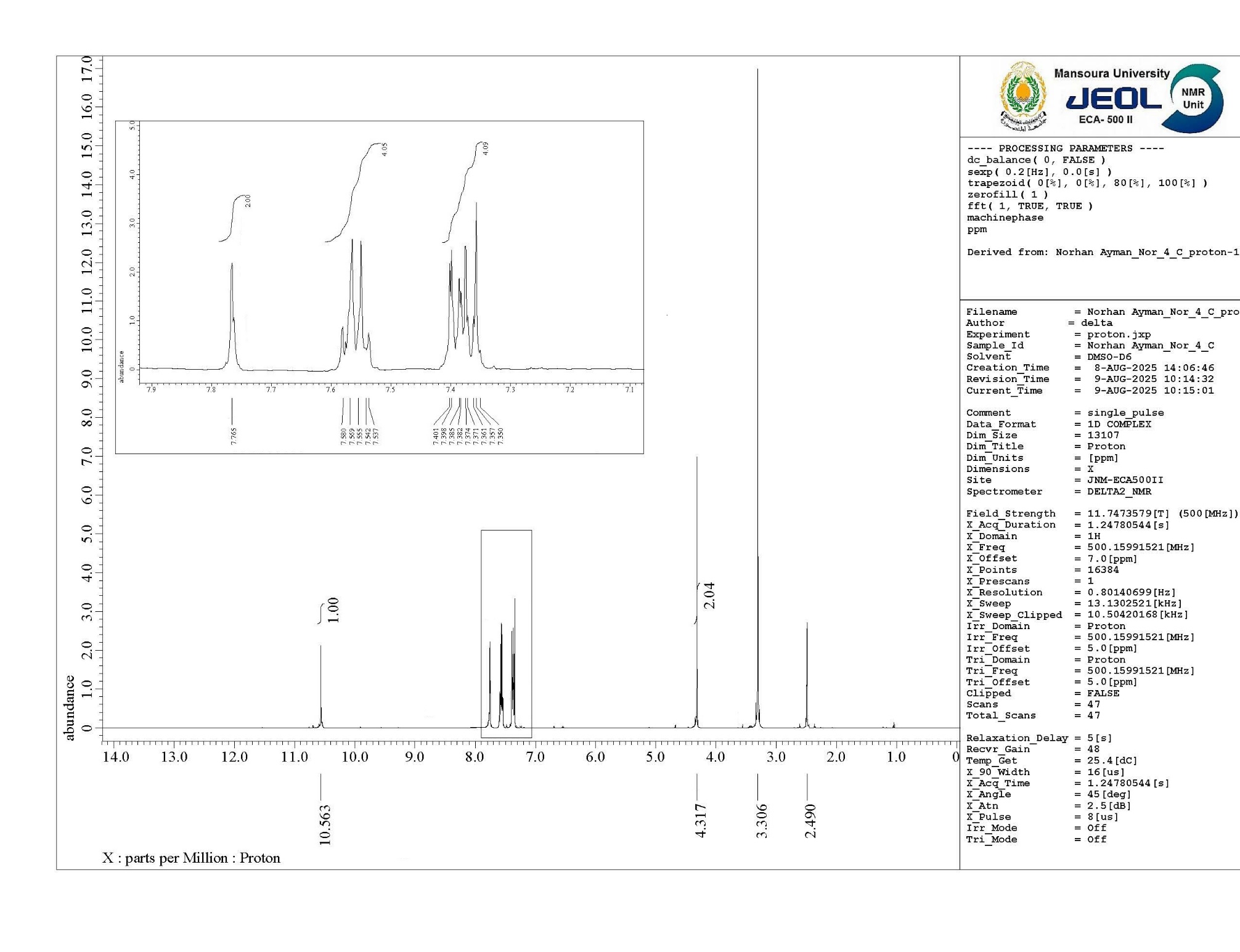

**Fig.S5.** **^1^H NMR spectrum of compound 4c**


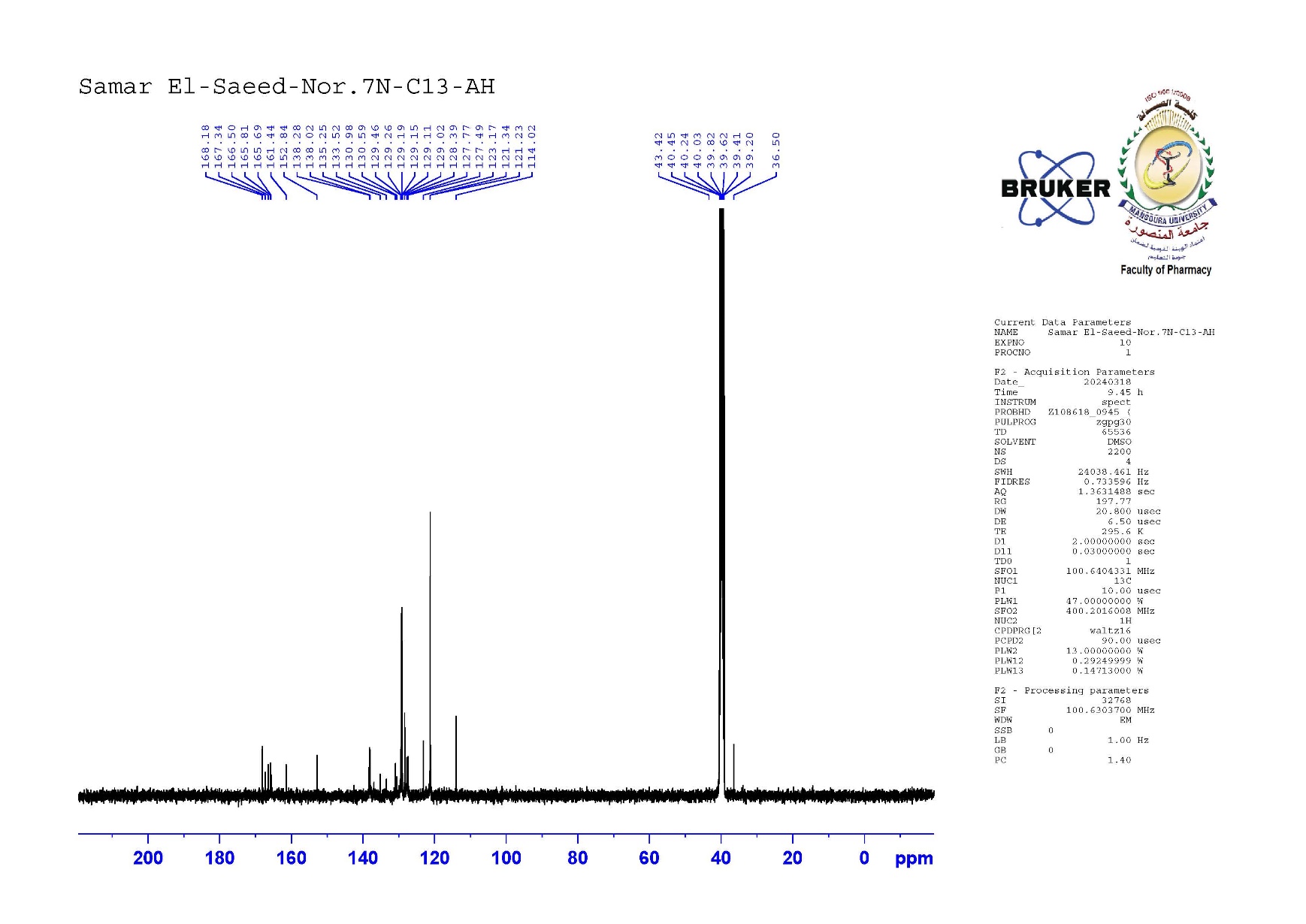

**Fig.S6.** **^13^CNMR spectrum of compound 4c**


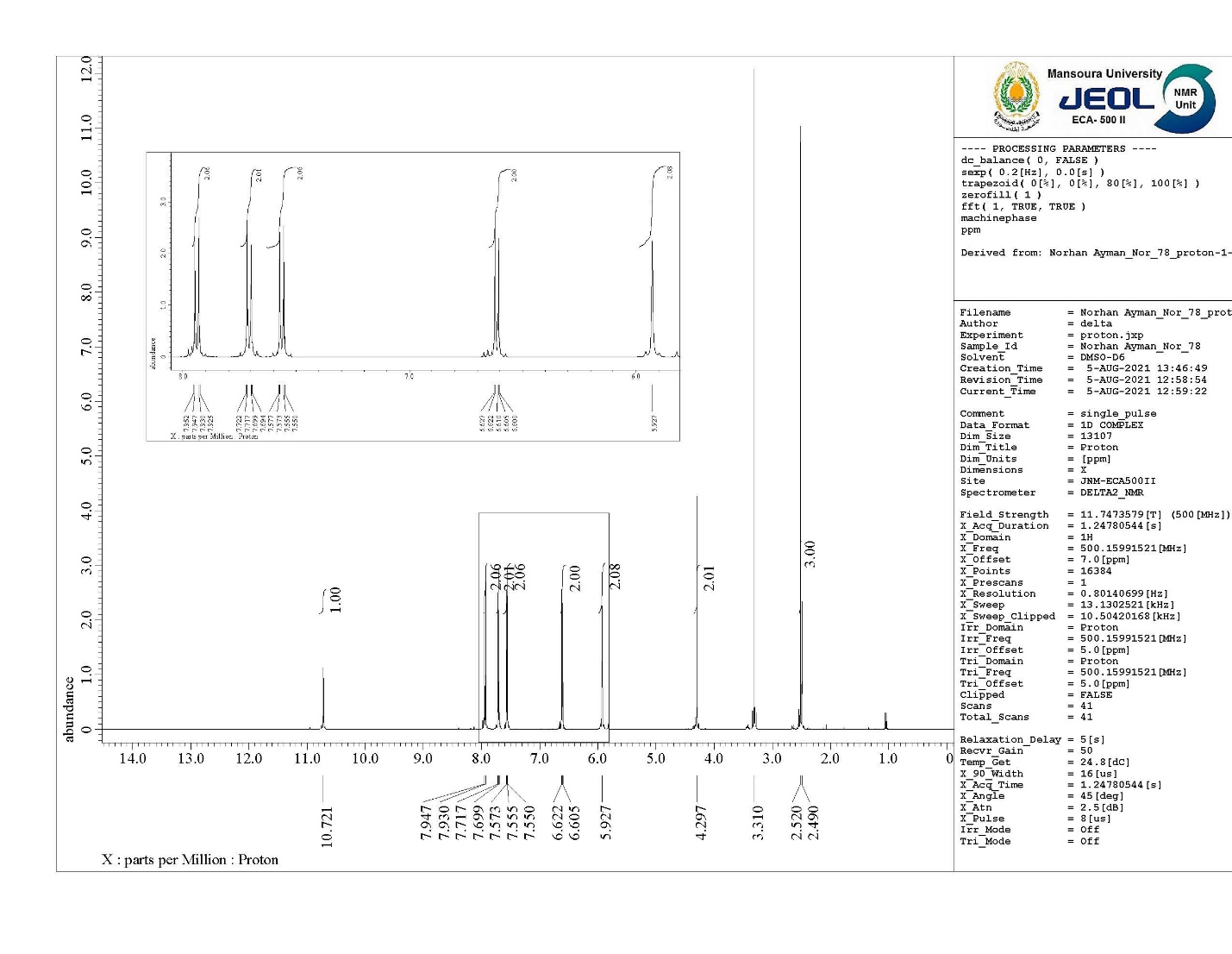

**Fig.S7.** **^1^H NMR spectrum of compound 4d**


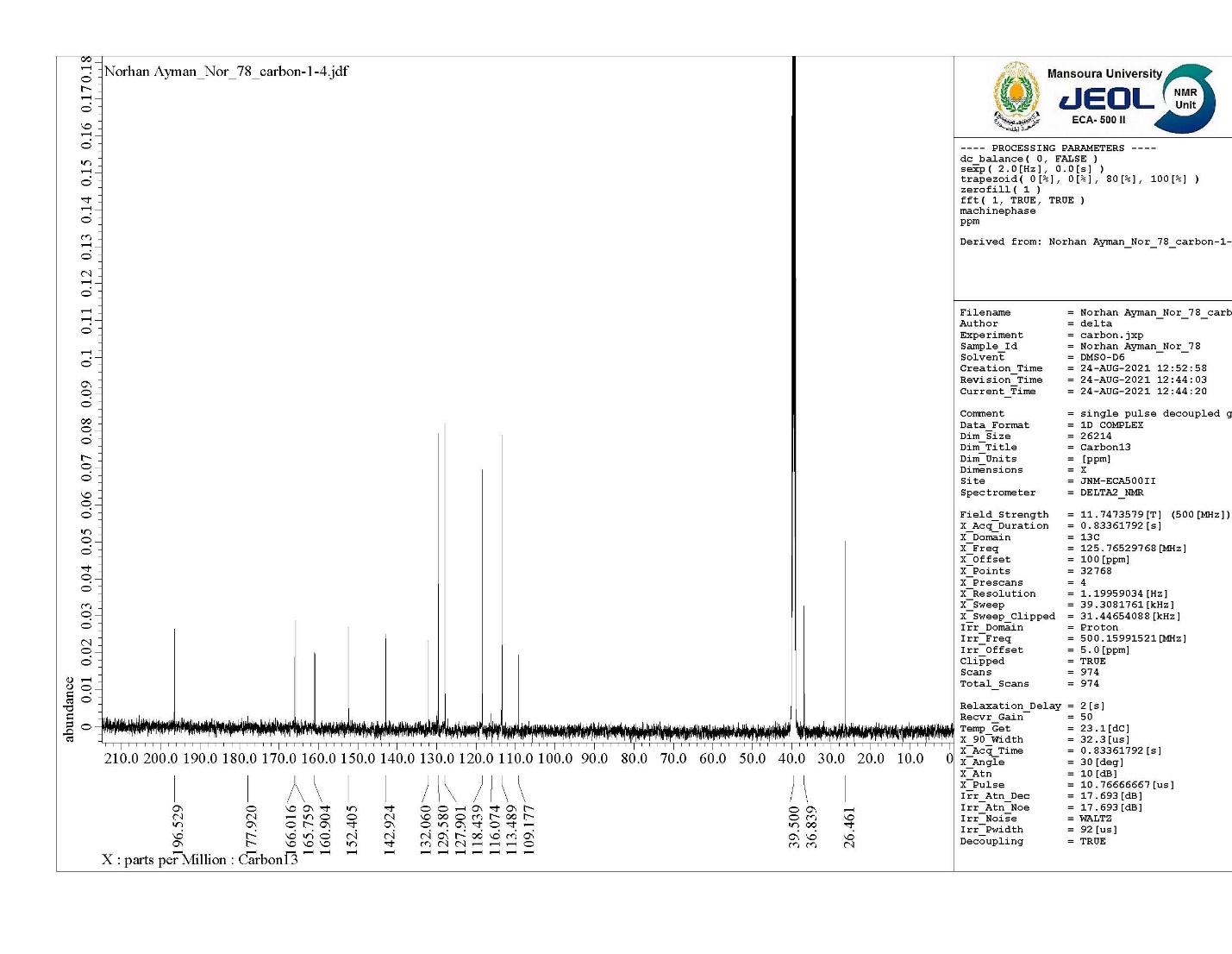

**Fig.S8.** **^13^CNMR spectrum of compound 4d**


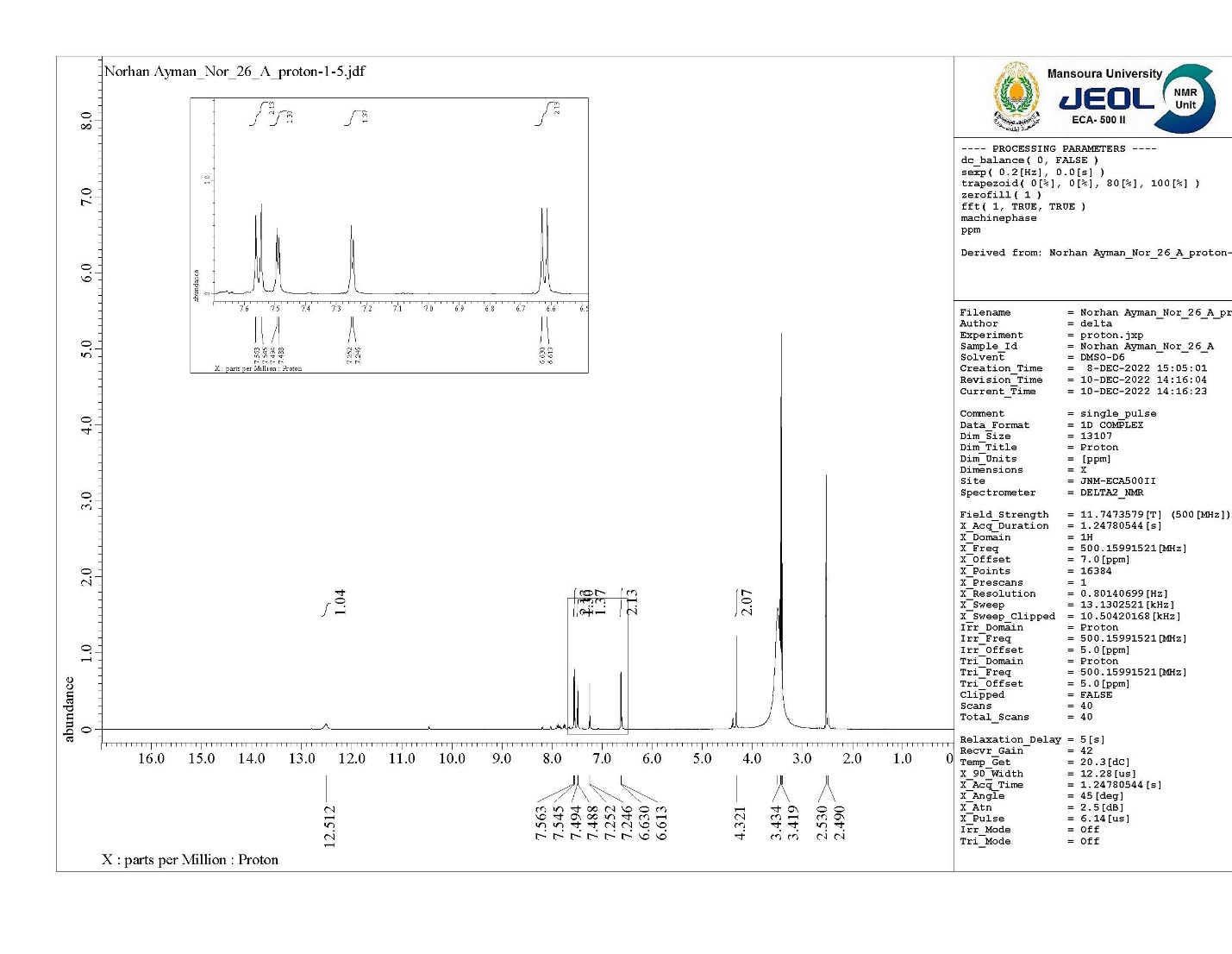

**Fig.S9.** **^1^H NMR spectrum of compound 4e**


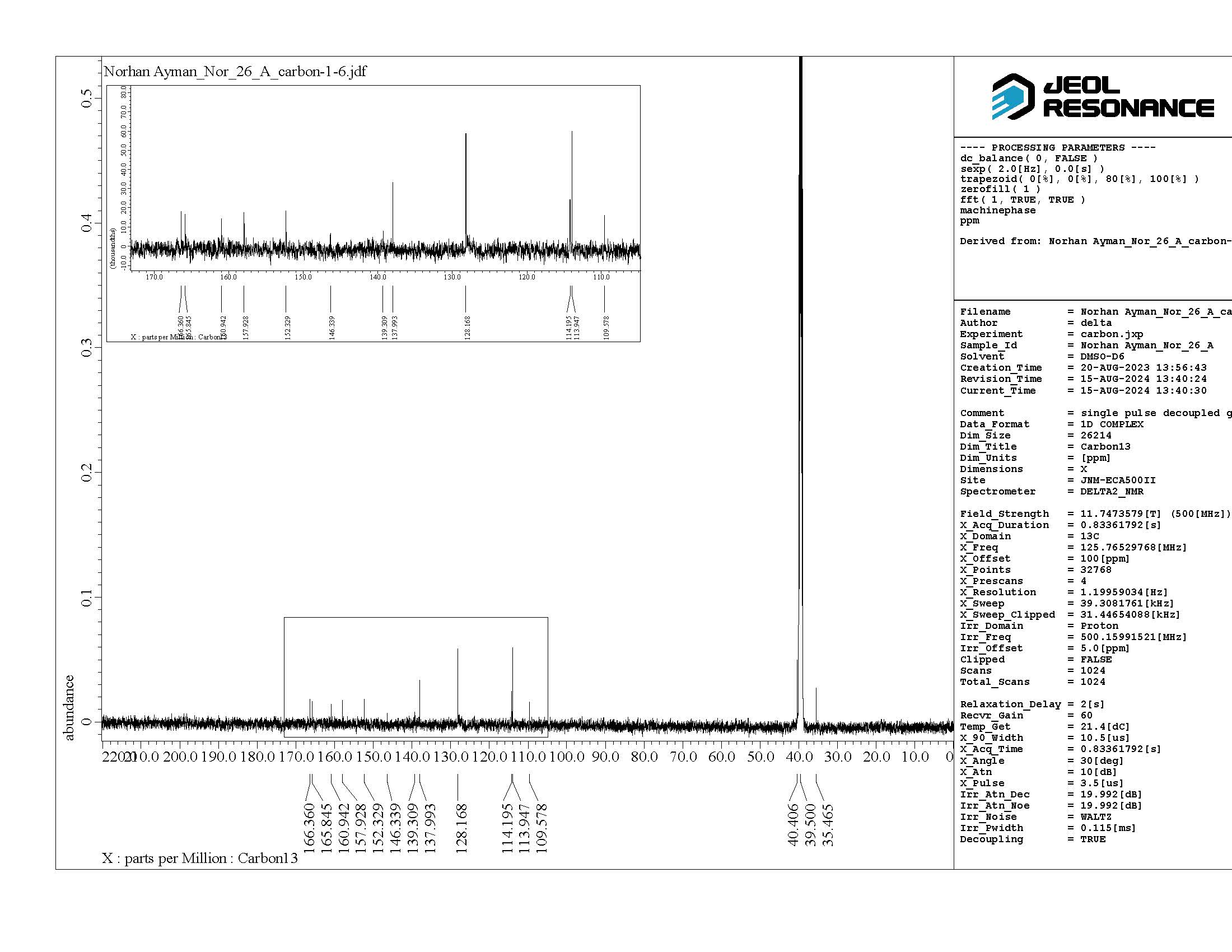

**Fig.S10.** **^13^CNMR spectrum of compound 4e**


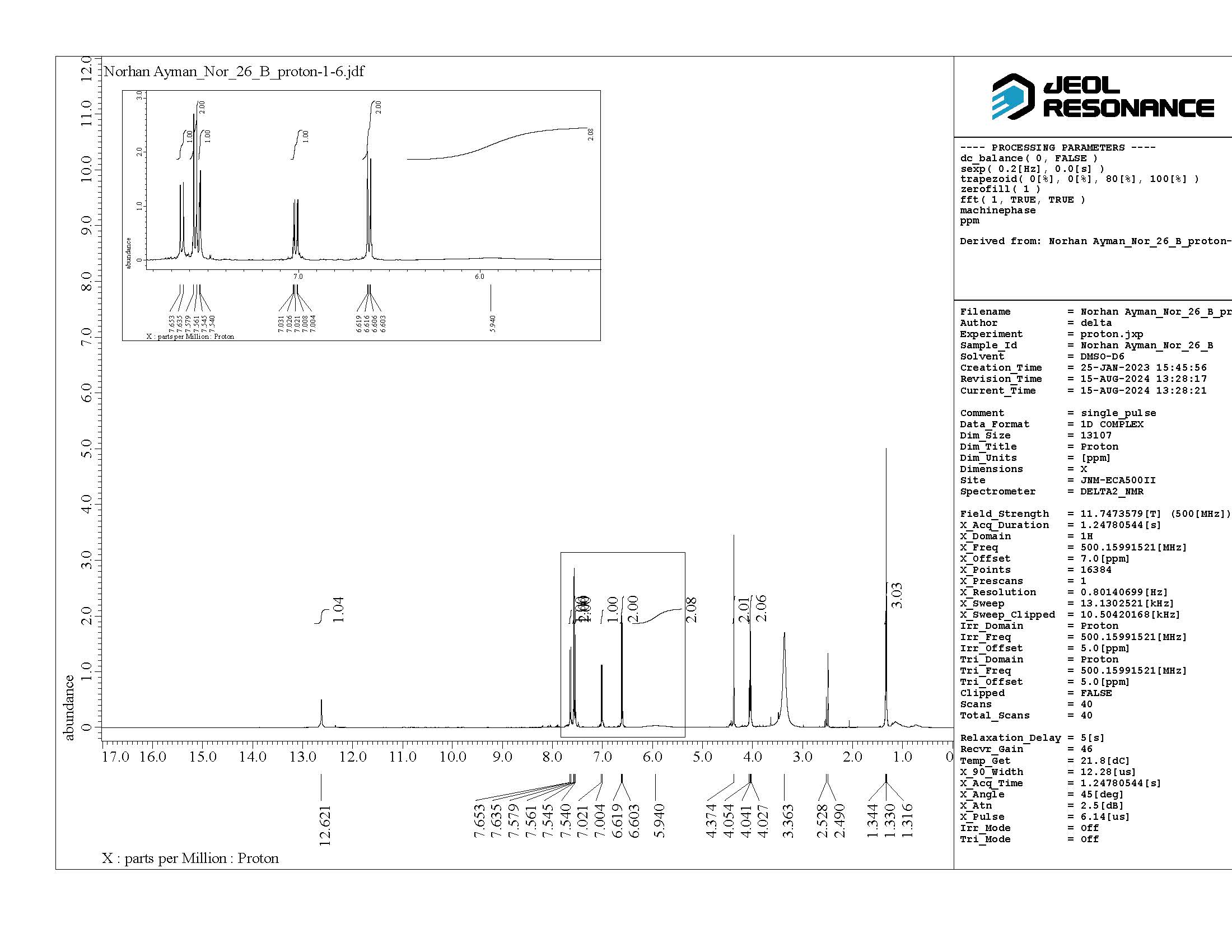

**Fig.S11.** **^1^H NMR spectrum of compound 4f**


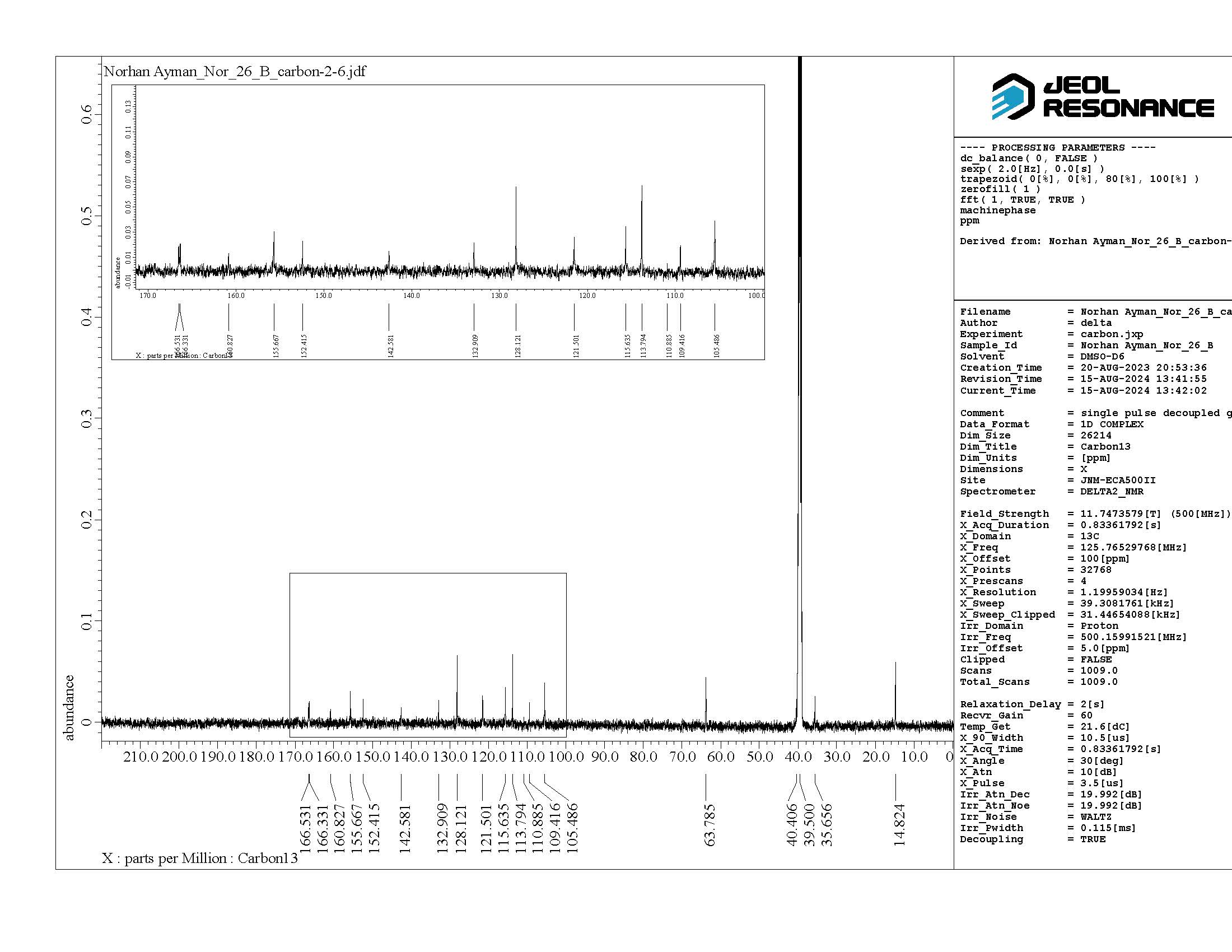

**Fig.S12.** **^13^CNMR spectrum of compound 4f**


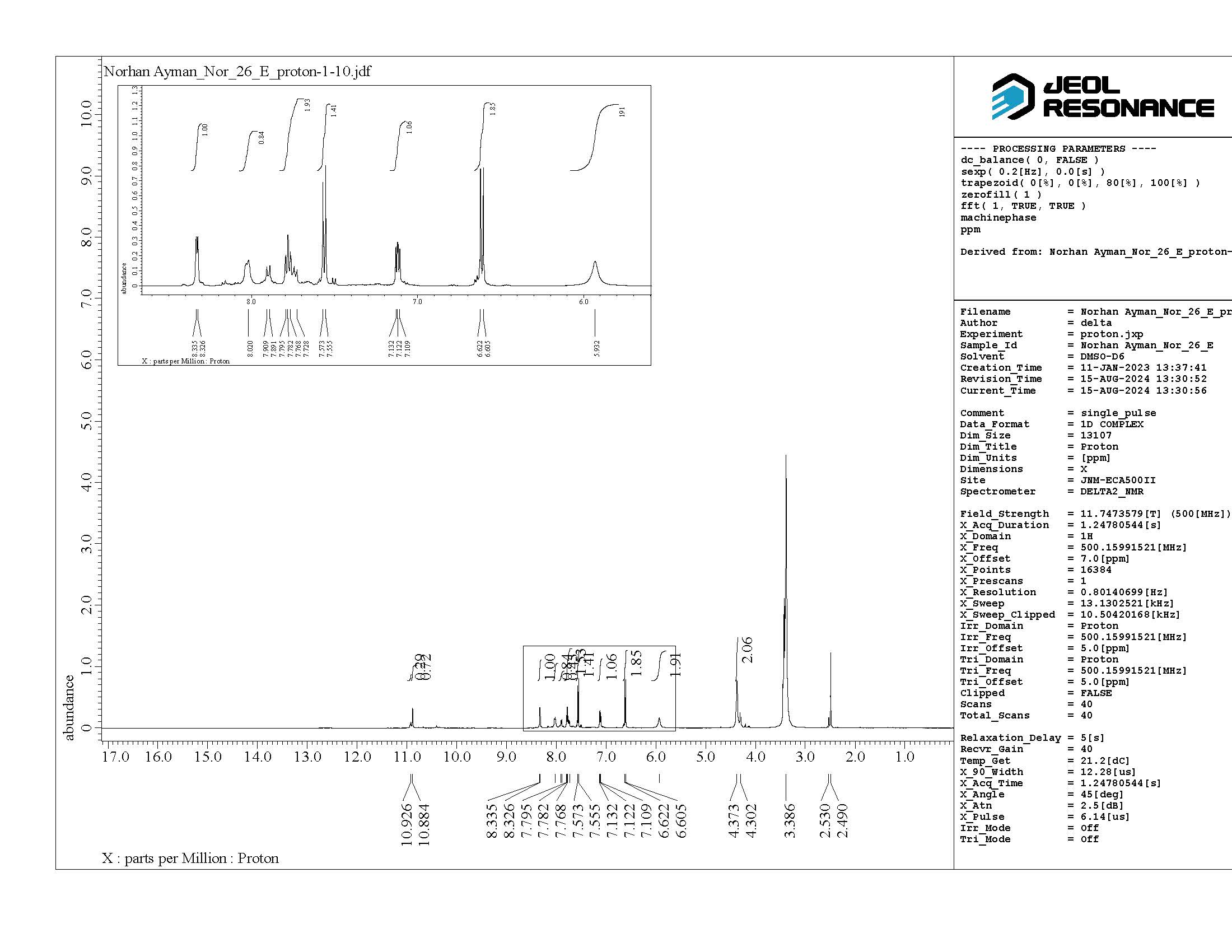

**Fig.S13.** **^1^H NMR spectrum of compound 4g**


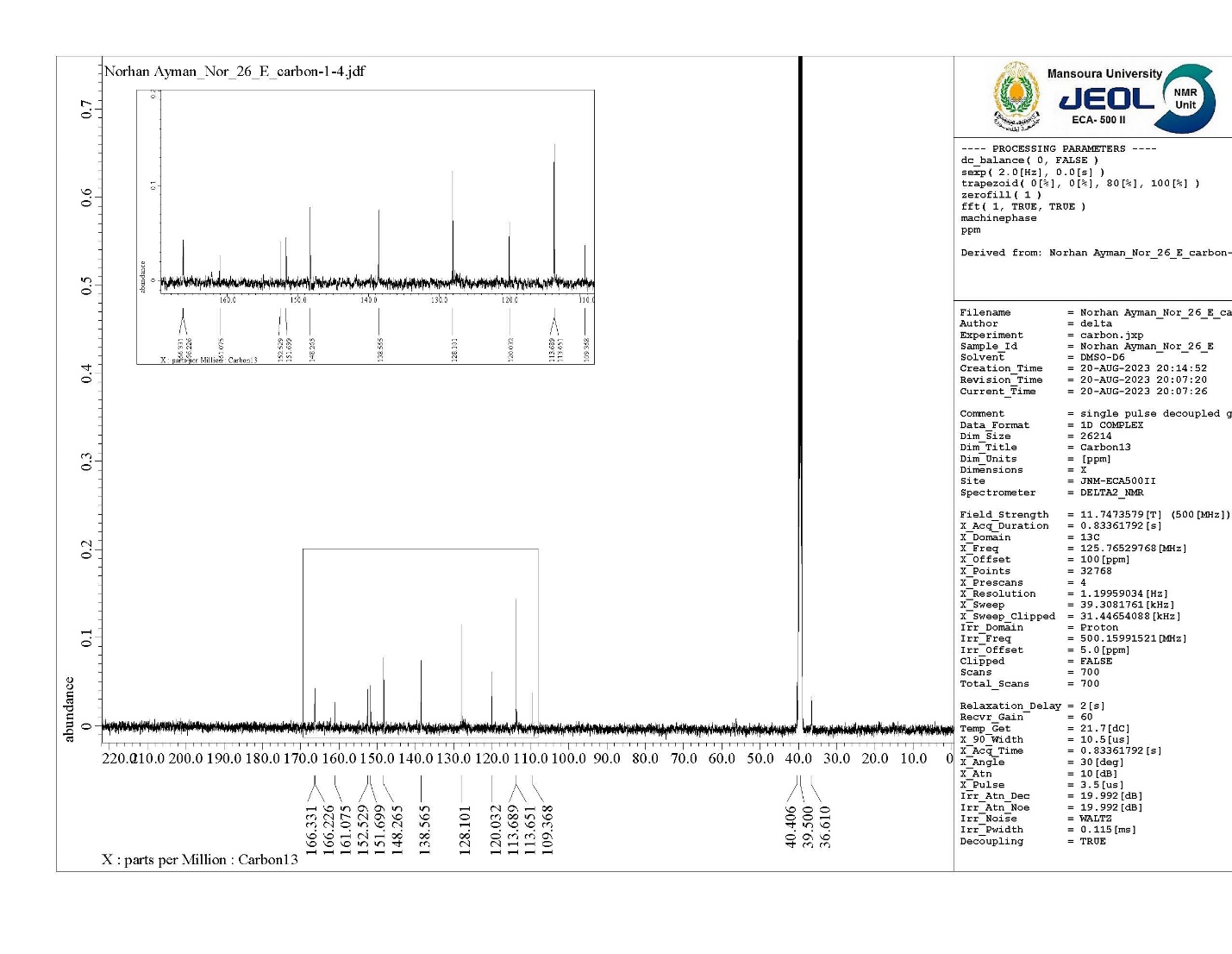

**Fig.S14.** **^13^CNMR spectrum of compound 4g**


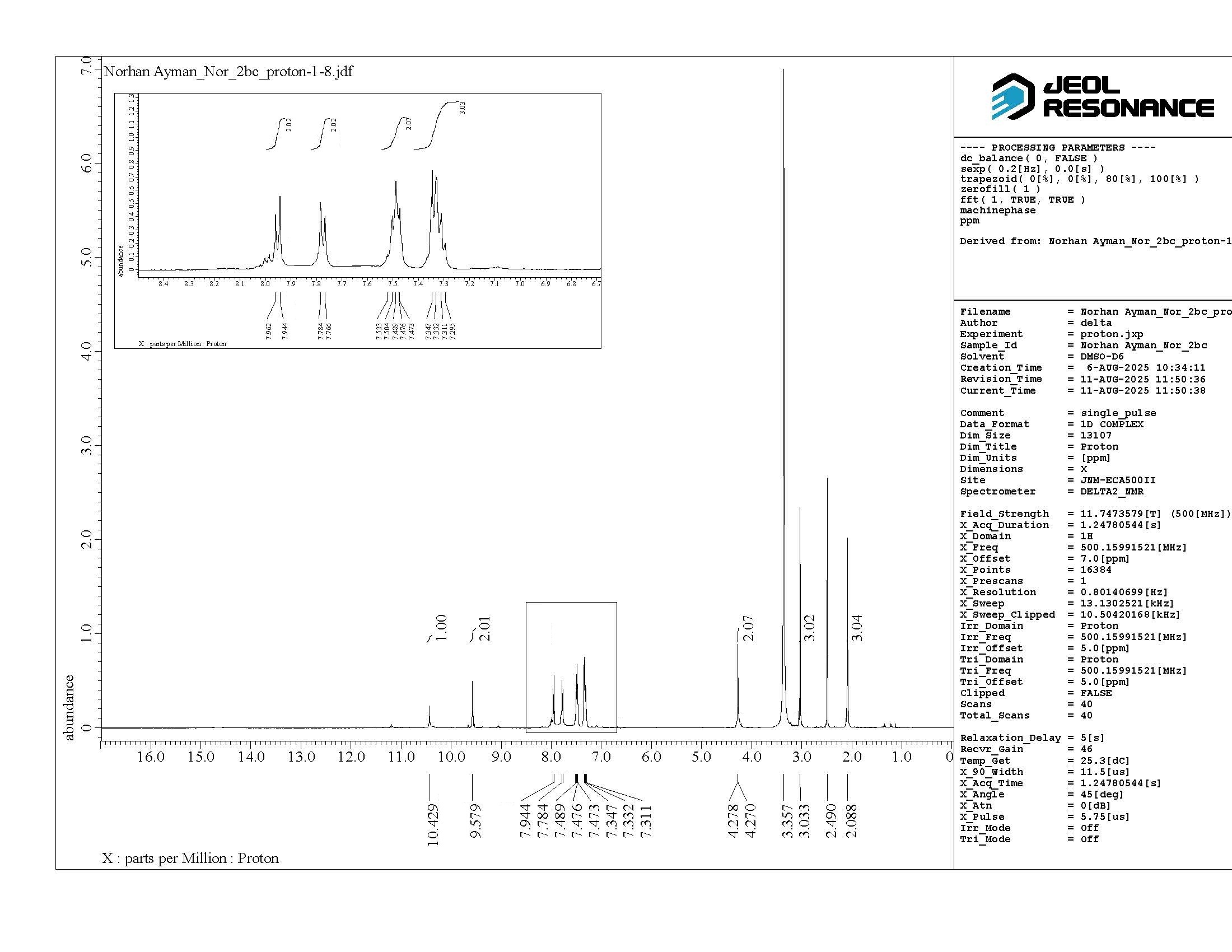

**Fig.S15.** **^1^H NMR spectrum of compound 4h**


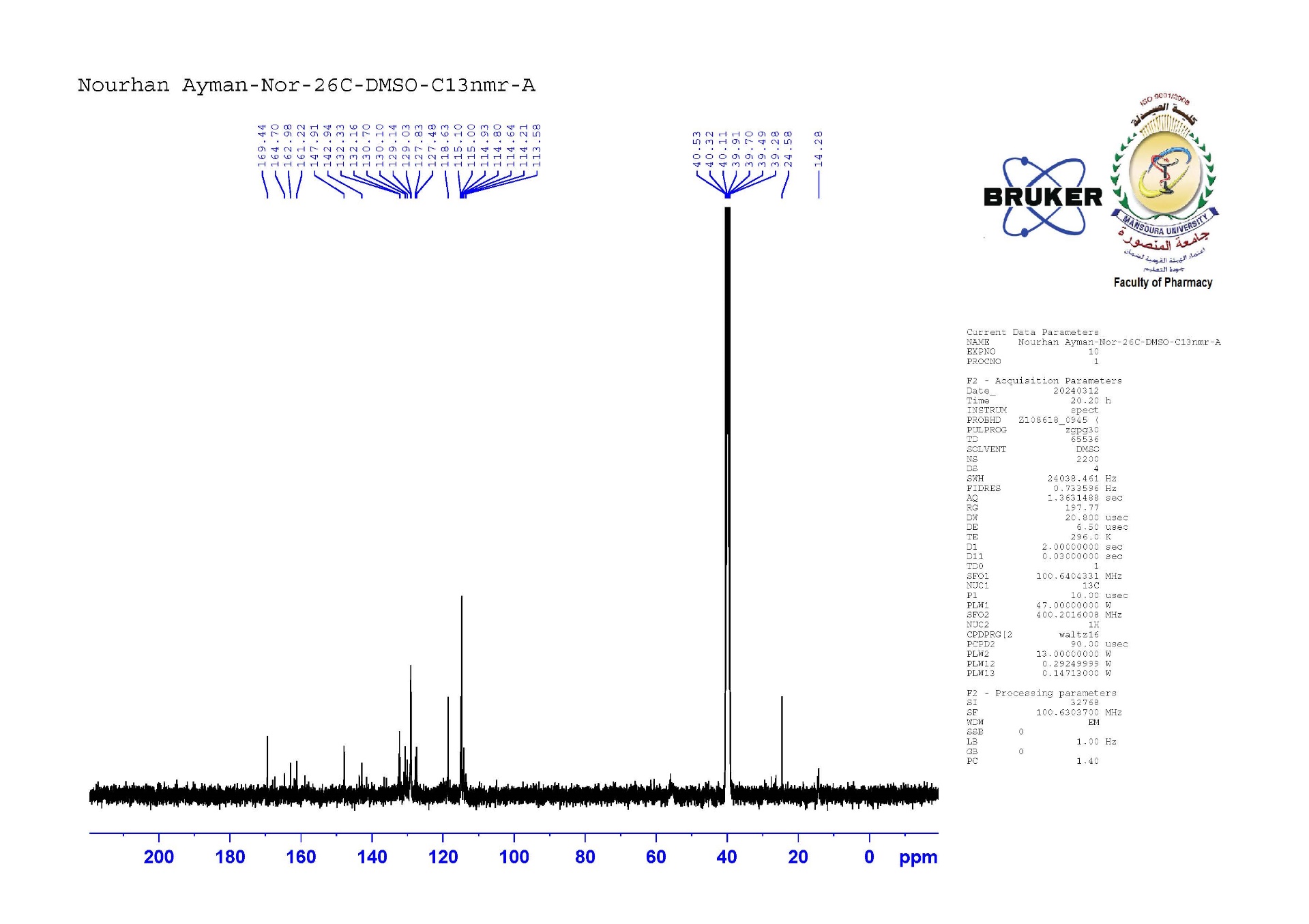

**Fig.S16.** **^13^CNMR spectrum of compound 4h**

1. **Purity of the tested compounds**

| 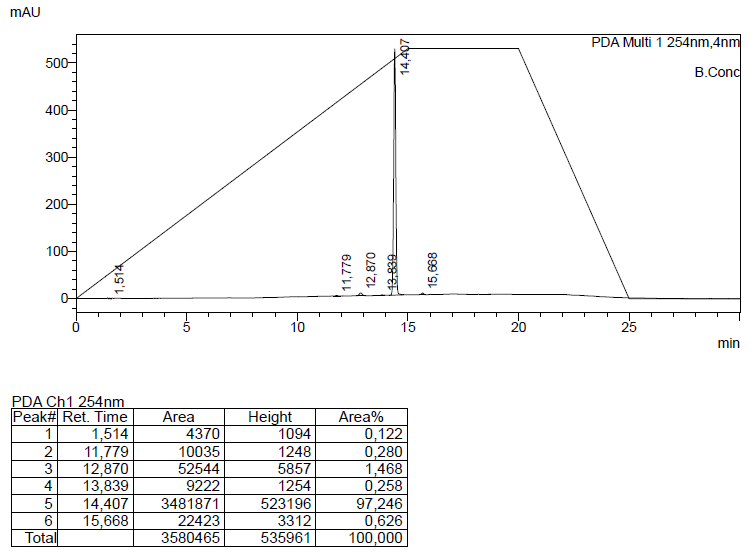  **4a** | 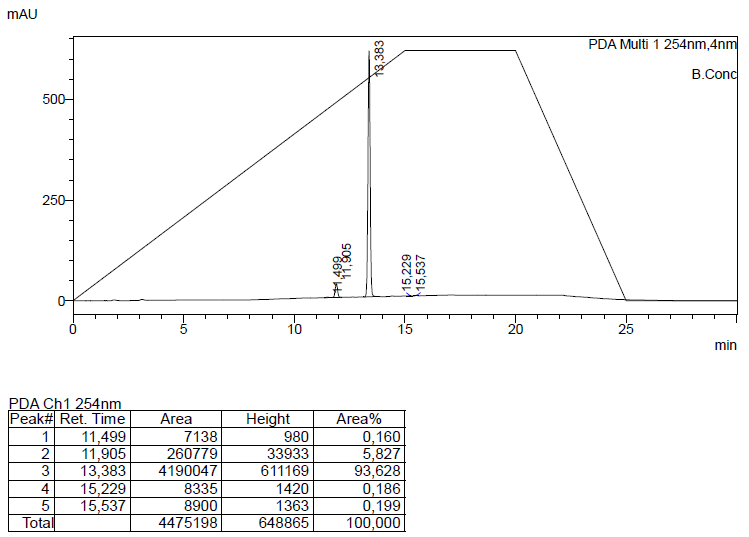  **4b** |
| --- | --- |
| **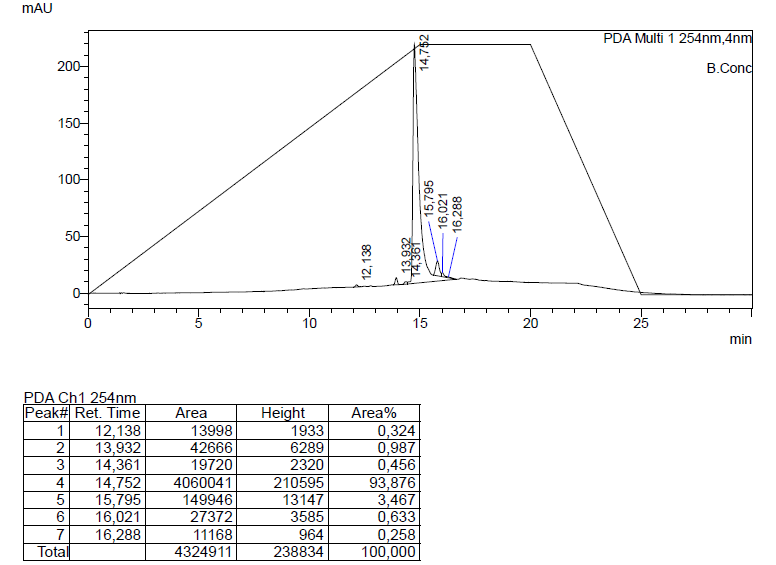**  **4c** | **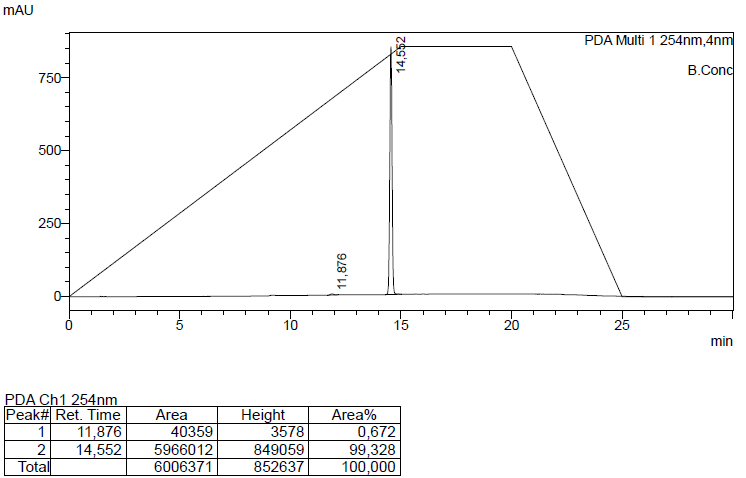**  **4d** |
| **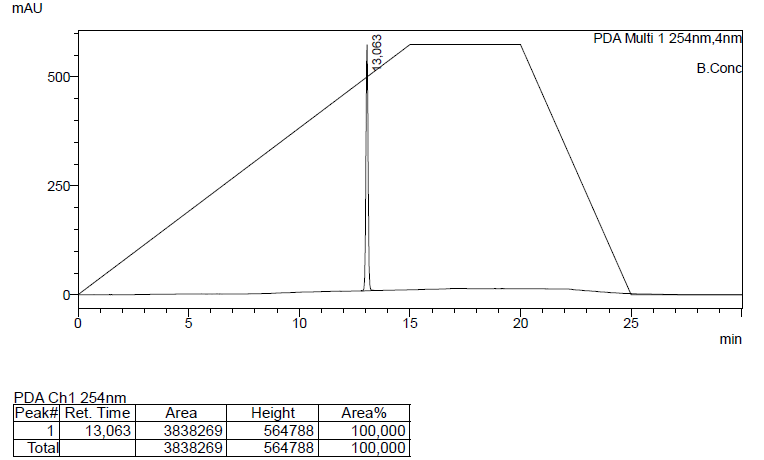**  **4e** | **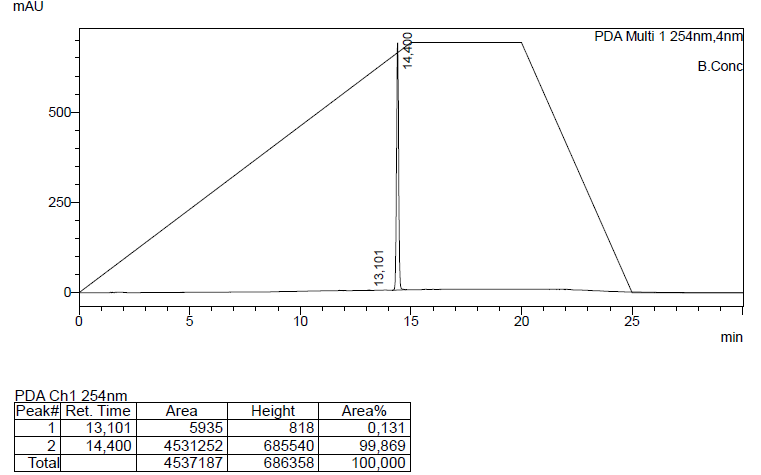**  **4f** |
| **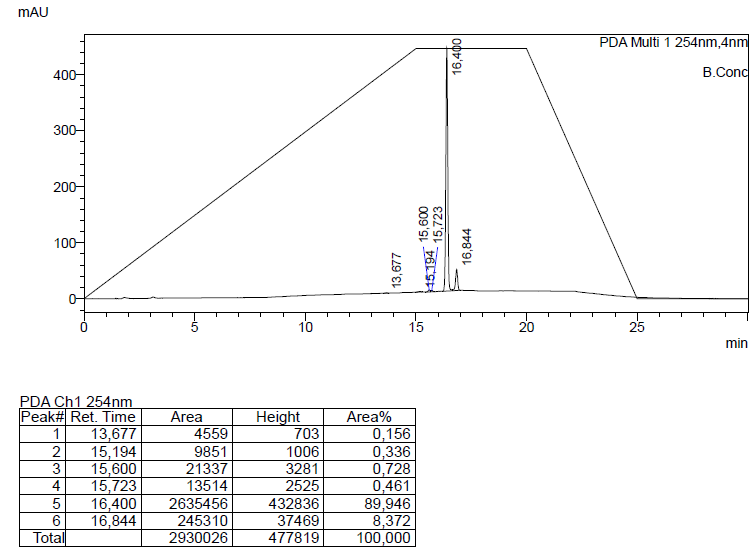**  **4g** | **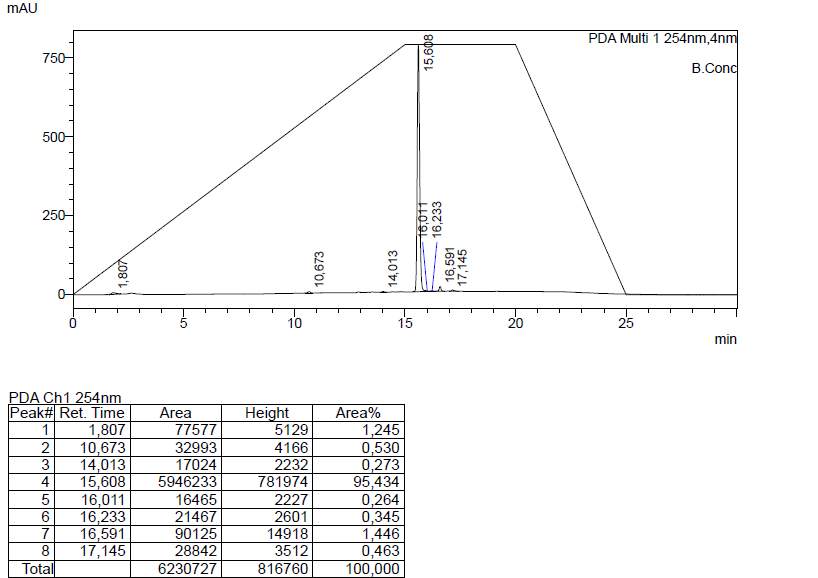**  **4h** |

**Fig. S17: Purity of the oxadiazole derivatives 4a-h**

1. **C,H,N elemental analysis**


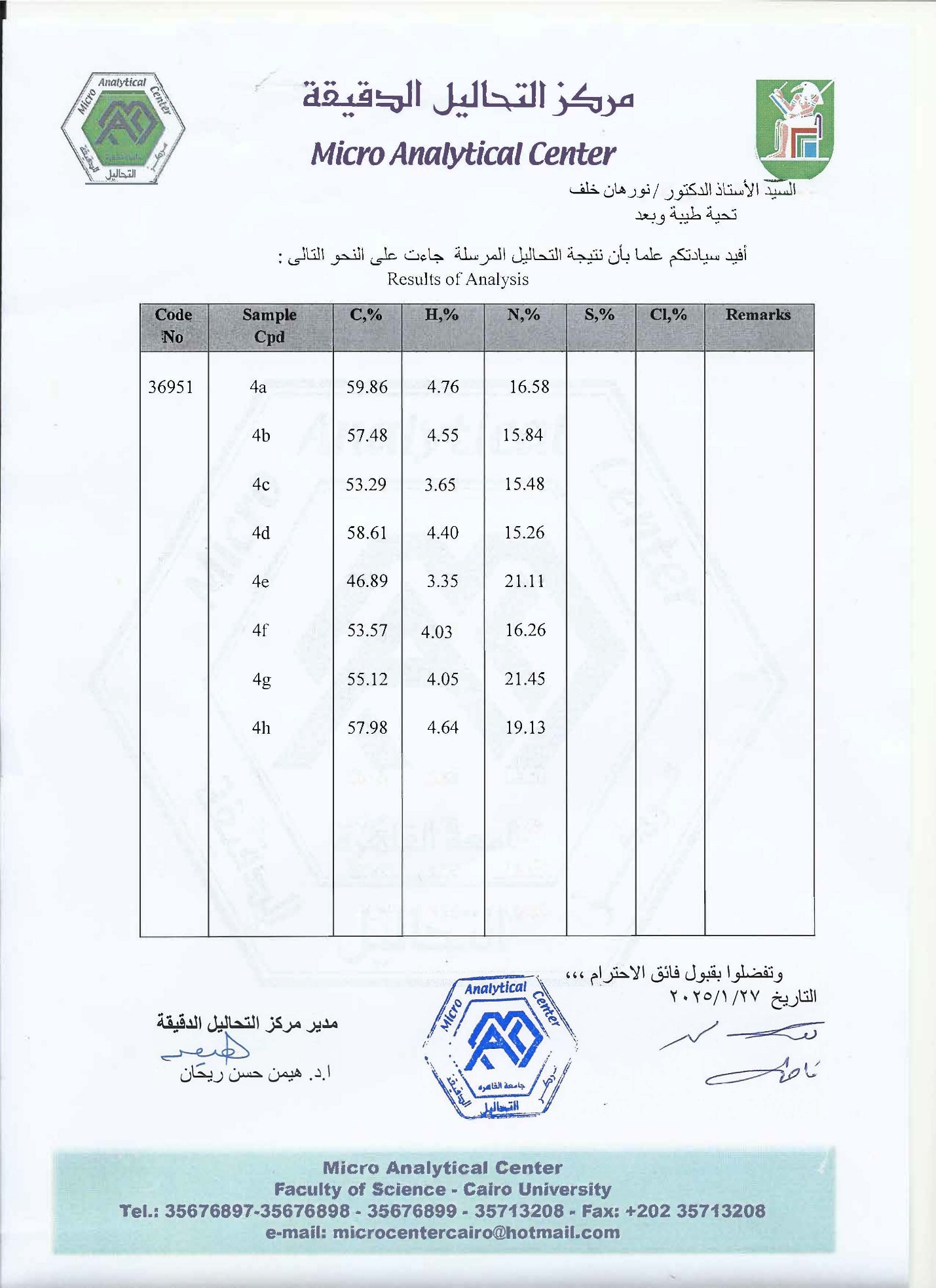


**Fig. S18: Elemental Analysis of the synthesized compounds**

**3.3. In vitro antioxidant (DPPH and ABTS) radical scavenging activities**

**3.3.1 Evaluation of antioxidant activity by ABTS radical scavenging method**

ABTS radical scavenging activity was determined according to Re et al. (1999) with some modifications.2,20-azino-bis(3-ethylbenzothiazoline-6-sulphonicacid) (ABTS) was dissolved in water to 7mM concentration. ABTS radical cation (ABTS^.+^) was produced by reacting ABTS stock solution with 2.45mM potassium persulfate (final concentration) and allowing the mixture to stand in the dark at room temperature for 12–16 h before use. The ABTS^.+^ solution was diluted with water to an absorbance of 0.70(±0.02) at 734nm.The reaction mixture consisted of 0.07 mL of extract and 3mL of the ABTS radical.After incubation for 6min, absorbance was determined in spectrophotometer at 734nm. The antioxidant activity was calculated by using the following equation.

%Inhibition = (A _control_ – A _sample_) / A _control_ x 100

A _control_ = Absorbance of negative control at the moment of solution preparation

A _sample_ = Absorbance of sample after 6min

The EC_50_ values were calculated from the graph which represents the concentration of the sample required to scavenge 50% of the ABTS free radicals. The EC_50_ is often used to express the amount or concentration of extracts needed to scavenge 50% of the free radicals. ABTS are expressed as ug GAE/mL.

**3.3.2. Evaluation of antioxidant activity by DPPH radical scavenging method**

Free radical scavenging activity of different compounds were measured by 1, 1- diphenyl-2-picryl hydrazyl (DPPH). In brief, 0.1 mM solution of DPPH in ethanol was prepared. This solution (1 ml) was added to 3 ml. of different extracts in ethanol at different concentration (3.9, 7.8, 15.62, 31.25, 62.5, 125, 250, 500, 1000 μg/ml). Here, only those extracts are used which are Solubilize in ethanol and their various concentrations were prepared by dilution method. The mixture was shaken vigorously and allowed to stand at room temp for 30 min. then, absorbance was measured at 517 nm. by using spectrophotometer (UV-VIS milton roy). Reference standard compound being used was ascorbic acid and experiment was done in triplicate.16 The IC 50 value of the sample, which is the concentration of sample required to inhibit 50% of the DPPH free radical, was calculated using Log dose inhibition curve. Lower absorbance of the reaction mixture indicated higher free radical activity. The percent DPPH scavenging effect was calculated by using following equation:

DPPH scavenging effect (%) or

Percent inhibition = A0 - A 1 / A0 × 100.

Where A0 was the absorbance of control reaction and A1 was the Absorbance in presence of test or standard sample.

**ABTS inhibition**


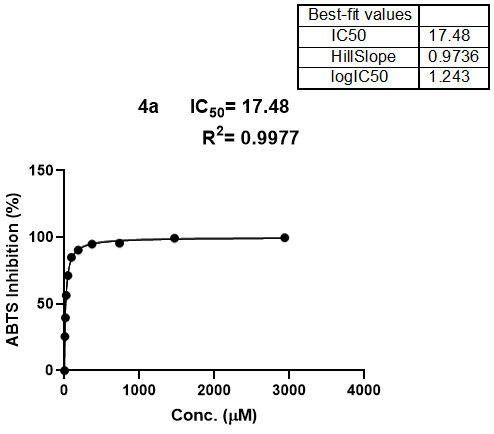
 AB
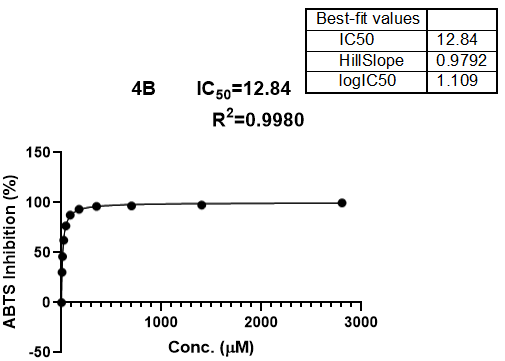

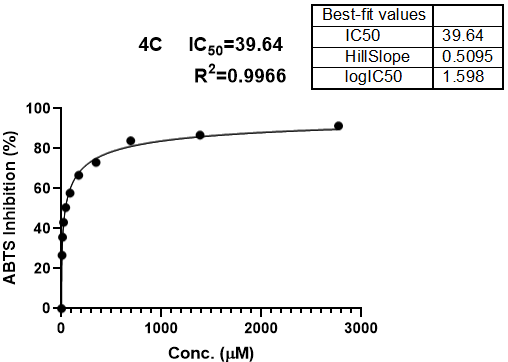

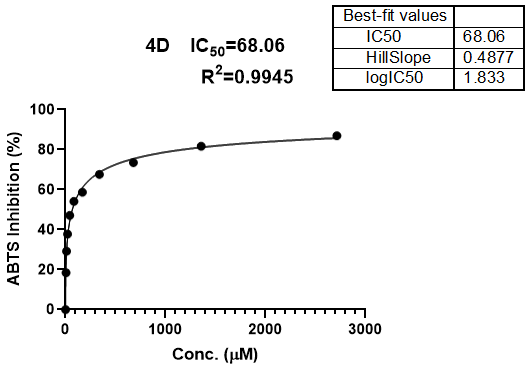


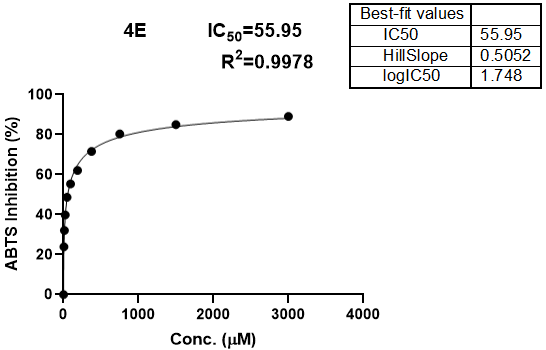

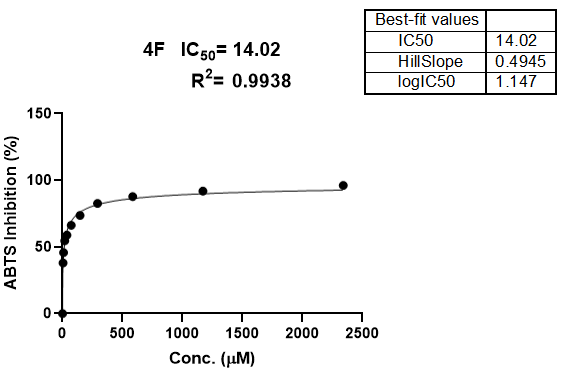


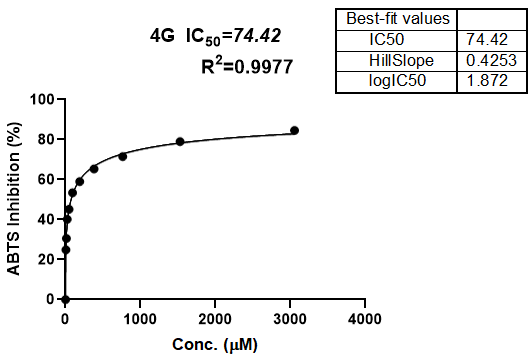

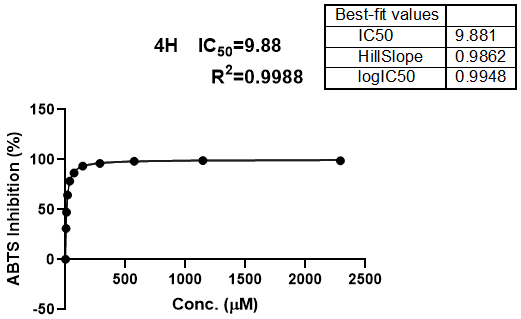


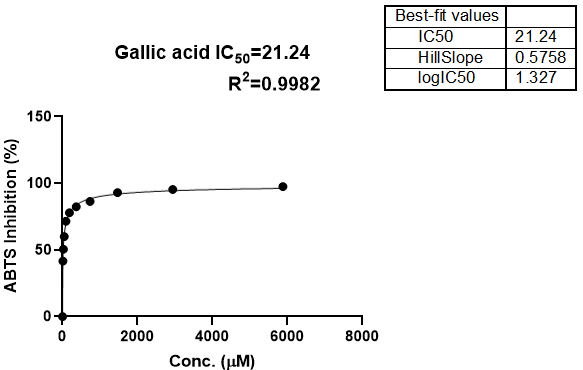


**Fig. S19 IC50 curves measured for the tested compounds against ABTS**

**DPPH inhibition**


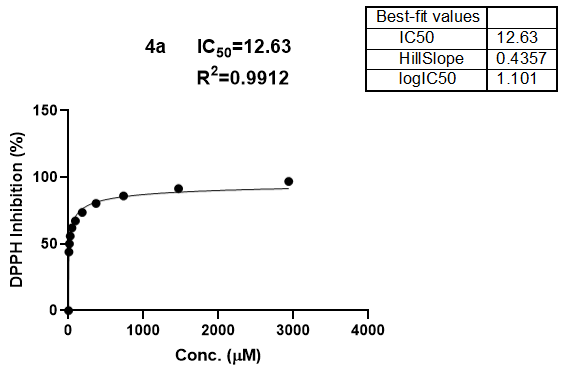

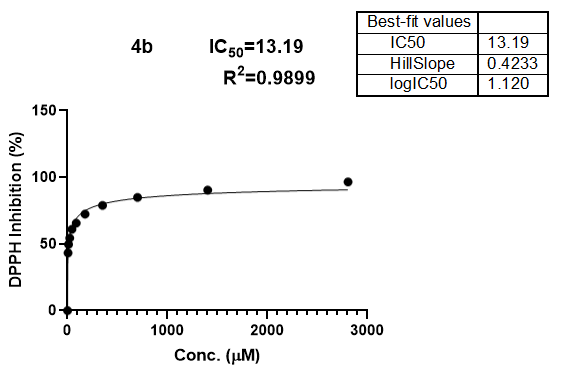


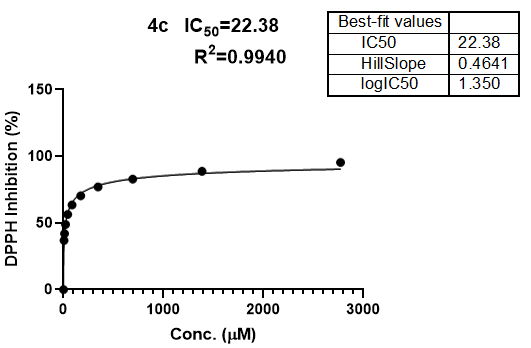

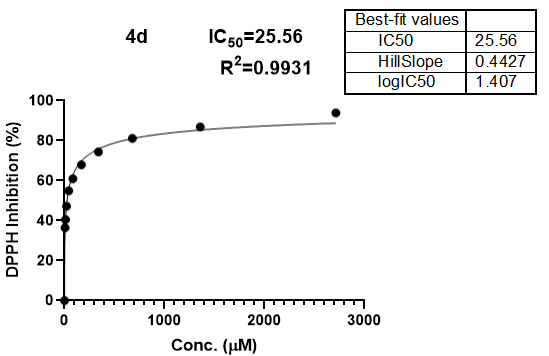


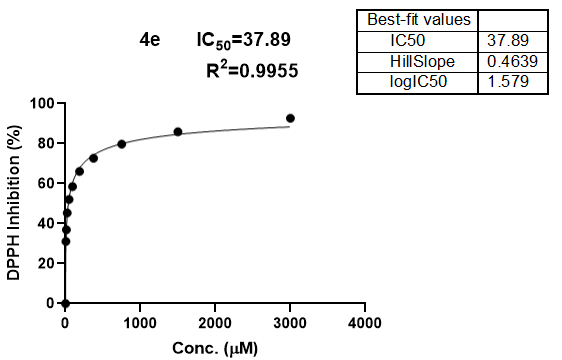

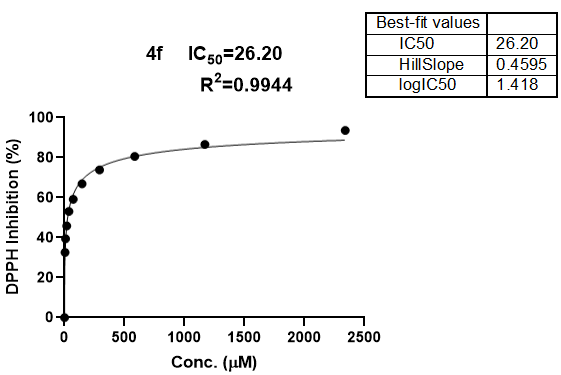


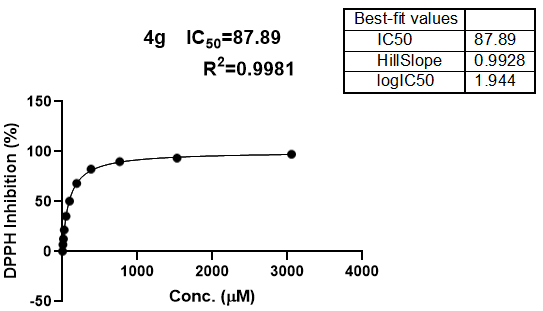

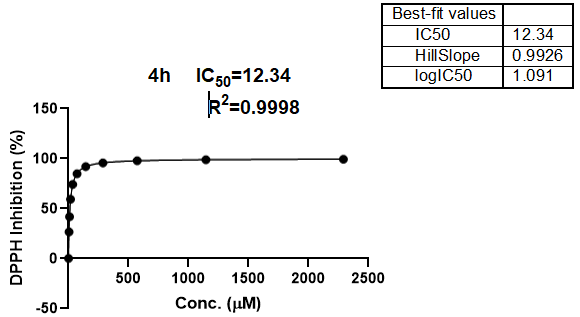


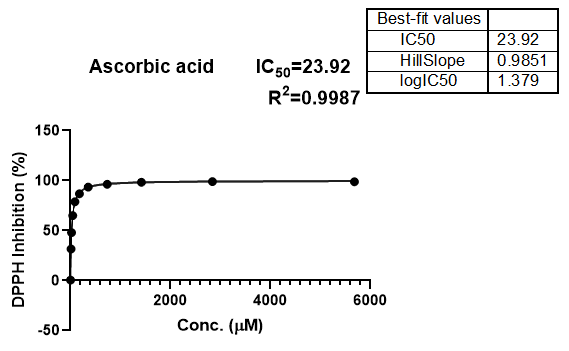


**Fig. S20 IC50 curves measured for the tested compounds against DPPH**

**Molecular docking**

Molecular docking simulations were carried out for the synthesized compounds to study their binding capability. Molecular Operating Environment (MOE, 2015.10) software was exploited for docking purpose. All minimizations were carried out using MOE till an RMSD of 0.05 kcal•mol^-1^Å^-1^ applying MMFF94x force field, and partial charges were automatically calculated. The X-ray co-crystallographic structure of α-glucosidase with the inhibitor acarbose (PDB ID: 3w37) was obtained from the RSCB protein data bank. Subsequently, the protein structure was prepared using Protonate 3D protocol with default options. Placement using Triangle Matcher placement method and scoring using London dG scoring function were selected. The active site in the protein structure was defined using the co-crystalized ligand, acarbose. The applied docking protocol was then validated by redocking of the cognate ligand (tolrestat) in the active site giving a docking pose with an energy score (S) = -7.35 kcal/mol and an RMSD of 0.5233 Å from the co-crystalized ligand pose.

The standard drug acarbose (docking energy = -9.99 kcal/mol) displayed greater hydrogen bond interactions with Asp232, Ala234, Asn237, Arg552, Asp568, and His626 in the target’s active site as it contains a number of hydroxy and hydroxy methyl functional groups. Moreover, standard drug acarbose formed a number of hydrophobic interactions with Ile233, Phe236, Trp329, Trp432, Met470, and Phe601.

**Docking poses of the target compounds in α-glycosidase active site**

| 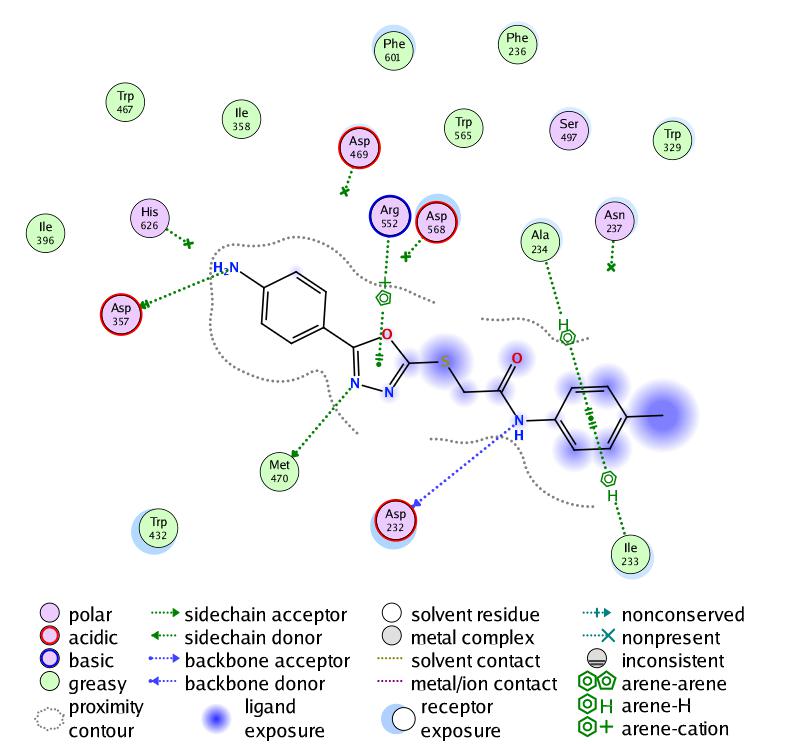 | 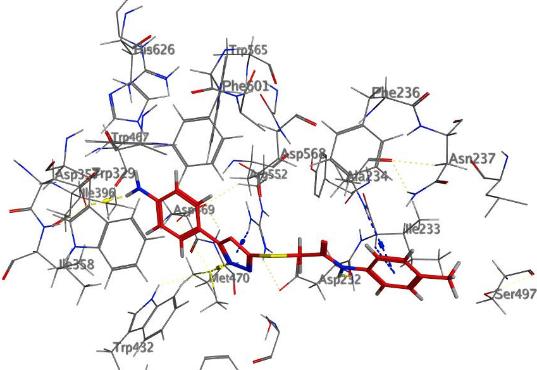 |
| --- | --- |
| **2D** | **3D** |
| 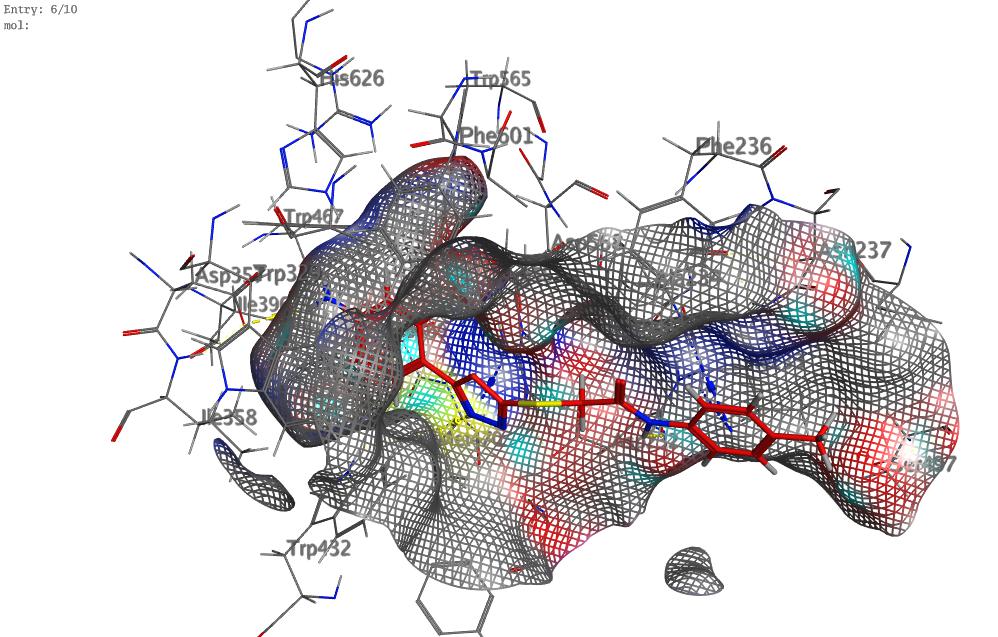 | |
| **Surface map** | |

**Figure S21**: The binding interaction of 1,3,4-oxadiazole **4a** with (PDB ID: 3W37).

| 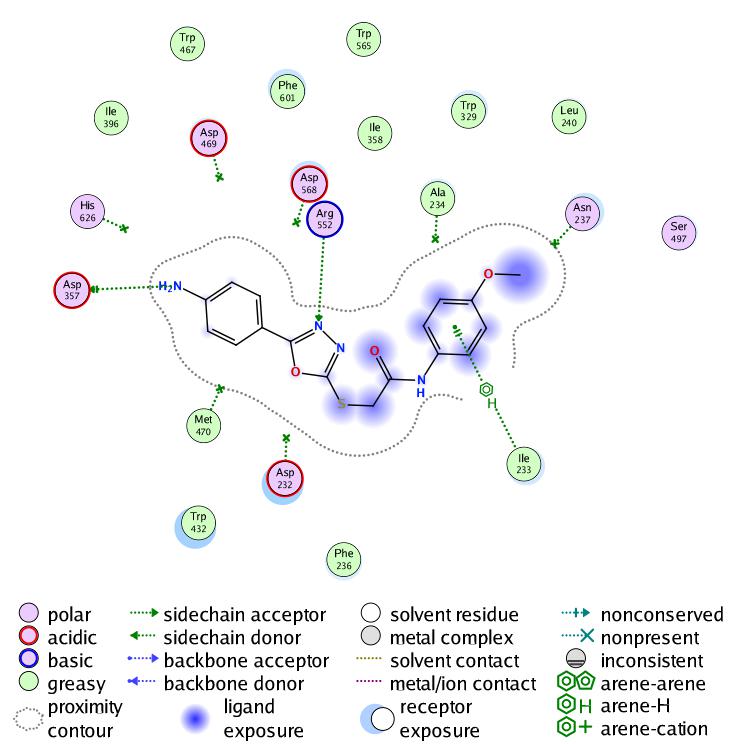 | 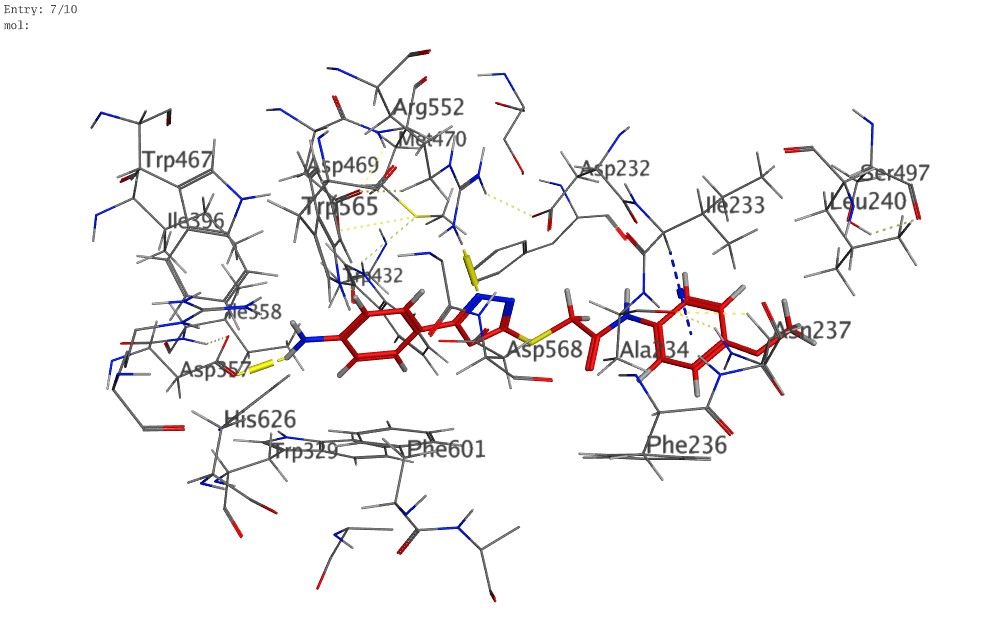 |
| --- | --- |
| **2D** | **3D** |
| 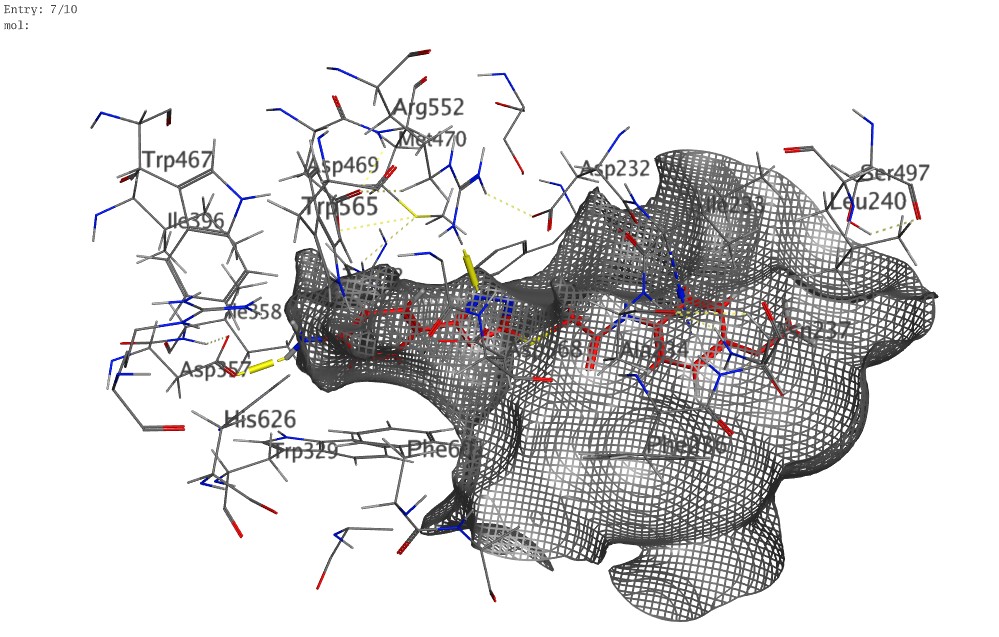 | |
| **Surface map** | |

**Figure S22**: The binding interaction of 1,3,4-oxadiazole **4b** with (PDB ID: 3W37).

| 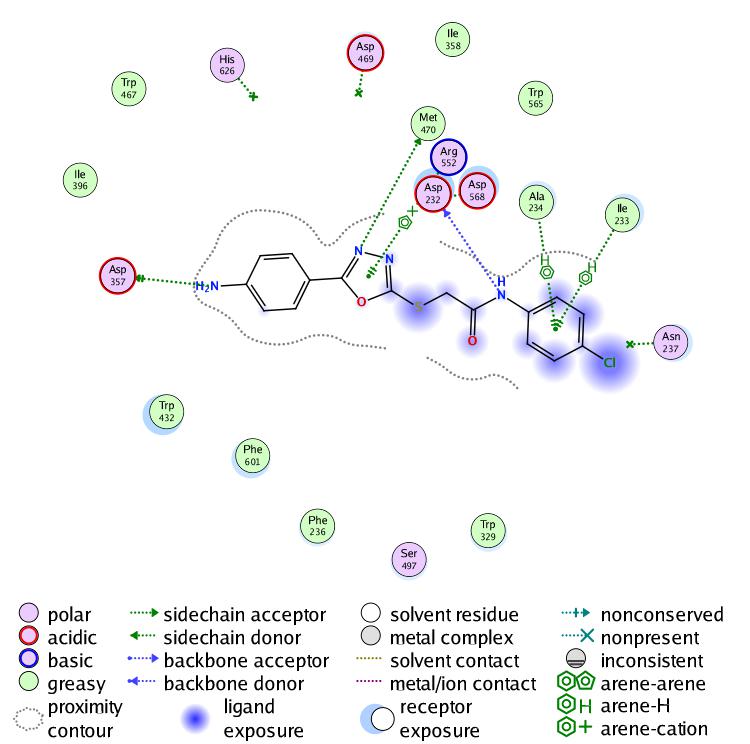 | 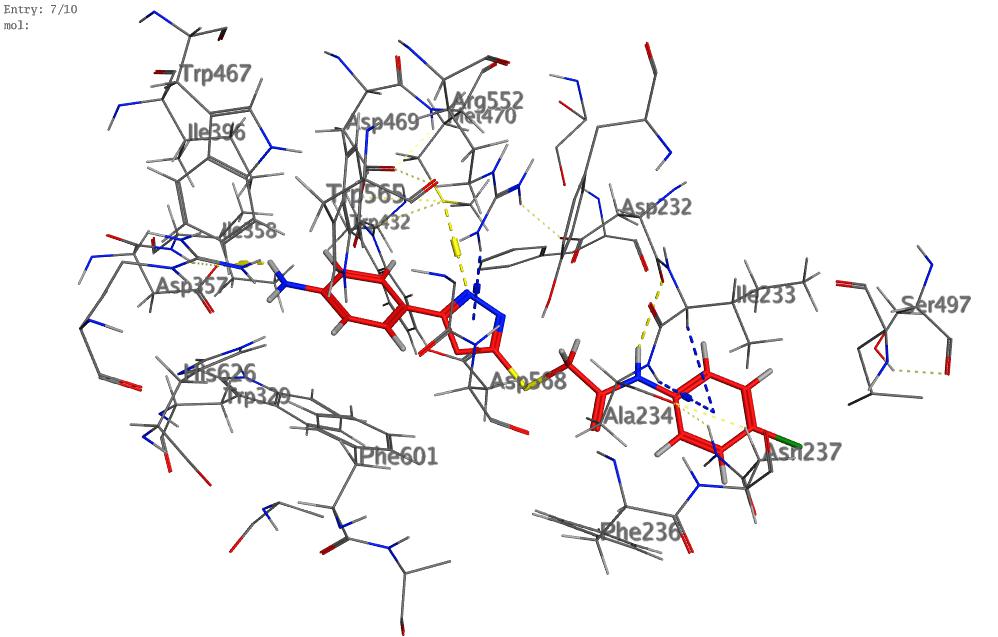 |
| --- | --- |
| **2D** | **3D** |
| 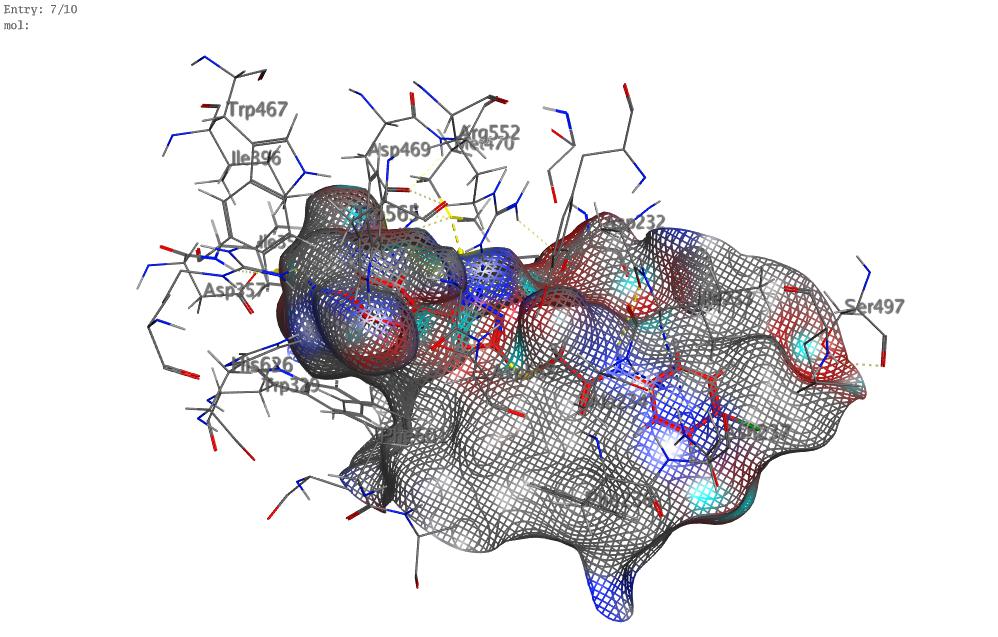 | |
| **Surface map** | |

**Figure S23**: The binding interaction of 1,3,4-oxadiazole **4c** with (PDB ID: 3W37).

| 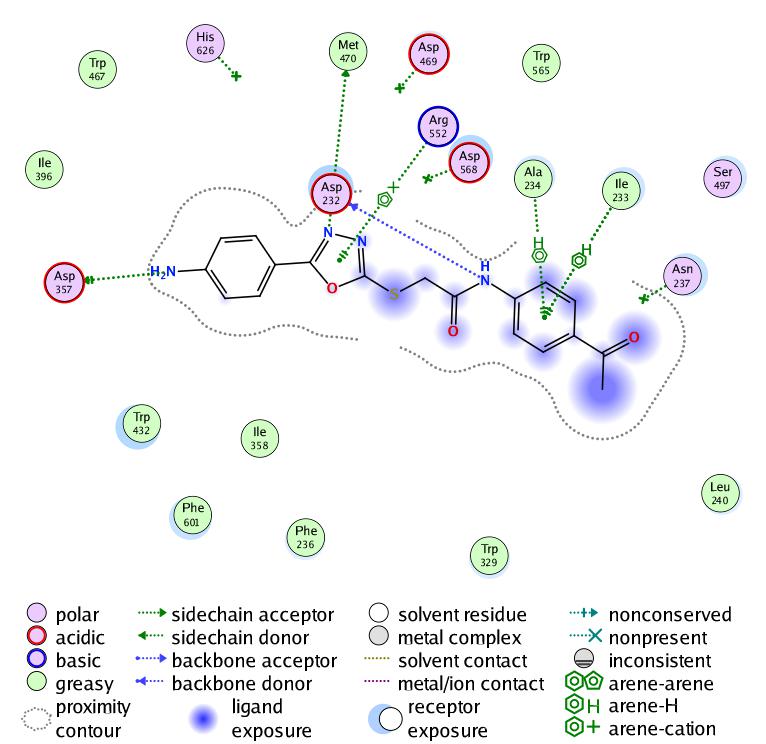 | 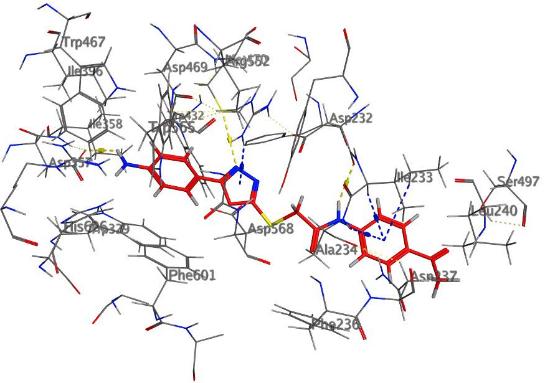 |
| --- | --- |
| **2D** | **3D** |
| 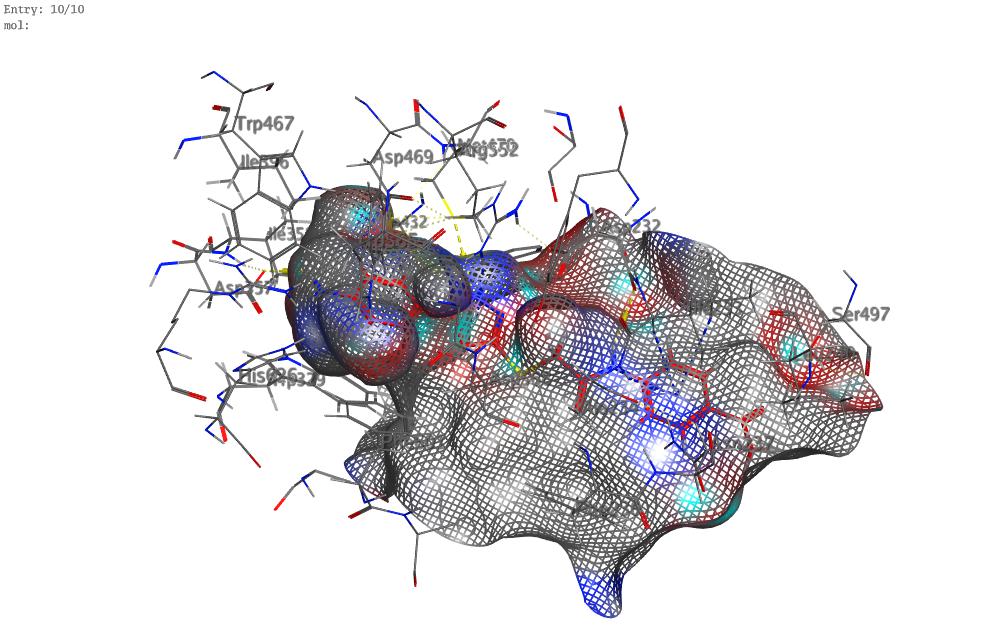 | |
| **Surface map** | |

**Figure S24**: The binding interaction of 1,3,4-oxadiazole **4d** with (PDB ID: 3W37).

| 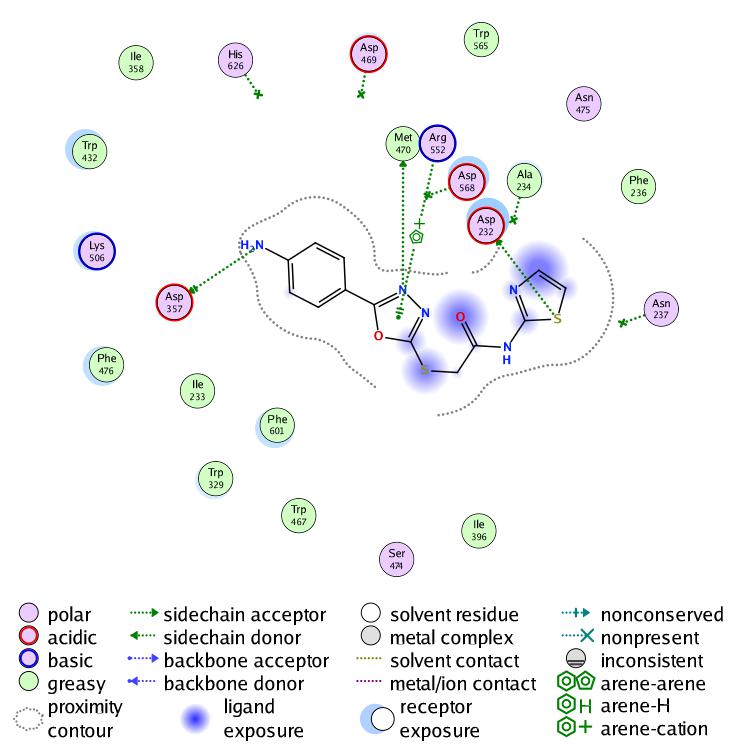 | 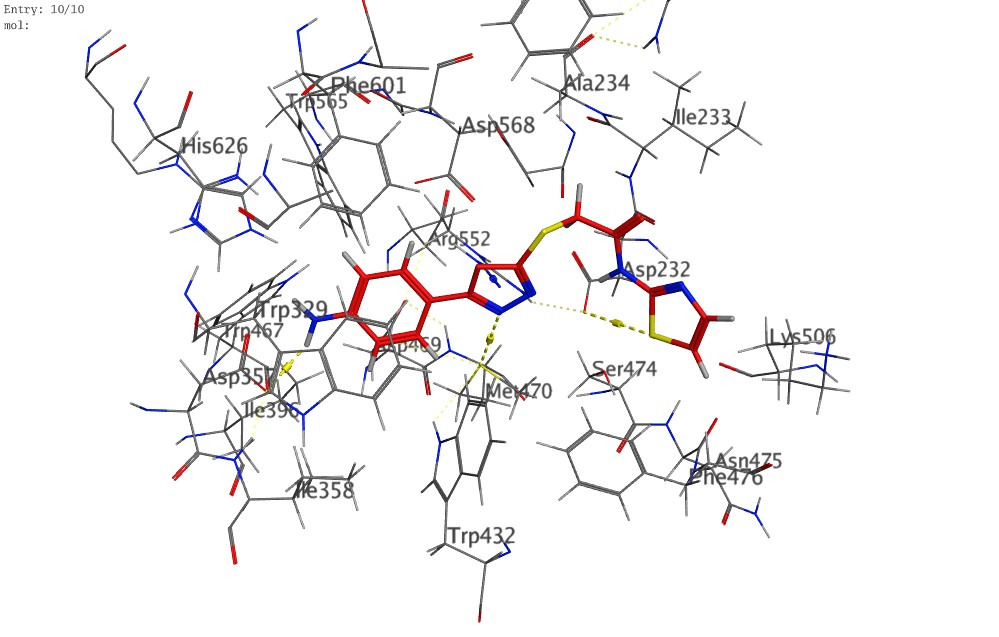 |
| --- | --- |
| **2D** | **3D** |
| 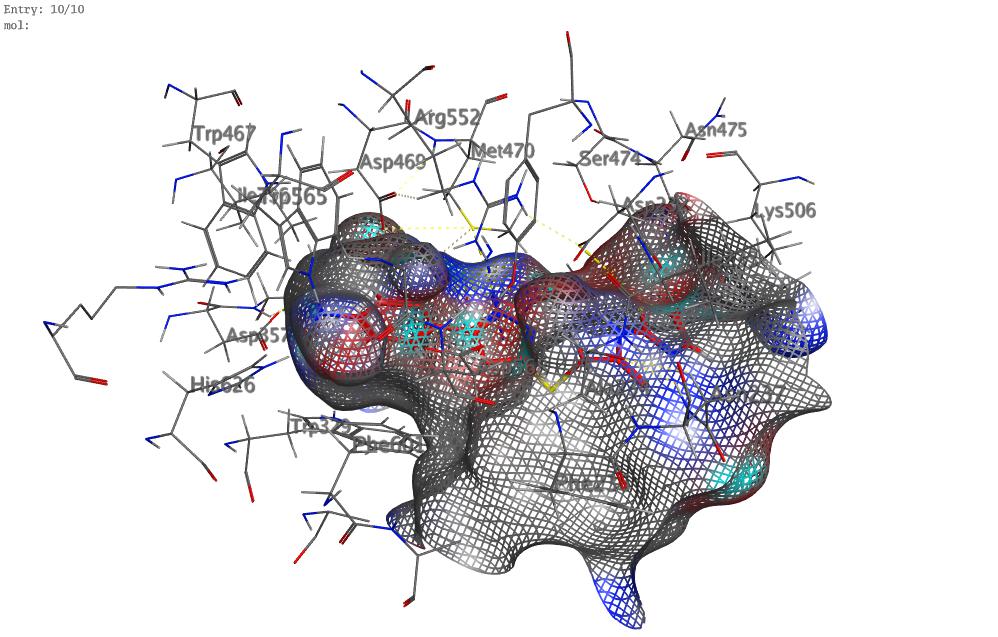 | |
| **Surface map** | |

**Figure S25**: The binding interaction of 1,3,4-oxadiazole **4e** with (PDB ID: 3W37).

| 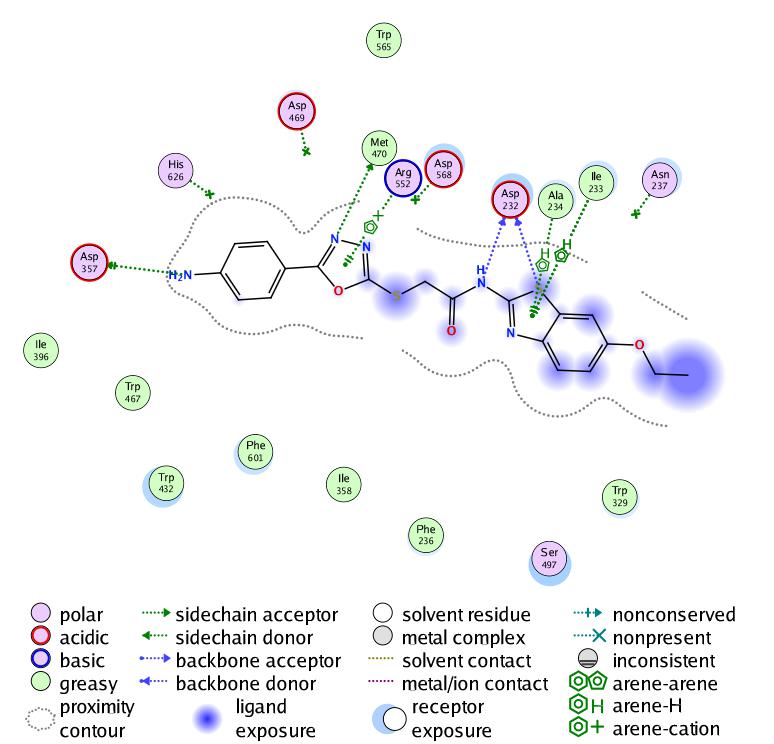 | 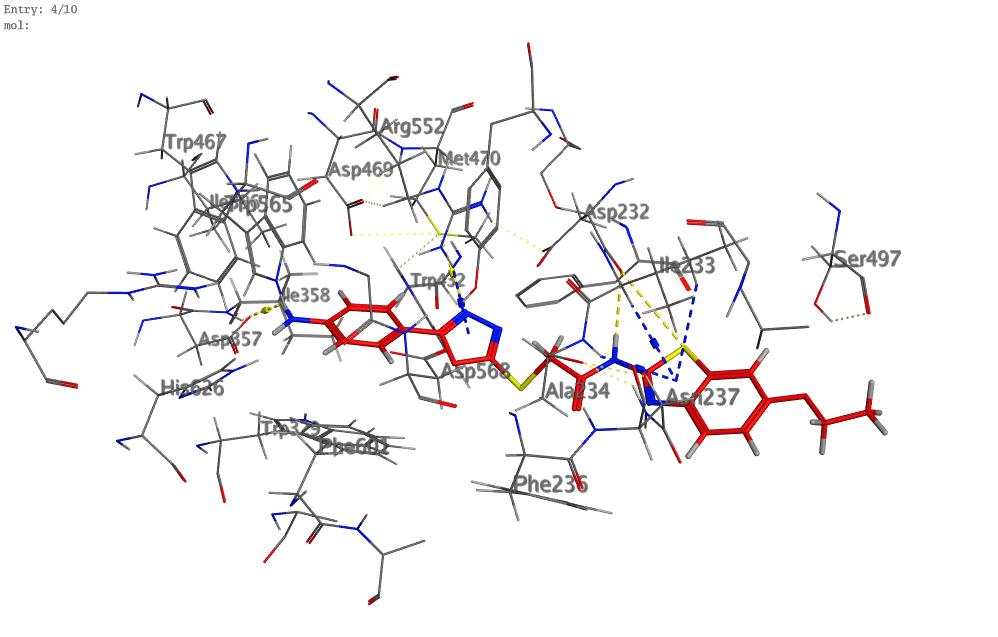 |
| --- | --- |
| **2D** | **3D** |
| 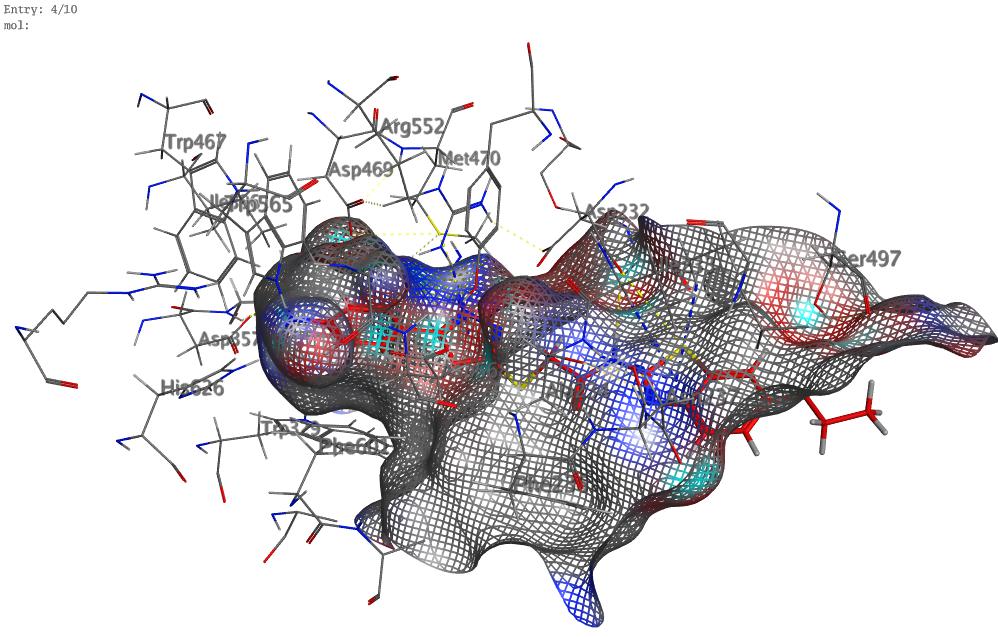 | |
| **Surface map** | |

**Figure S26**: The binding interaction of 1,3,4-oxadiazole **4f** with (PDB ID: 3W37).

| 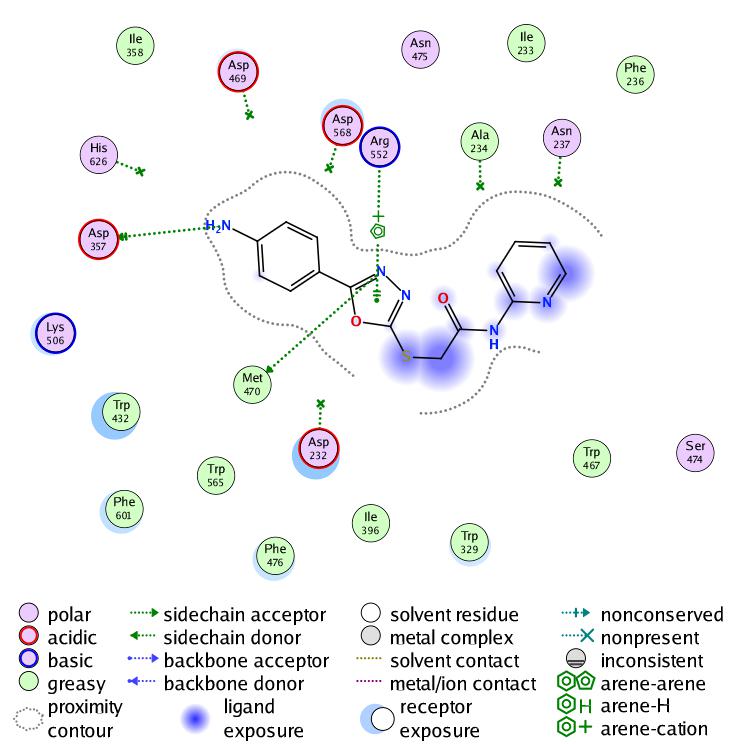 | 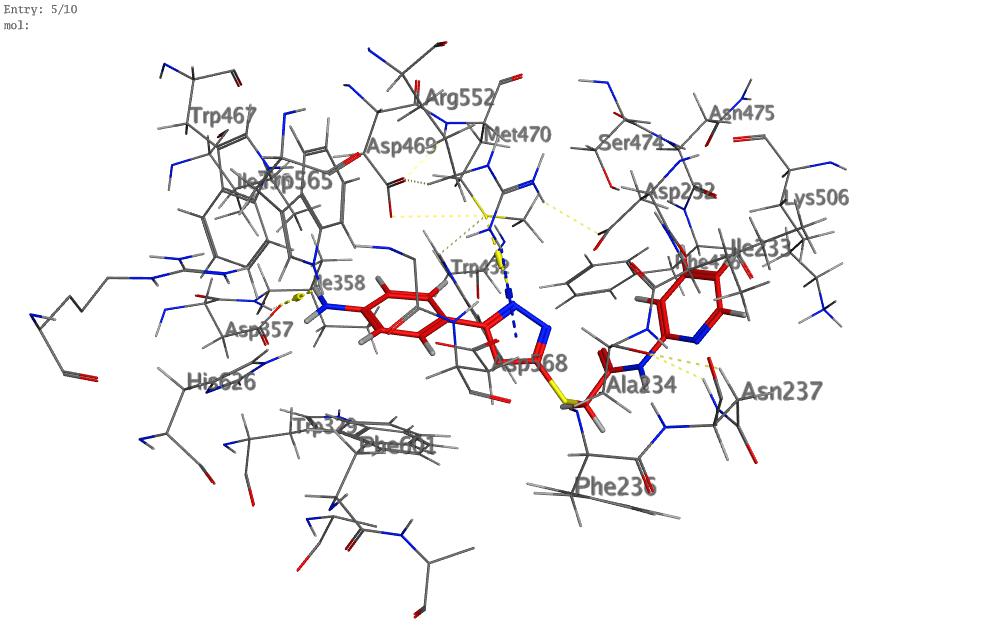 |
| --- | --- |
| **2D** | **3D** |
| 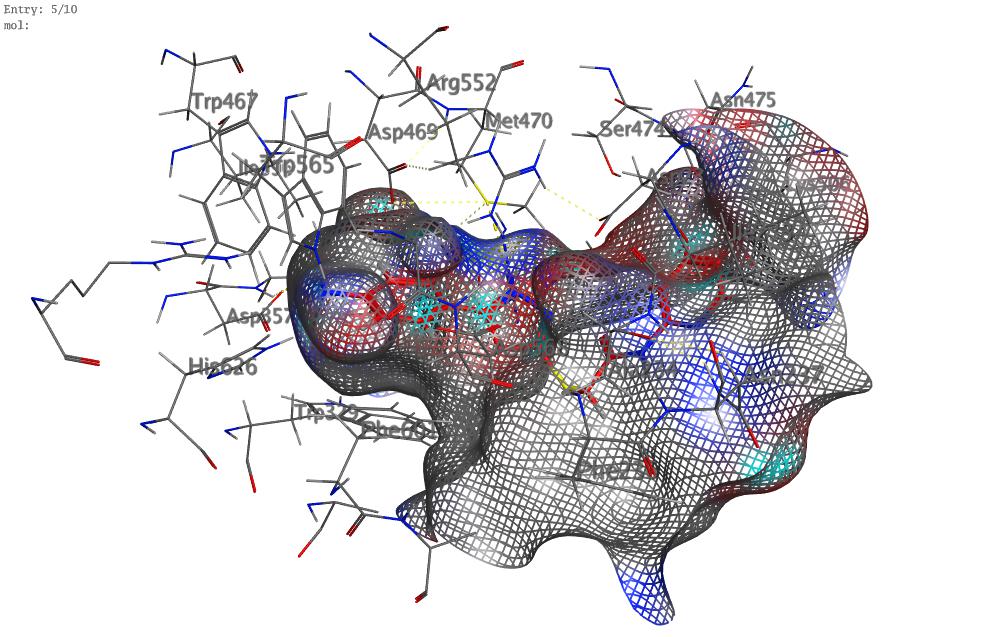 | |
| **Surface map** | |

**Figure S27**: The binding interaction of 1,3,4-oxadiazole **4g** with (PDB ID: 3W37).

| 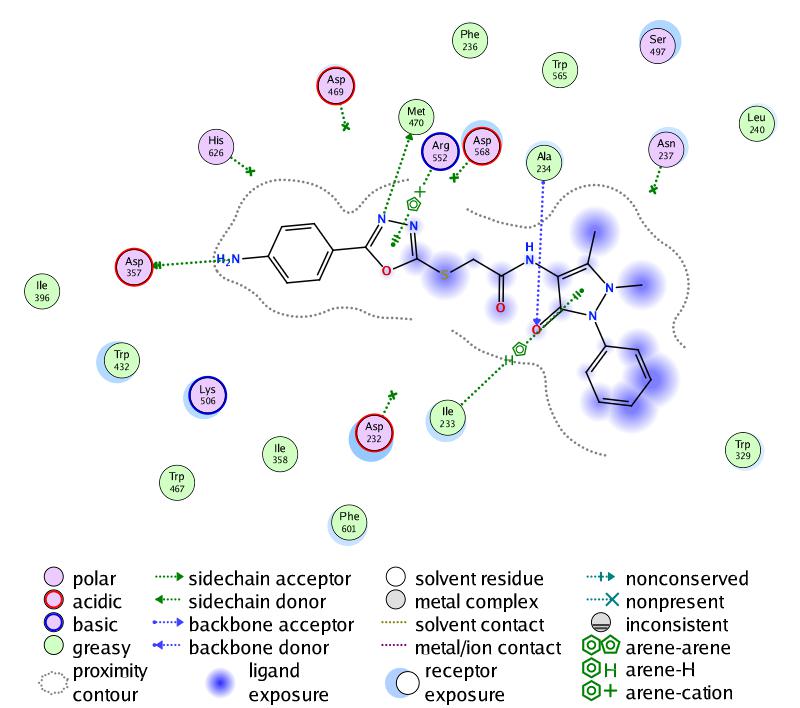 | 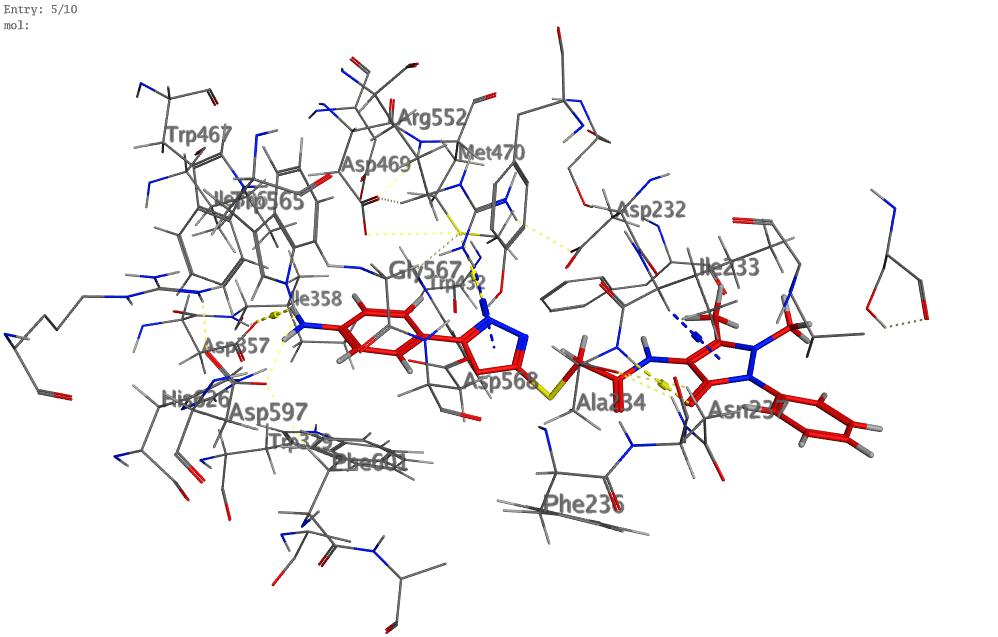 |
| --- | --- |
| **2D** | **3D** |
| 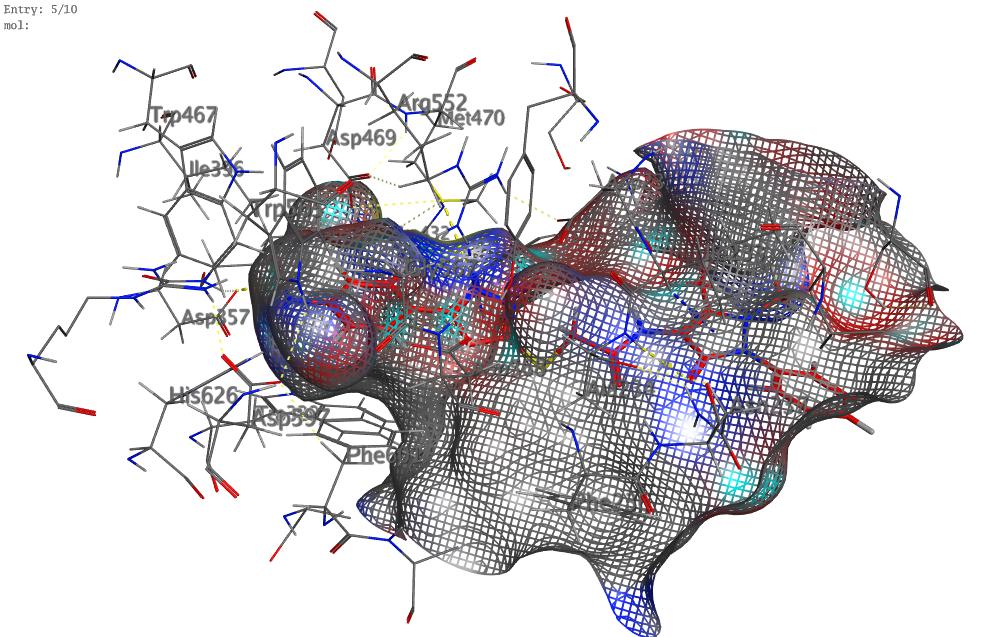 | |
| **Surface map** | |

**Figure S28**: The binding interaction of 1,3,4-oxadiazole **4h** with (PDB ID: 3W37).

**DFT-calculations**

| 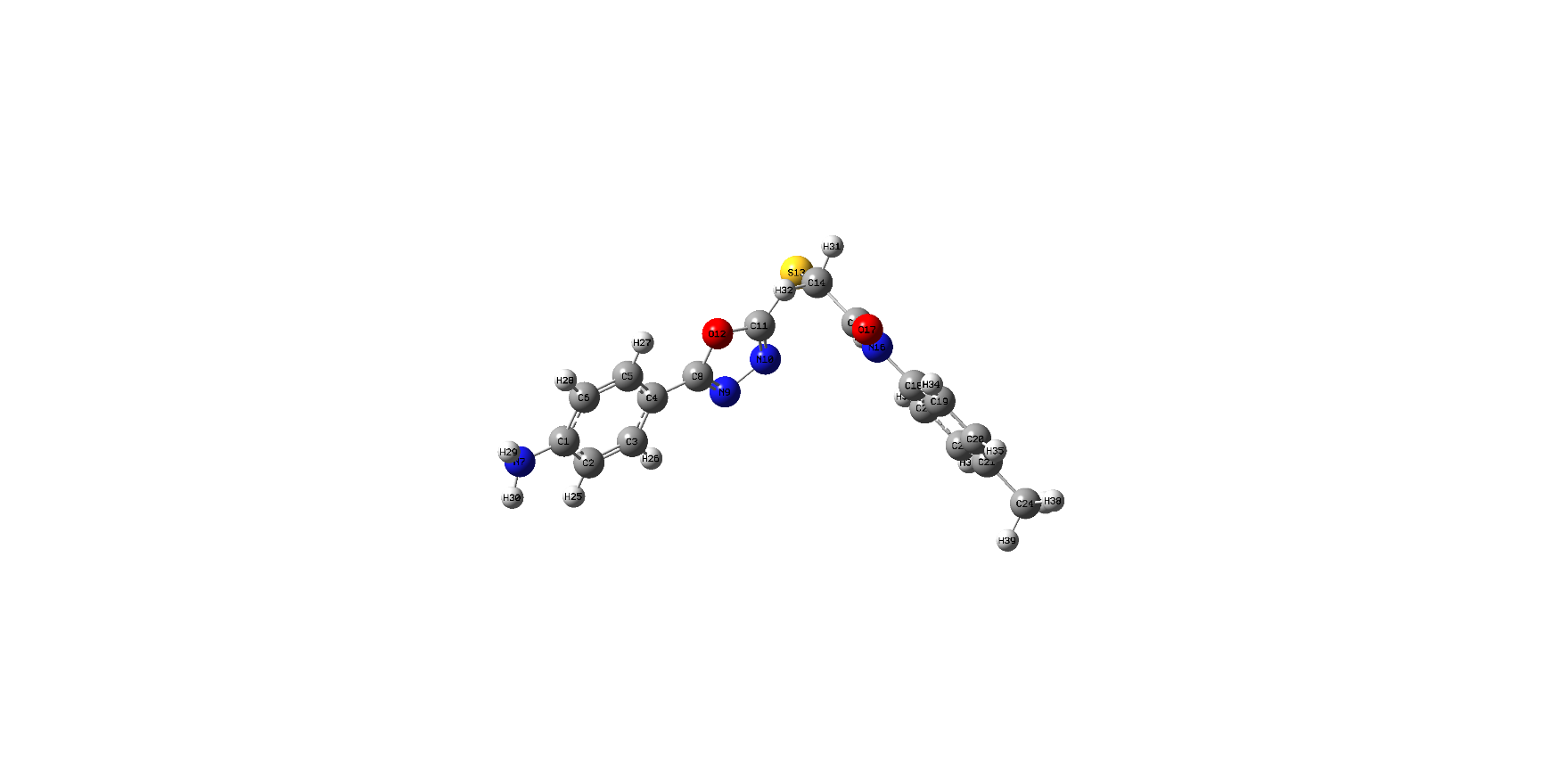  **Optimized Structure** | **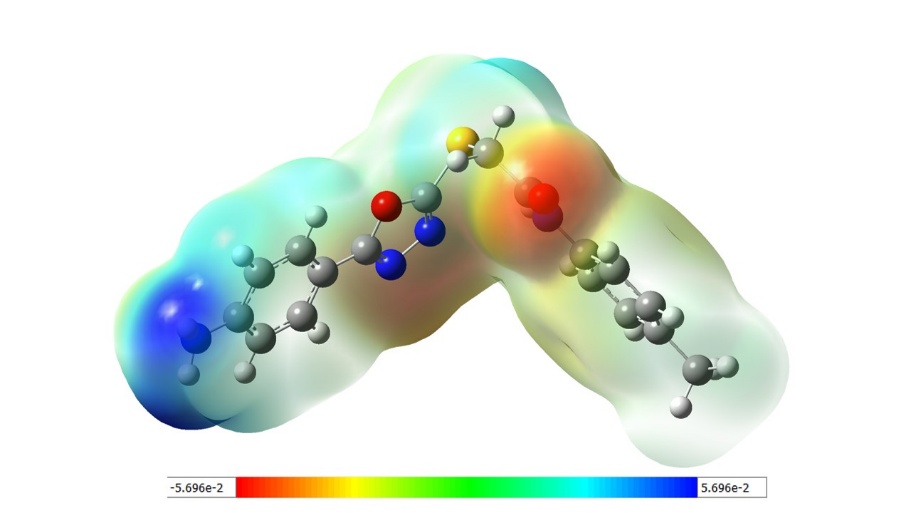**  **MEP** |
| --- | --- |
| 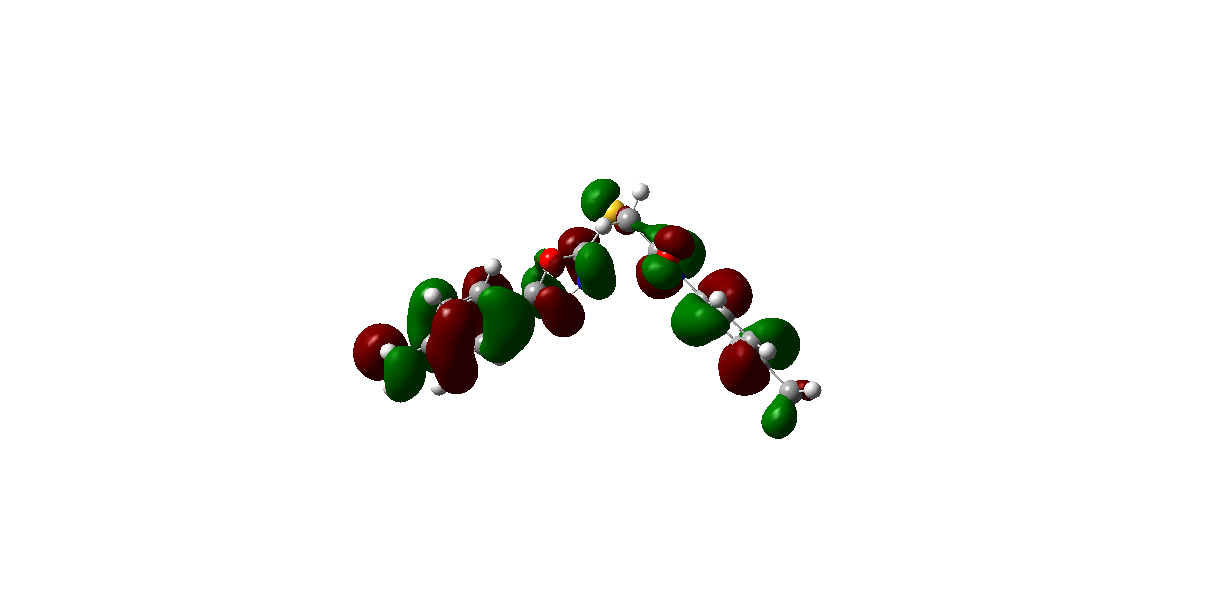 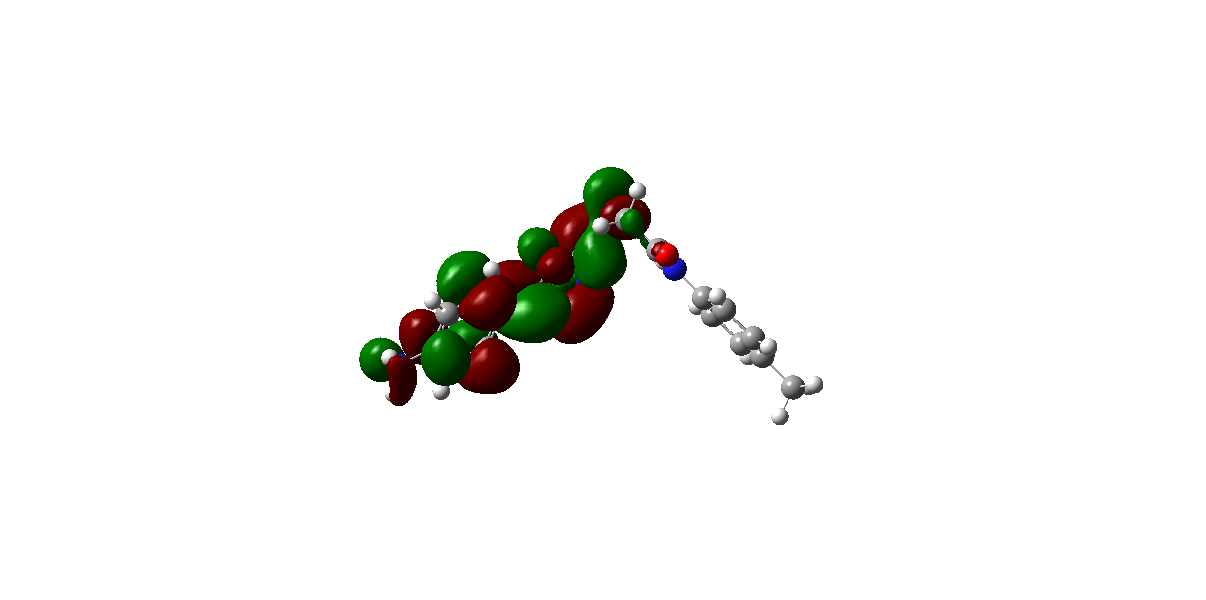  **E_gap_= 4.38 eV**  **HOMO** (-6.09 eV) **LUMO** (-1.71 eV) | |
| **Fig. S29.** Optimized structures, electron density and HOMO & LUMO for **4a** compound. | |

| 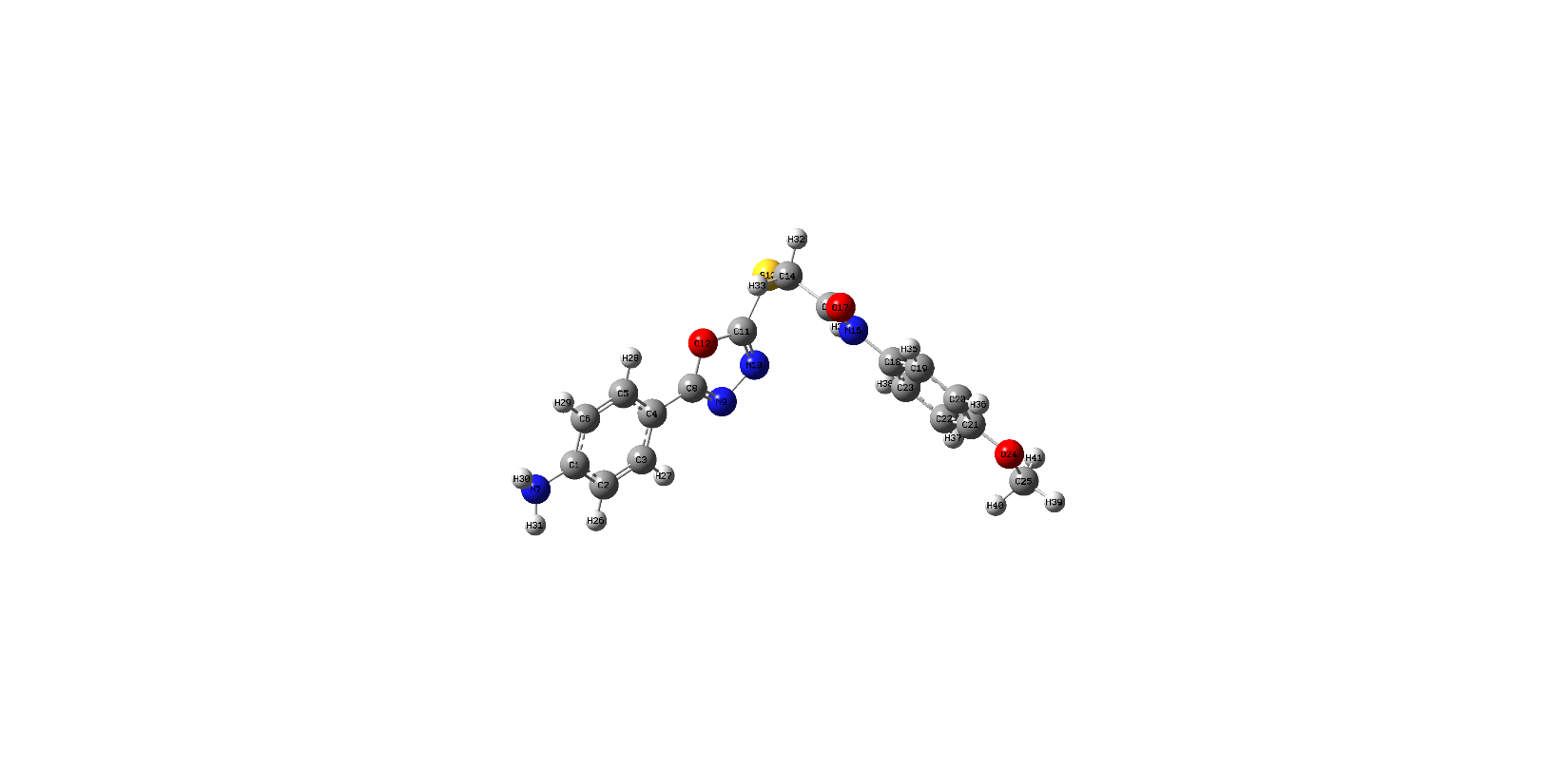  **Optimized Structure** | **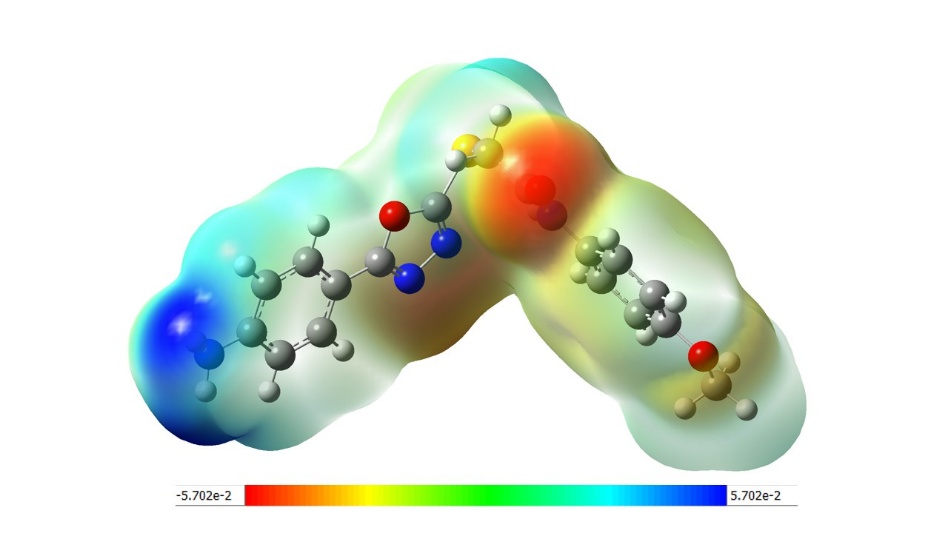**  **MEP** |
| --- | --- |
| 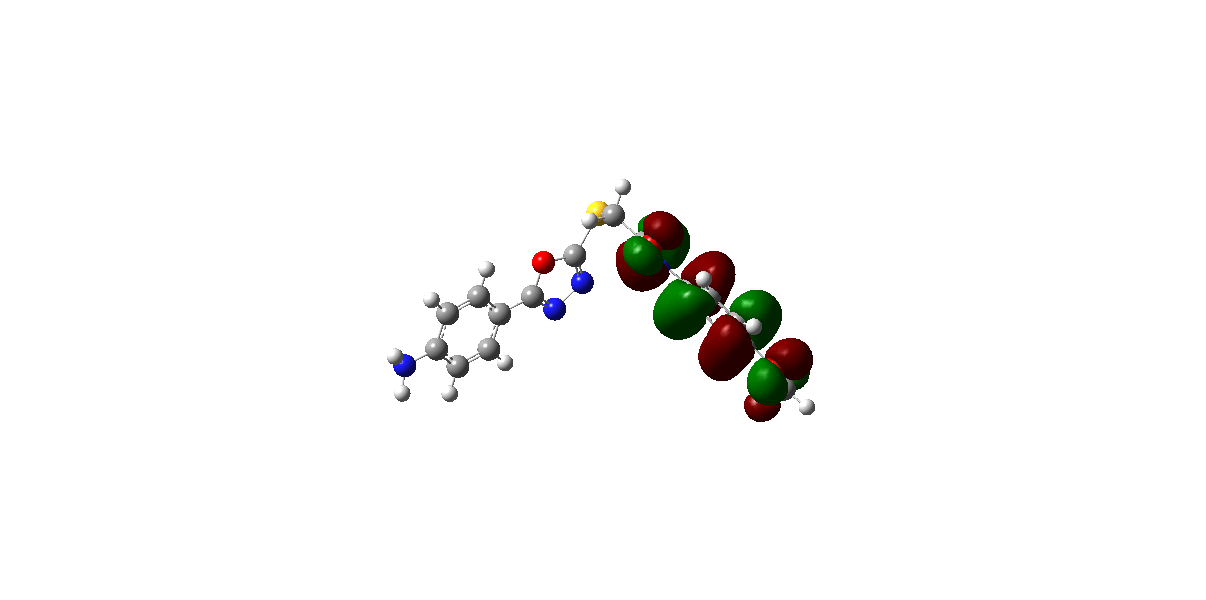 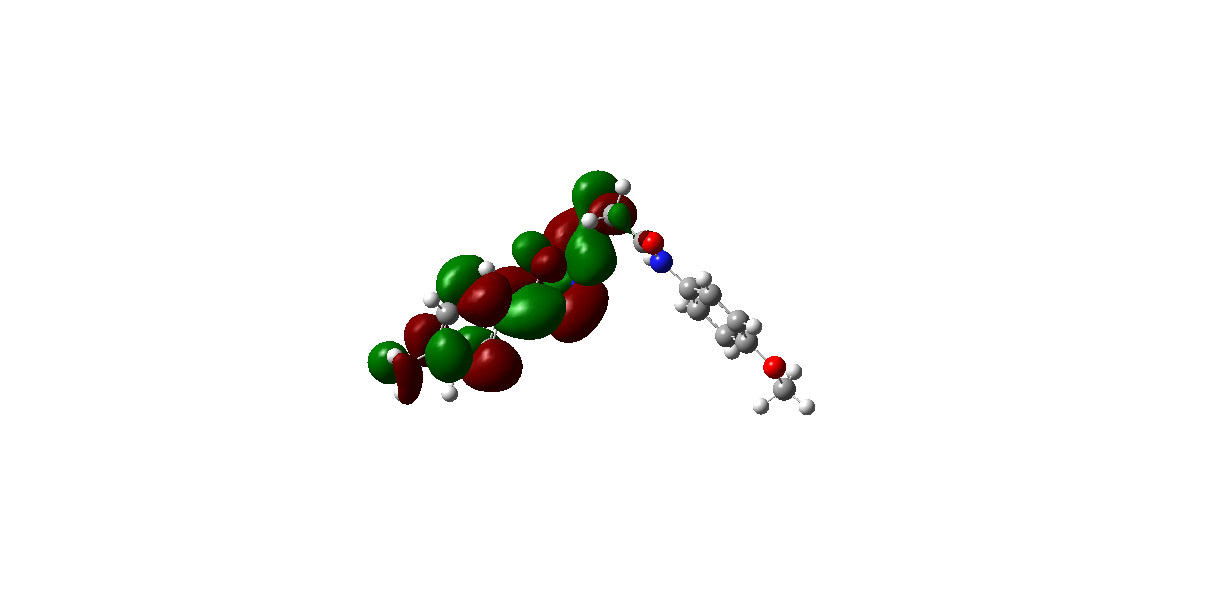  **E_gap_= 4.07 eV**  **HOMO** (- 5.78eV) **LUMO** (-1.71 eV) | |
| **Fig.S30.** Optimized structures, electron density and HOMO & LUMO for **4b** compound. | |

| 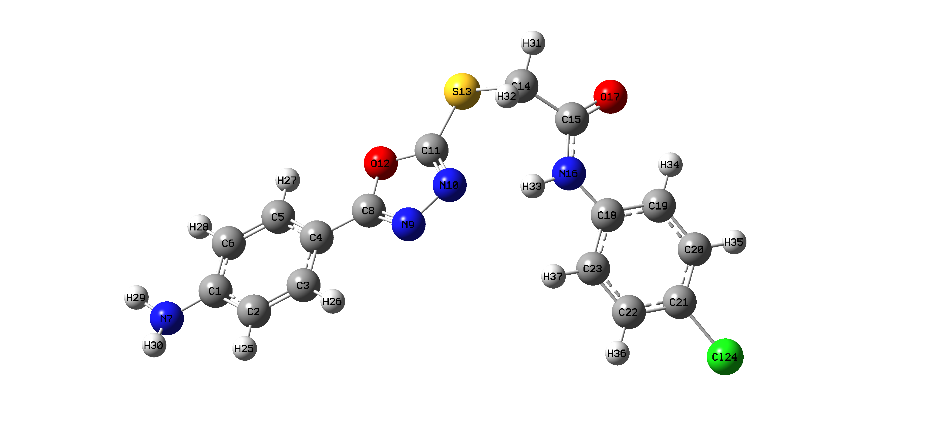  **Optimized Structure** | 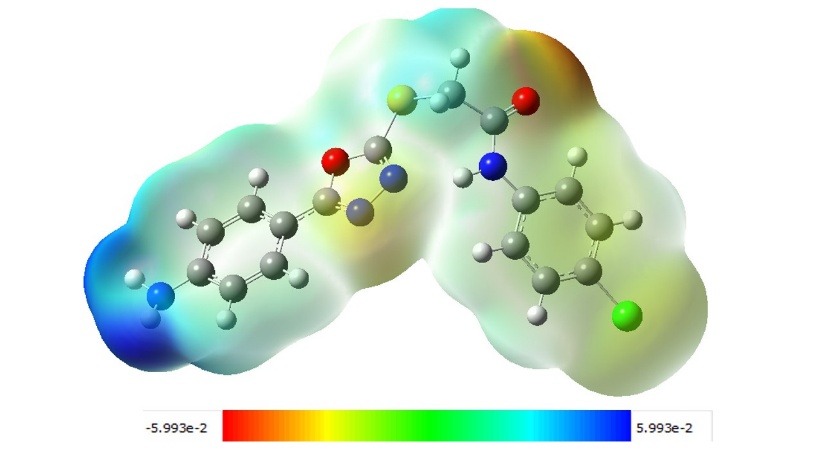  **MEP** |
| --- | --- |
| 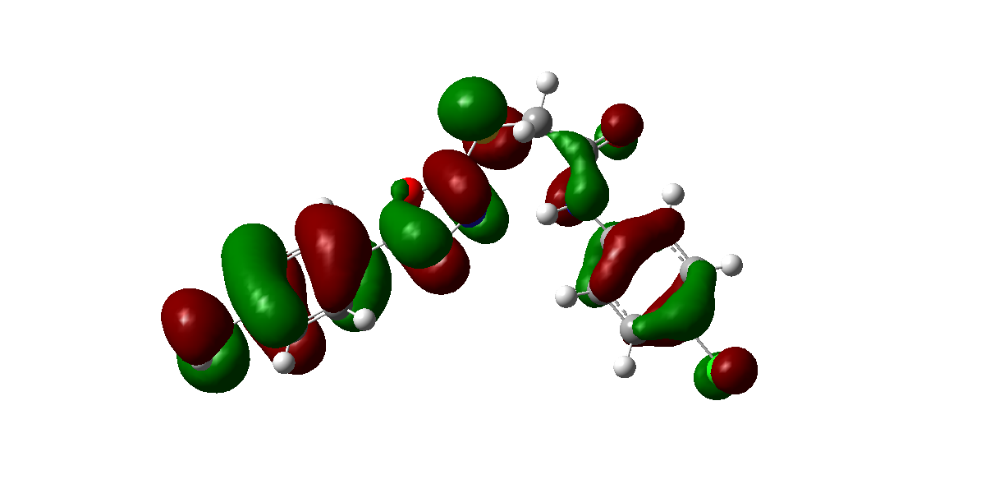 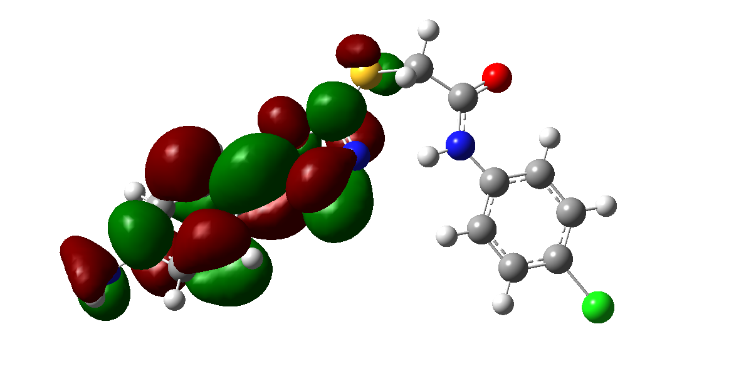  **E_gap_= 4.45 eV**  **HOMO** (-6.16 eV )  **LUMO** (-1.71 eV) | |
| **Fig. S31.** Optimized structures, electron density and HOMO & LUMO for **4c** compound. | |

| 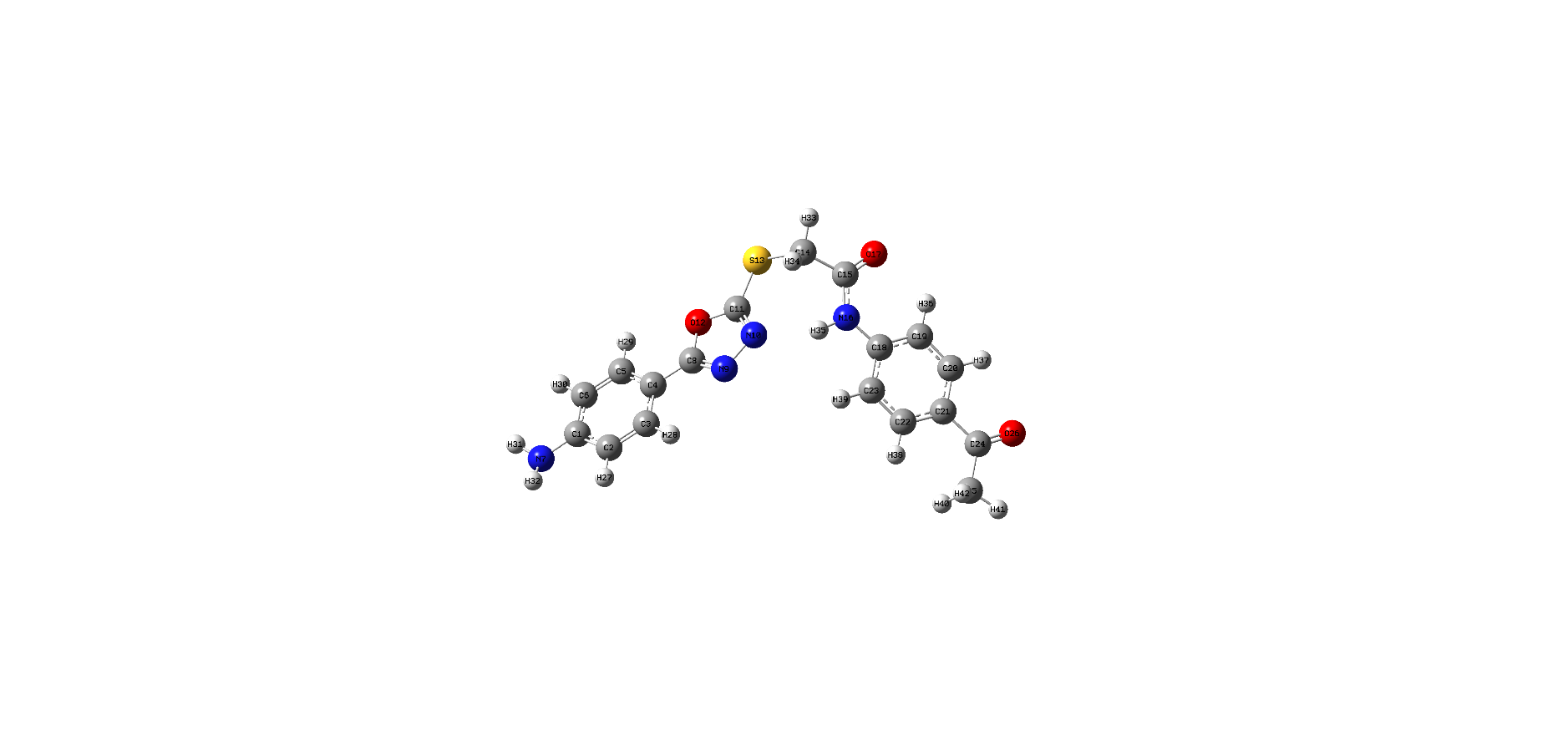  **Optimized Structure** | 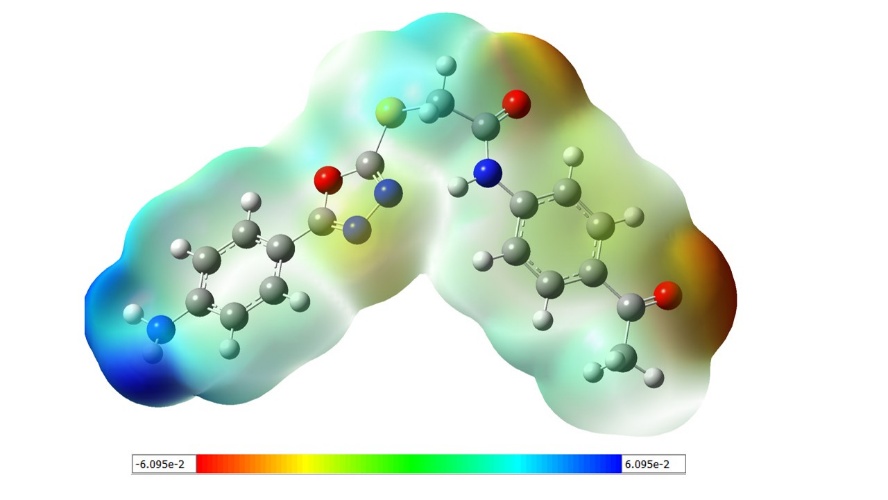  **MEP** |
| --- | --- |
| 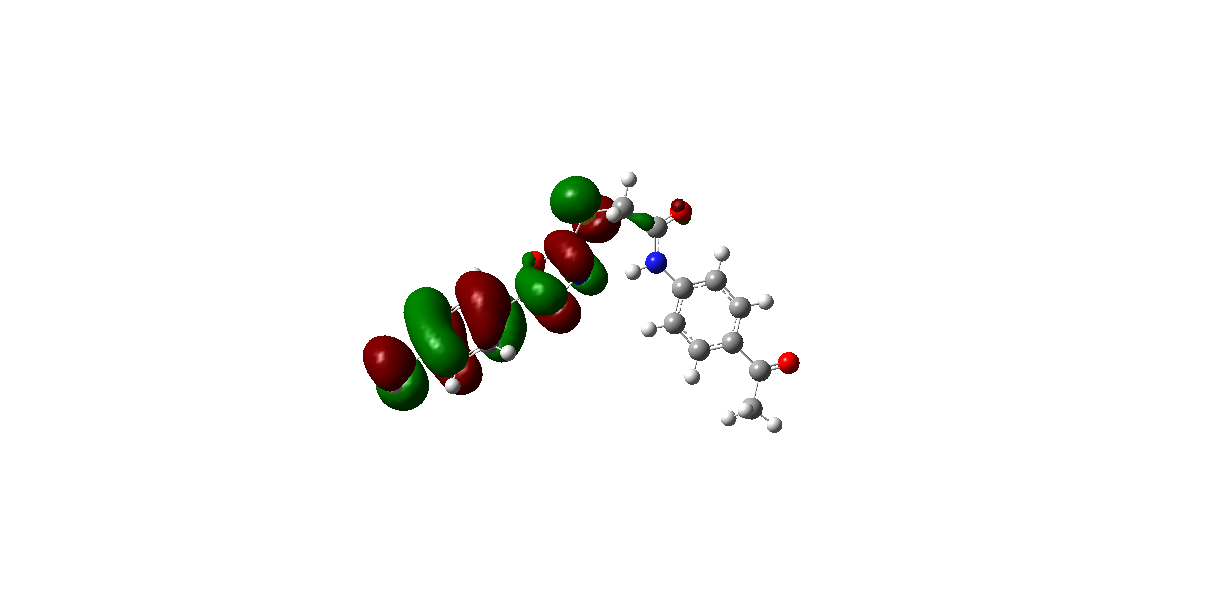 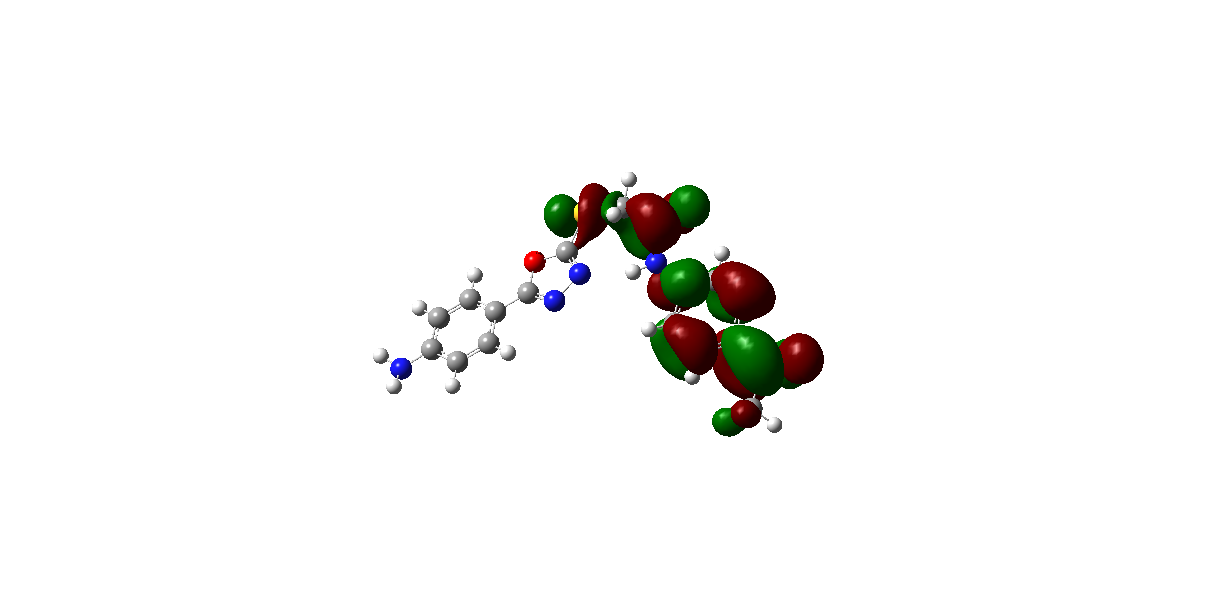  **E_gap_= 4.42 eV**  **HOMO** (-6.21 eV) **LUMO** (-1.78 eV) | |
| **Fig. S32.** Optimized structures, electron density and HOMO & LUMO for **4d** compound. | |

| 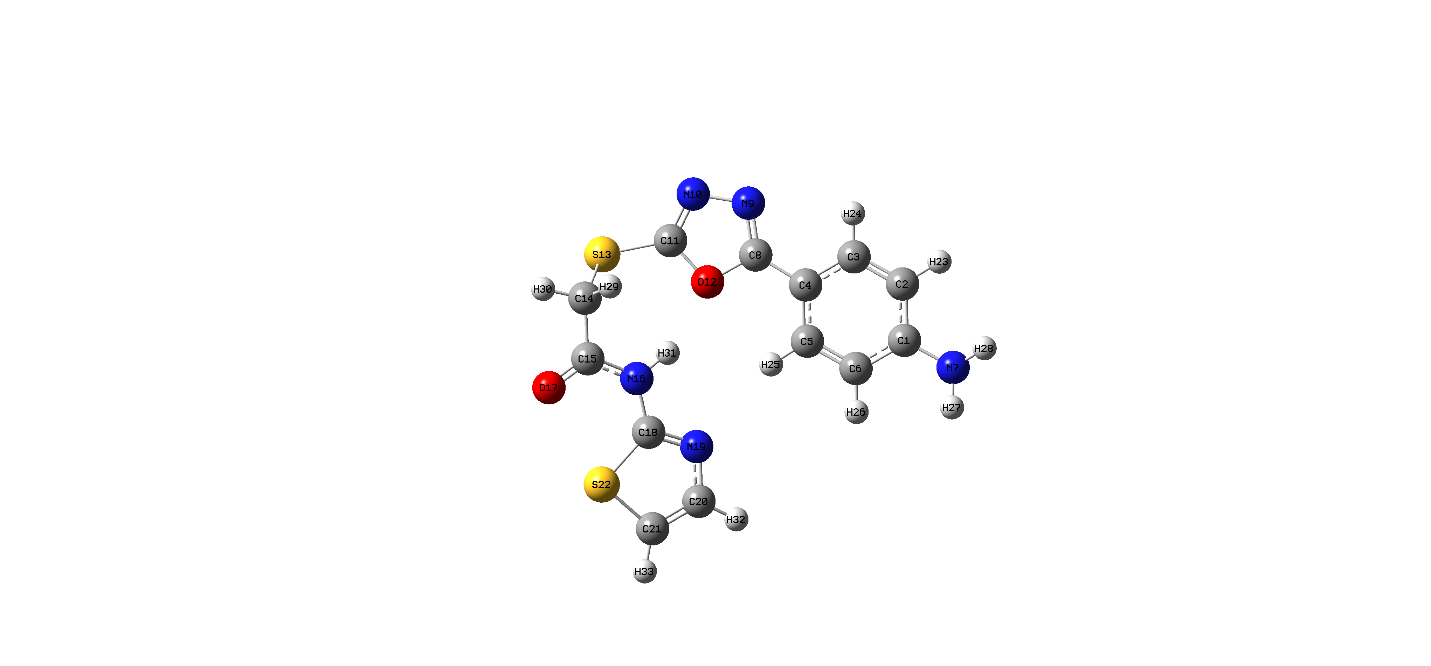  **Optimized Structure** | 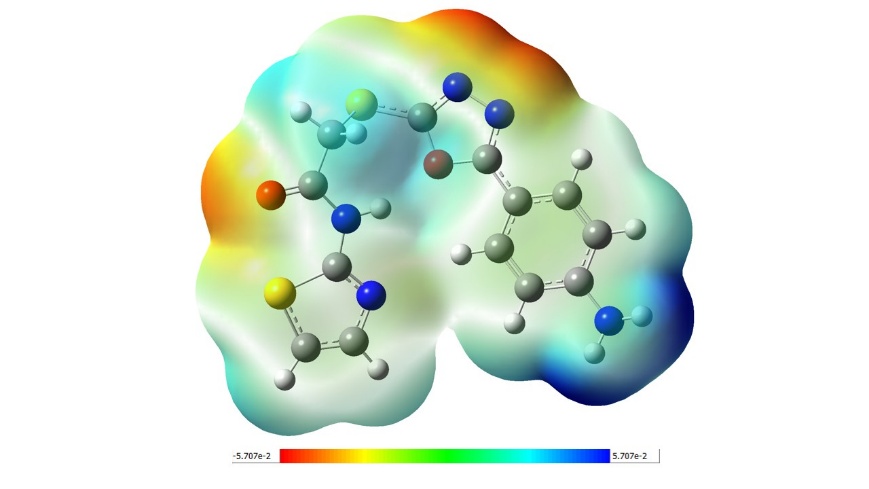  **MEP** |
| --- | --- |
| 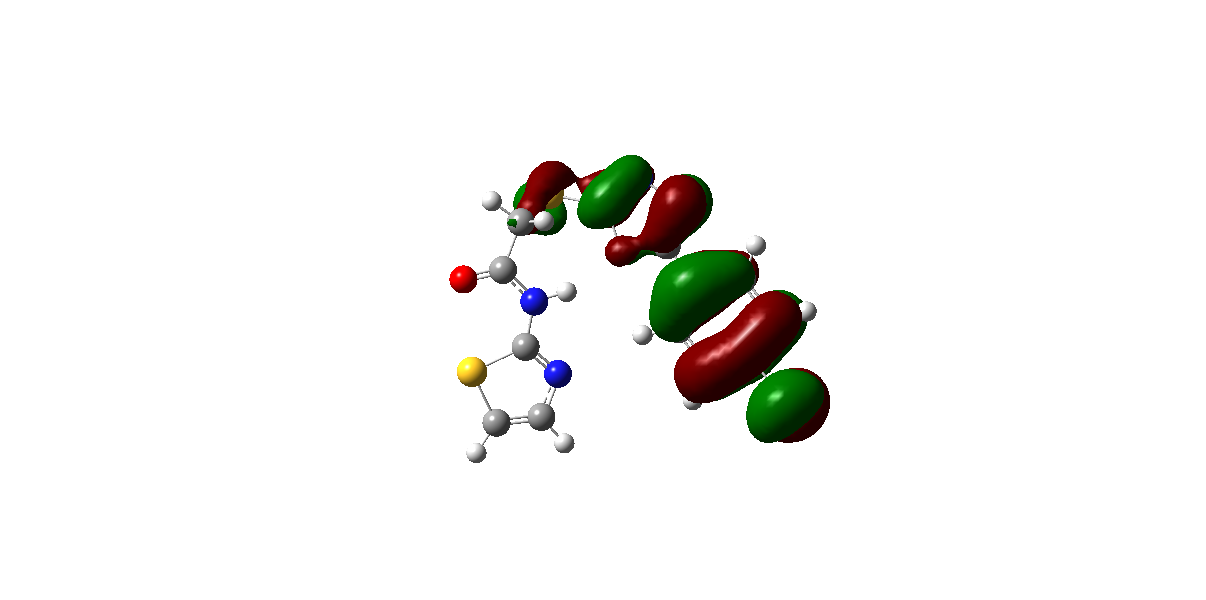 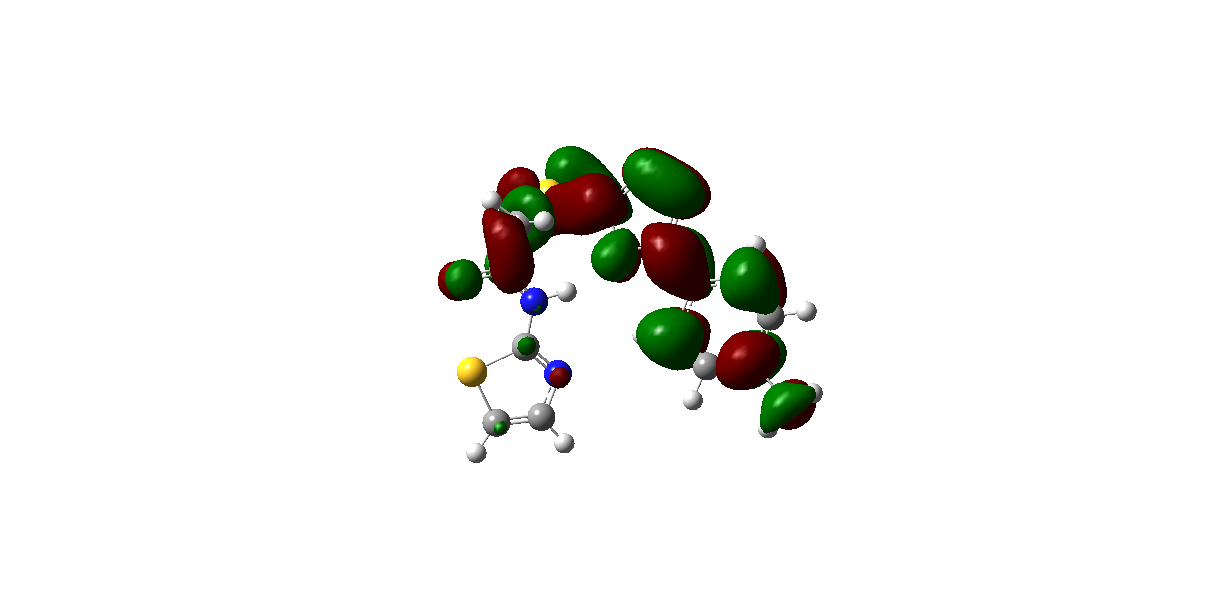  **E_gap_= 4.30 eV**  **HOMO** (-6.14 eV) **LUMO** (-1.84 eV) | |
| **Fig. S33.** Optimized structures, electron density, and HOMO & LUMO for **4e** compound. | |

| 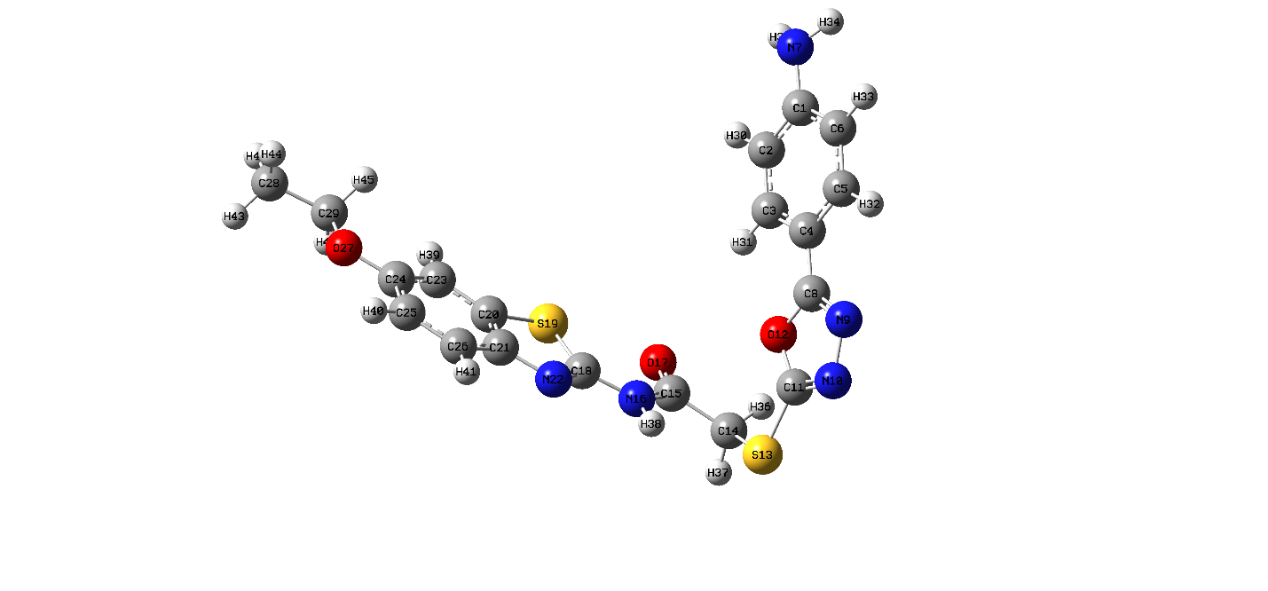  **Optimized Structure** | 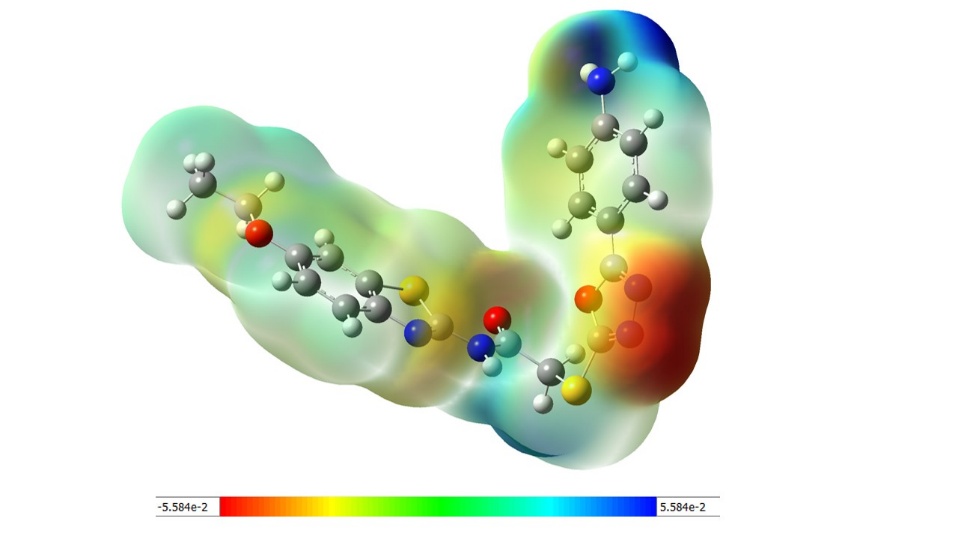  **MEP** |
| --- | --- |
| 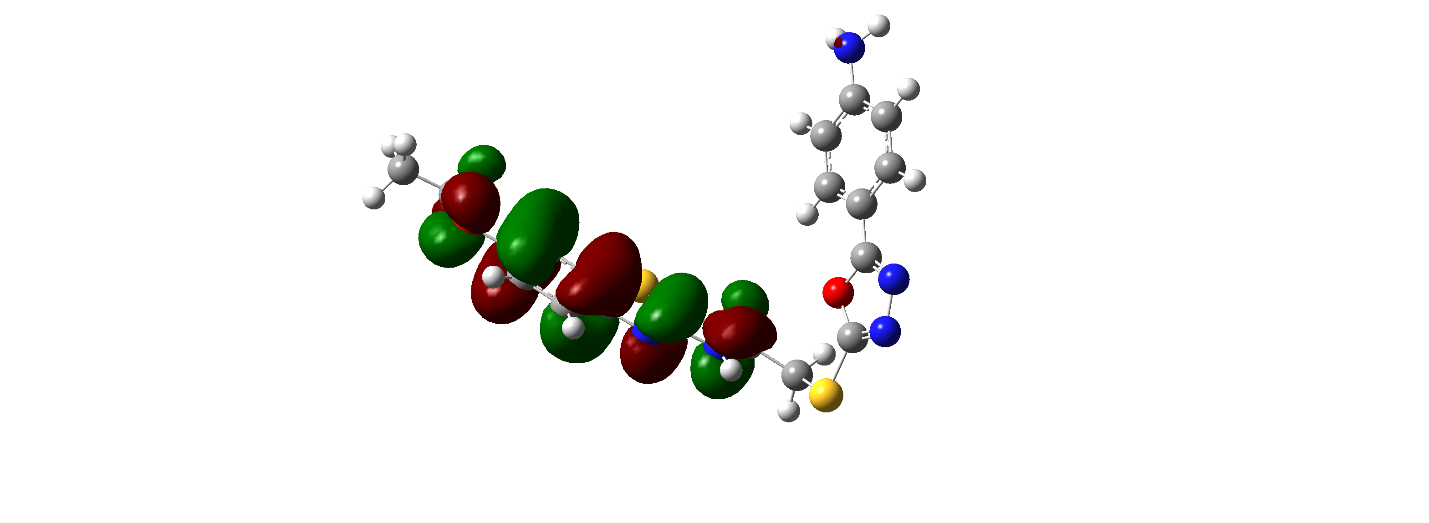 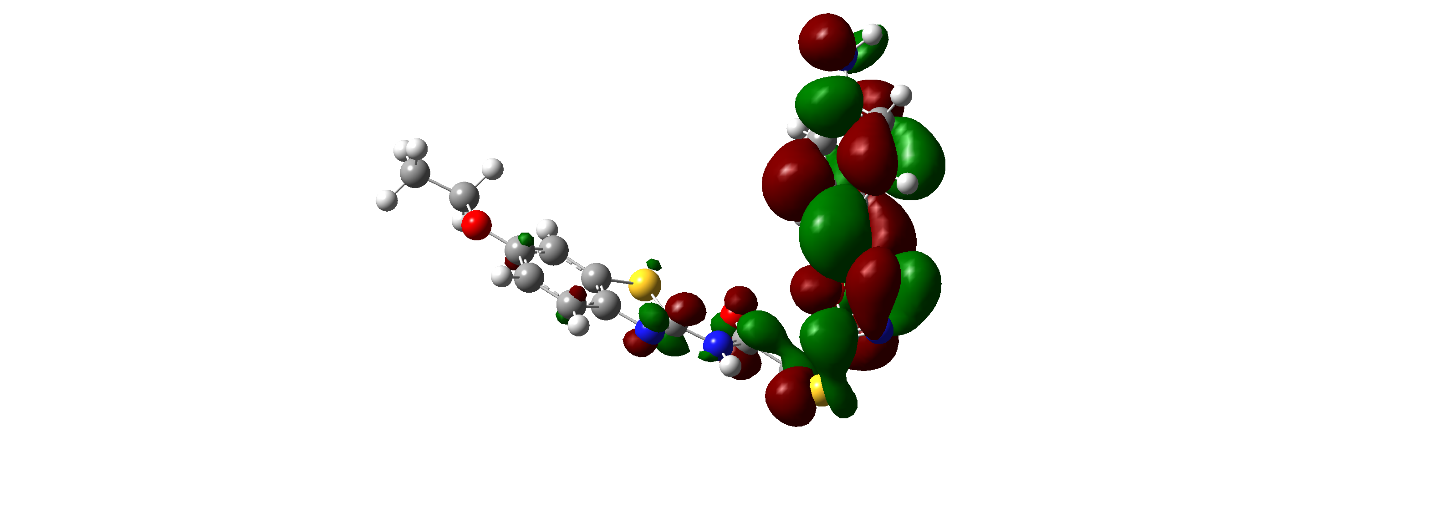  **E_gap_= 4.46 eV**  **HOMO** (-5.90 eV) **LUMO** (-1.44 eV) | |
| **Fig. S34.** Optimized structures, electron density, and HOMO & LUMO for **4f** compound. | |

| 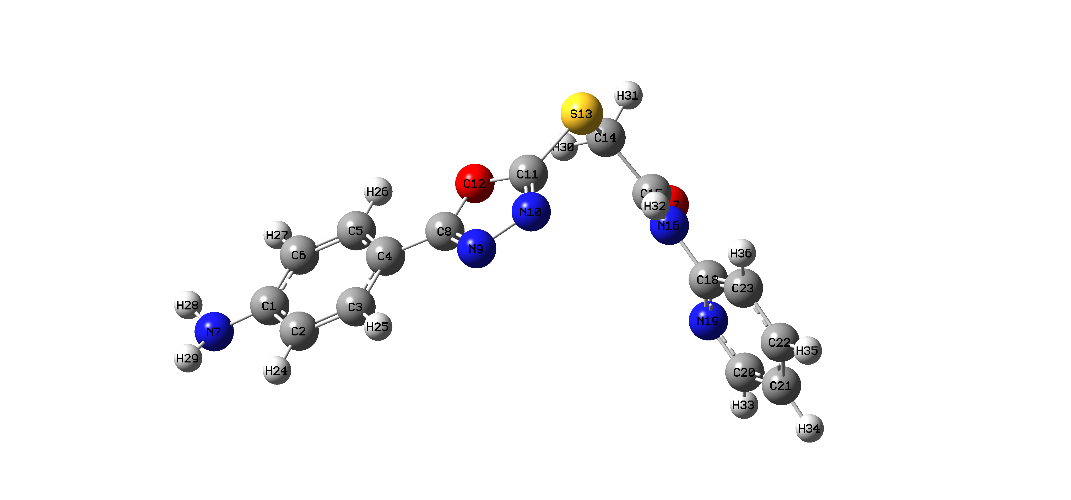  **Optimized Structure** | 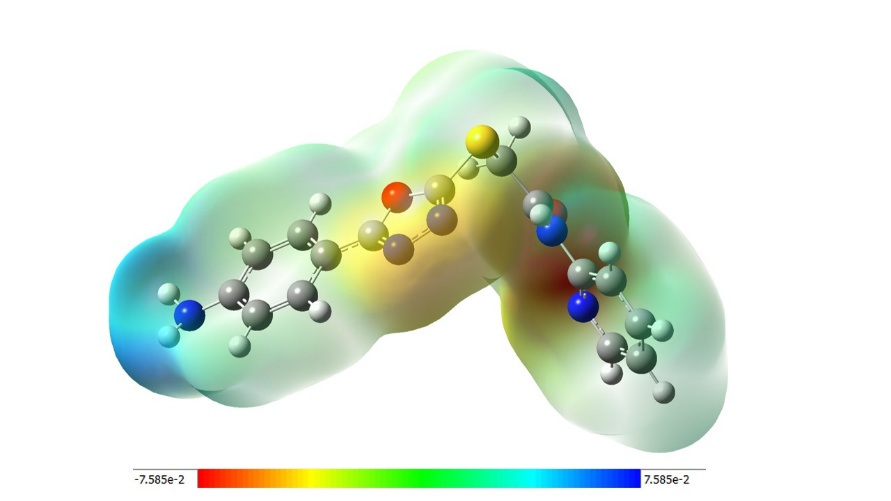  **MEP** |
| --- | --- |
| 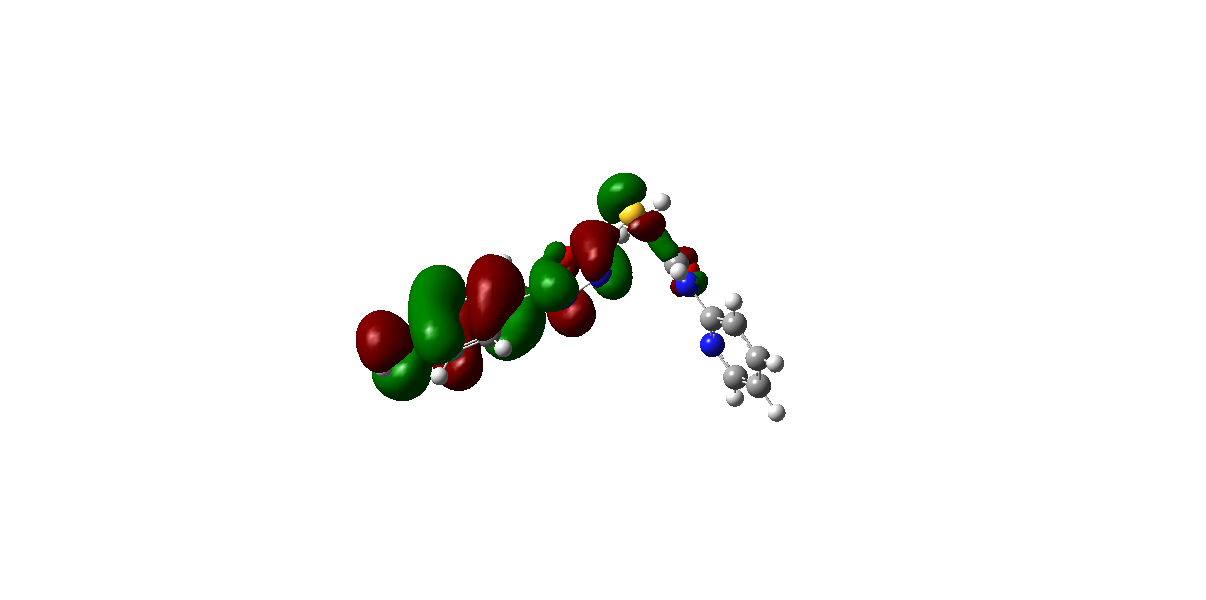 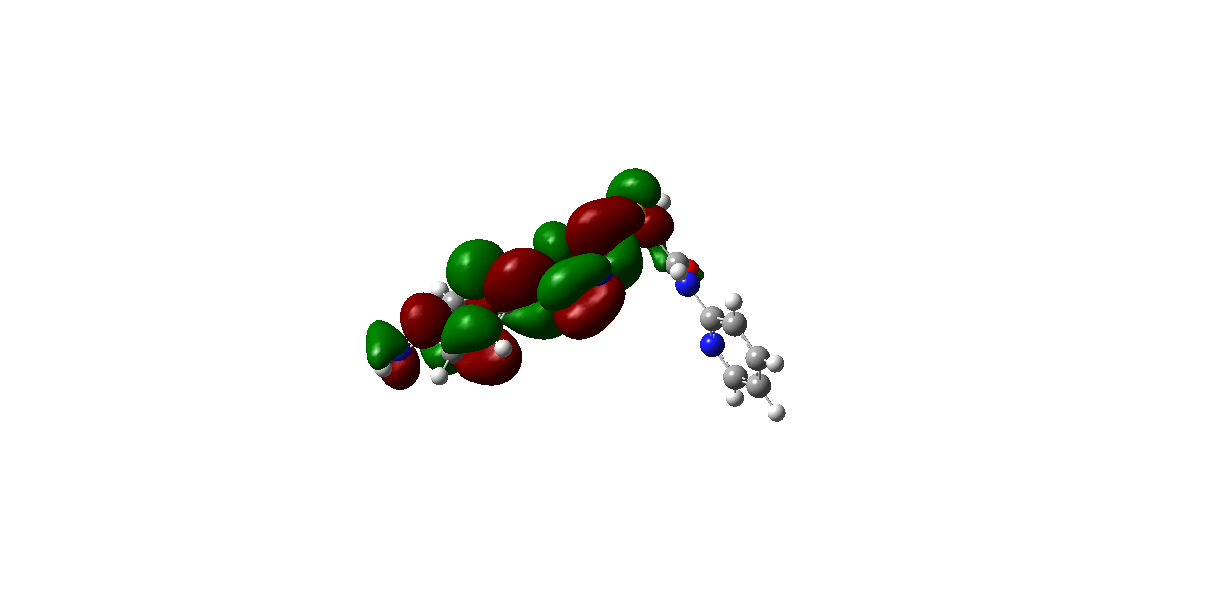  **E_gap_= 4.41 eV**  **HOMO** (-6.08 eV) **LUMO** (-1.68 eV) | |
| **Fig. S35.** Optimized structures, electron density, and HOMO & LUMO for **4g** compound. | |

| 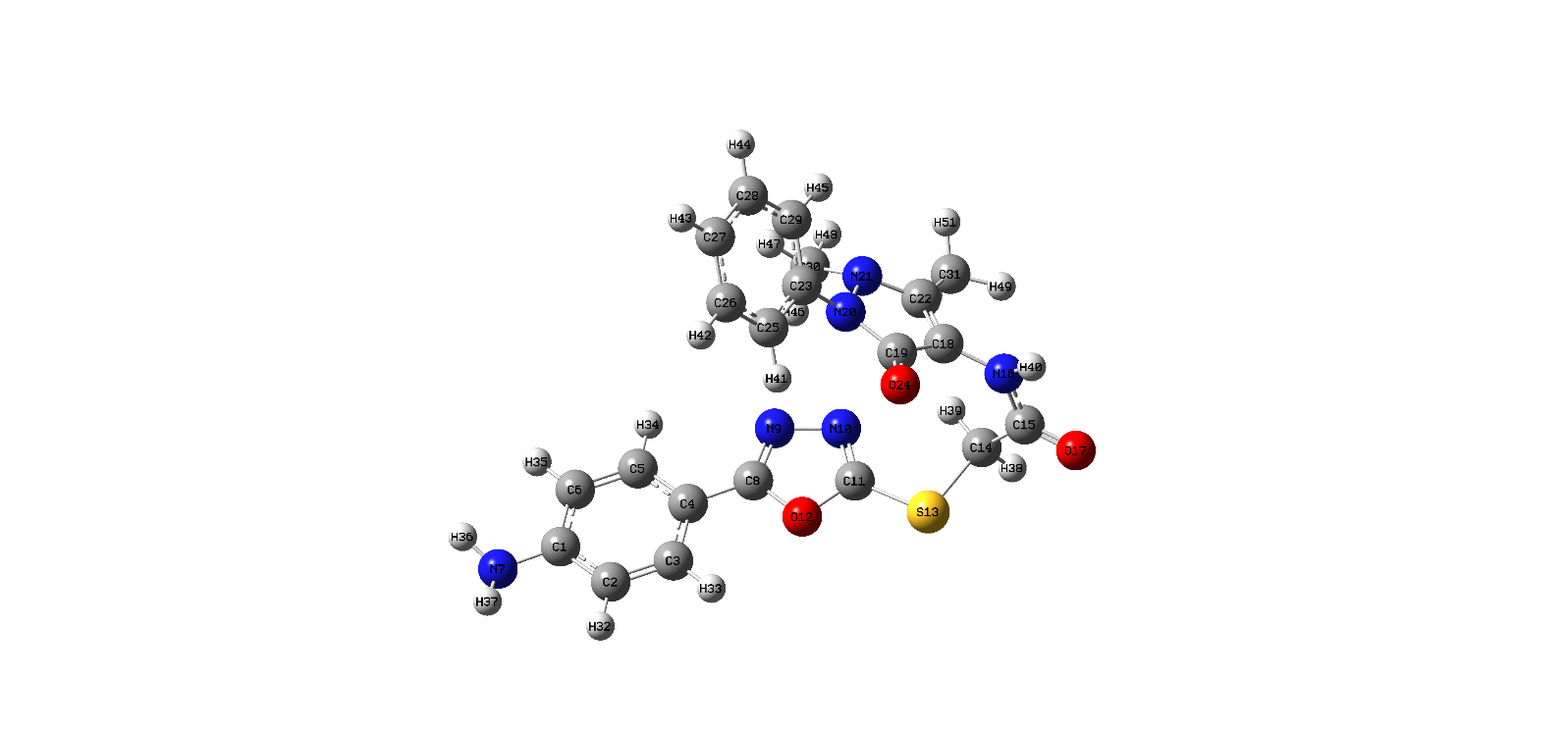  **Optimized Structure** | 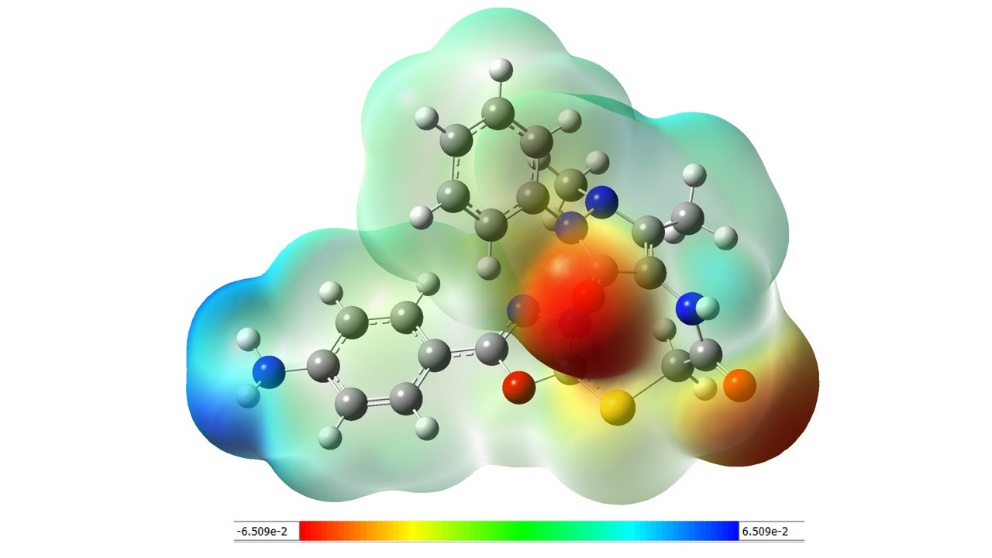  **MEP** |
| --- | --- |
| 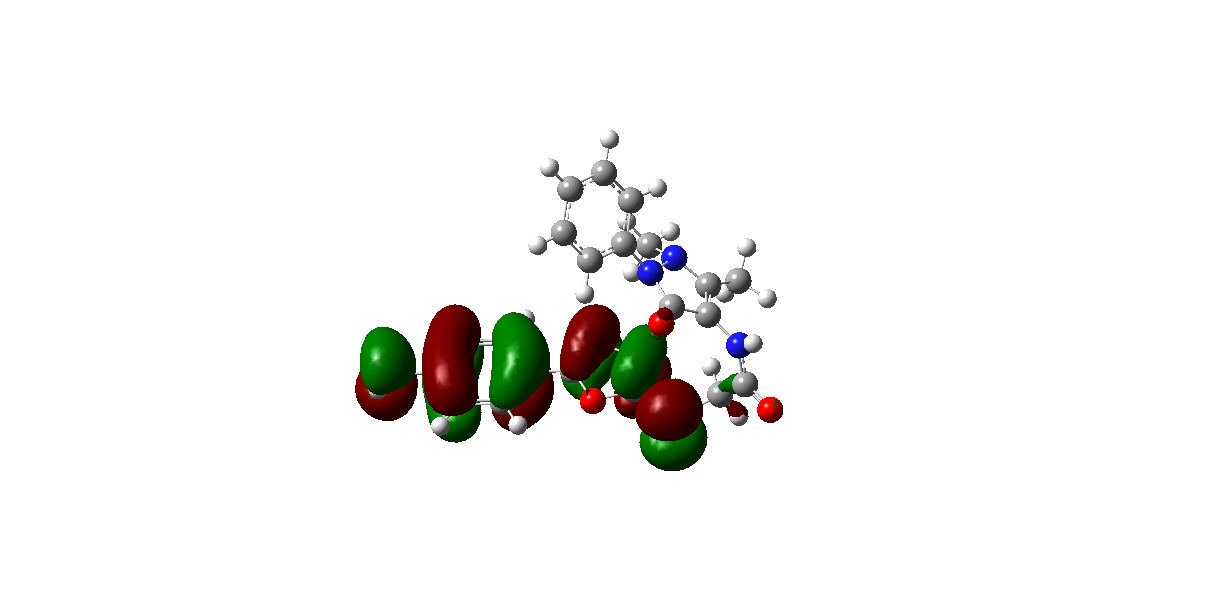 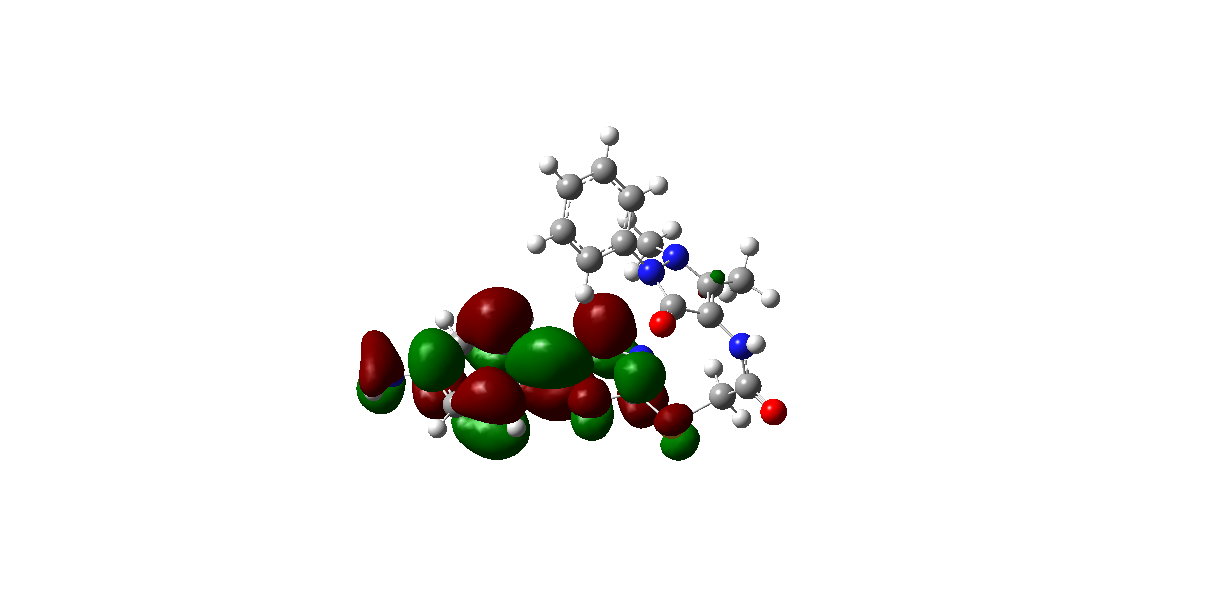  **E_gap_= 4.45 eV**  **HOMO** (-5.98 eV) **LUMO** (-1.52 eV) | |
| **Fig. S36.** Optimized structures, electron density, and HOMO & LUMO for **4h** compound. | |

**Cartesian coordinates**

**TableS1 Cartesian coordinates for the DFT calculation of the compounds 4a-h**

| **Compound 4a** | **Compound 4b** |
| --- | --- |
| ---------------------------------------------------------------------  Center Atomic Atomic Coordinates (Angstroms)  Number Number Type X Y Z  ---------------------------------------------------------------------  1 6 0 -5.859704 -1.498509 0.680788  2 6 0 -5.198703 -2.072908 -0.421112  3 6 0 -4.043404 -1.505506 -0.926512  4 6 0 -3.506506 -0.341505 -0.352812  5 6 0 -4.165307 0.234694 0.742788  6 6 0 -5.321706 -0.332708 1.252488  7 7 0 -7.041103 -2.041111 1.156388  8 6 0 -2.293207 0.233597 -0.900012  9 7 0 -1.571706 -0.184001 -1.899912  10 7 0 -0.497108 0.690001 -2.012912  11 6 0 -0.648310 1.582400 -1.090012  12 8 0 -1.769409 1.366698 -0.337112  13 16 0 0.346488 3.001002 -0.821612  14 6 0 0.884988 2.731203 0.920488  15 6 0 1.943190 1.661105 1.235288  16 7 0 2.489891 1.010506 0.174688  17 8 0 2.225091 1.480206 2.408588  18 6 0 3.468893 -0.012092 0.169888  19 6 0 4.154494 -0.432091 1.314088  20 6 0 5.113396 -1.437589 1.205488  21 6 0 5.416897 -2.048388 -0.013612  22 6 0 4.720696 -1.611690 -1.146412  23 6 0 3.760094 -0.612391 -1.061412  24 6 0 6.436399 -3.157787 -0.104512  25 1 0 -5.602601 -2.969609 -0.879112  26 1 0 -3.540003 -1.957005 -1.772512  27 1 0 -3.767109 1.132295 1.199688  28 1 0 -5.819607 0.127791 2.099188  29 1 0 -7.326903 -1.785112 2.088588  30 1 0 -7.239101 -3.000512 0.918188  31 1 0 1.292286 3.695504 1.232188  32 1 0 0.014889 2.532402 1.545388  33 1 0 2.129291 1.270506 -0.734912  34 1 0 3.936593 0.022509 2.268288  35 1 0 5.638796 -1.749988 2.102488  36 1 0 4.929897 -2.059689 -2.112512  37 1 0 3.227794 -0.297492 -1.953512  38 1 0 7.176799 -3.084285 0.695288  39 1 0 5.959501 -4.140787 -0.020312  40 1 0 6.967199 -3.134586 -1.059612  --------------------------------------------------------------------- | ---------------------------------------------------------------------  Center Atomic Atomic Coordinates (Angstroms)  Number Number Type X Y Z  ---------------------------------------------------------------------  1 6 0 -5.991693 -1.767626 0.736697  2 6 0 -5.226291 -2.394023 -0.264803  3 6 0 -4.108193 -1.773920 -0.791203  4 6 0 -3.713296 -0.504219 -0.339103  5 6 0 -4.475898 0.123179 0.656297  6 6 0 -5.595796 -0.496325 1.186597  7 7 0 -7.137191 -2.368729 1.228797  8 6 0 -2.536098 0.124184 -0.906403  9 7 0 -1.738697 -0.325614 -1.832303  10 7 0 -0.745000 0.629889 -2.005103  11 6 0 -1.014302 1.595889 -1.189003  12 8 0 -2.141602 1.354185 -0.453203  13 16 0 -0.157307 3.118091 -1.038803  14 6 0 0.379993 3.050292 0.723897  15 6 0 1.506196 2.091396 1.143097  16 7 0 2.101698 1.375997 0.154897  17 8 0 1.790696 2.048096 2.329797  18 6 0 3.135301 0.411800 0.258597  19 6 0 3.862302 0.181402 1.435597  20 6 0 4.868604 -0.773595 1.447797  21 6 0 5.174806 -1.514994 0.302297  22 6 0 4.451306 -1.288296 -0.870603  23 6 0 3.438403 -0.331299 -0.883203  24 8 0 6.184809 -2.426991 0.428997  25 6 0 6.533511 -3.211690 -0.702103  26 1 0 -5.519788 -3.373424 -0.628003  27 1 0 -3.523891 -2.265319 -1.559303  28 1 0 -4.188101 1.102779 1.017797  29 1 0 -6.175798 0.005074 1.954097  30 1 0 -7.493392 -2.037230 2.111697  31 1 0 -7.231688 -3.363829 1.097997  32 1 0 0.714791 4.066293 0.944097  33 1 0 -0.480806 2.849990 1.361197  34 1 0 1.733498 1.518696 -0.777203  35 1 0 3.630500 0.743202 2.327497  36 1 0 5.435305 -0.960093 2.352097  37 1 0 4.655707 -1.845495 -1.774603  38 1 0 2.872503 -0.172500 -1.795503  39 1 0 7.345113 -3.860388 -0.376203  40 1 0 5.691413 -3.826892 -1.038303  41 1 0 6.881510 -2.586389 -1.532003  --------------------------------------------------------------------- |

| **Compound 4c** | **Compound 4d** |
| --- | --- |
| ---------------------------------------------------------------------  Center Atomic Atomic Coordinates (Angstroms)  Number Number Type X Y Z  ---------------------------------------------------------------------  1 6 0 -6.440583 -1.950827 -0.301934  2 6 0 -5.468526 -2.403666 0.610216  3 6 0 -4.300858 -1.691002 0.810267  4 6 0 -4.058202 -0.499911 0.106505  5 6 0 -5.024402 -0.046958 -0.803382  6 6 0 -6.195417 -0.758981 -1.005523  7 7 0 -7.586033 -2.689682 -0.536833  8 6 0 -2.826729 0.230011 0.333117  9 7 0 -1.835870 -0.051629 1.120429  10 7 0 -0.894525 0.967663 0.963093  11 6 0 -1.389310 1.793936 0.096624  12 8 0 -2.606504 1.406368 -0.355302  13 16 0 -0.725309 3.279597 -0.542039  14 6 0 0.699824 3.496108 0.609985  15 6 0 1.983139 2.766959 0.195913  16 7 0 1.988524 1.433113 0.504650  17 8 0 2.893934 3.378254 -0.333507  18 6 0 2.993850 0.482042 0.262506  19 6 0 4.243245 0.791381 -0.299393  20 6 0 5.174460 -0.219328 -0.488639  21 6 0 4.904603 -1.548197 -0.134570  22 6 0 3.652494 -1.843036 0.423849  23 6 0 2.709740 -0.845964 0.619739  24 6 0 5.955417 -2.585395 -0.364266  25 6 0 5.660259 -4.024748 0.022752  26 8 0 7.031800 -2.289242 -0.852582  27 1 0 -5.637678 -3.323996 1.159187  28 1 0 -3.561077 -2.048102 1.516125  29 1 0 -4.856779 0.869235 -1.356008  30 1 0 -6.929574 -0.395822 -1.716828  31 1 0 -8.363144 -2.220316 -0.974674  32 1 0 -7.854416 -3.368523 0.158183  33 1 0 0.912820 4.562698 0.571189  34 1 0 0.363457 3.222970 1.610674  35 1 0 1.117732 1.069406 0.894520  36 1 0 4.466288 1.809293 -0.578793  37 1 0 6.141314 0.008921 -0.920454  38 1 0 3.402016 -2.856803 0.711365  39 1 0 1.744860 -1.088880 1.052026  40 1 0 4.794582 -4.408029 -0.525499  41 1 0 6.532340 -4.633741 -0.209563  42 1 0 5.435673 -4.105714 1.090351  --------------------------------------------------------------------- | ---------------------------------------------------------------------  Center Atomic Atomic Coordinates (Angstroms)  Number Number Type X Y Z  ---------------------------------------------------------------------  1 6 0 -6.440999 -1.950553 -0.301919  2 6 0 -5.469244 -2.403188 0.610663  3 6 0 -4.301467 -1.690687 0.810664  4 6 0 -4.058415 -0.499957 0.106423  5 6 0 -5.024309 -0.047195 -0.803874  6 6 0 -6.195436 -0.759053 -1.005958  7 7 0 -7.586530 -2.689299 -0.536771  8 6 0 -2.826838 0.229790 0.332975  9 7 0 -1.835932 -0.051979 1.120178  10 7 0 -0.894536 0.967303 0.962814  11 6 0 -1.389369 1.793618 0.096375  12 8 0 -2.606547 1.406164 -0.355477  13 16 0 -0.725235 3.279270 -0.542210  14 6 0 0.699555 3.495749 0.610233  15 6 0 1.983034 2.766830 0.196341  16 7 0 1.988658 1.432991 0.505065  17 8 0 2.893752 3.378362 -0.332988  18 6 0 2.994060 0.482048 0.262774  19 6 0 4.243182 0.791425 -0.299745  20 6 0 5.174466 -0.219192 -0.489120  21 6 0 4.904946 -1.548018 -0.134619  22 6 0 3.653133 -1.842892 0.424439  23 6 0 2.710328 -0.845901 0.620513  24 6 0 5.955793 -2.585128 -0.364520  25 6 0 5.660872 -4.024480 0.022661  26 8 0 7.032041 -2.288898 -0.853103  27 1 0 -5.638739 -3.323219 1.160037  28 1 0 -3.561937 -2.047612 1.516878  29 1 0 -4.856372 0.868736 -1.356844  30 1 0 -6.929370 -0.396032 -1.717567  31 1 0 -8.363430 -2.219973 -0.975019  32 1 0 -7.855223 -3.367738 0.158514  33 1 0 0.912462 4.562363 0.571679  34 1 0 0.362863 3.222332 1.610738  35 1 0 1.117799 1.069097 0.894643  36 1 0 4.465982 1.809307 -0.579456  37 1 0 6.141103 0.009093 -0.921407  38 1 0 3.402945 -2.856628 0.712324  39 1 0 1.745697 -1.088829 1.053357  40 1 0 4.794915 -4.407795 -0.525129  41 1 0 6.532856 -4.633460 -0.210070  42 1 0 5.436870 -4.105404 1.090392  --------------------------------------------------------------------- |

| **Compound 4e** | **Compound 4f** |
| --- | --- |
| ---------------------------------------------------------------------  Center Atomic Atomic Coordinates (Angstroms)  Number Number Type X Y Z  ---------------------------------------------------------------------  1 6 0 4.440185 -1.794612 0.192474  2 6 0 4.950582 -0.487080 0.301844  3 6 0 4.113038 0.607278 0.196833  4 6 0 2.734976 0.439010 -0.022976  5 6 0 2.223354 -0.863121 -0.137270  6 6 0 3.062525 -1.960073 -0.030459  7 7 0 5.284533 -2.888704 0.252011  8 6 0 1.891322 1.612052 -0.131350  9 7 0 2.232687 2.867271 -0.112587  10 7 0 1.064065 3.614069 -0.211314  11 6 0 0.090007 2.773124 -0.305035  12 8 0 0.530736 1.468437 -0.264859  13 16 0 -1.599529 3.127028 -0.598558  14 6 0 -2.424120 2.297952 0.837908  15 6 0 -2.952264 0.886286 0.601619  16 7 0 -2.029723 -0.044813 0.202067  17 8 0 -4.121457 0.607942 0.800059  18 6 0 -2.288015 -1.384547 -0.048338  19 7 0 -1.332188 -2.183032 -0.431855  20 6 0 -1.819587 -3.455427 -0.621773  21 6 0 -3.146068 -3.625987 -0.379973  22 16 0 -3.868247 -2.120301 0.118298  23 1 0 6.012410 -0.338586 0.467949  24 1 0 4.514194 1.609418 0.284786  25 1 0 1.168226 -1.036212 -0.315261  26 1 0 2.649525 -2.958660 -0.125049  27 1 0 4.869824 -3.787344 0.442952  28 1 0 6.190564 -2.756114 0.673310  29 1 0 -1.742647 2.323761 1.690251  30 1 0 -3.288167 2.918205 1.069821  31 1 0 -1.074860 0.240065 0.012645  32 1 0 -1.141322 -4.232458 -0.946601  33 1 0 -3.738804 -4.523410 -0.461512  --------------------------------------------------------------------- | ---------------------------------------------------------------------  Center Atomic Atomic Coordinates (Angstroms)  Number Number Type X Y Z  ---------------------------------------------------------------------  1 6 0 3.725481 4.210148 -0.247292  2 6 0 2.964186 3.263705 -0.962423  3 6 0 3.042869 1.909672 -0.653620  4 6 0 3.884517 1.457296 0.379195  5 6 0 4.644150 2.401609 1.097450  6 6 0 4.565829 3.753493 0.791267  7 7 0 3.604672 5.573747 -0.516644  8 6 0 3.986670 0.047230 0.717724  9 7 0 4.713648 -0.533422 1.630826  10 7 0 4.470864 -1.908578 1.537372  11 6 0 3.608533 -2.052731 0.581569  12 8 0 3.240185 -0.871292 0.001880  13 16 0 2.889072 -3.567625 0.046789  14 6 0 2.131446 -3.031778 -1.536057  15 6 0 0.849736 -2.192660 -1.492838  16 7 0 0.065531 -2.314913 -0.376918  17 8 0 0.545388 -1.500643 -2.459133  18 6 0 -1.162022 -1.689359 -0.157636  19 16 0 -1.920643 -0.626663 -1.354899  20 6 0 -3.247178 -0.419866 -0.217197  21 6 0 -2.993461 -1.167267 0.951668  22 7 0 -1.800563 -1.878468 0.955170  23 6 0 -4.407702 0.351580 -0.376242  24 6 0 -5.330033 0.362359 0.676082  25 6 0 -5.087773 -0.384005 1.854329  26 6 0 -3.935376 -1.141466 1.996605  27 8 0 -6.504358 1.067132 0.667474  28 6 0 -8.168139 2.522095 -0.228932  29 6 0 -6.829487 1.849347 -0.487698  30 1 0 2.304212 3.595649 -1.761228  31 1 0 2.446528 1.200477 -1.220372  32 1 0 5.295887 2.060233 1.896290  33 1 0 5.155438 4.469934 1.359806  34 1 0 4.391149 6.146176 -0.233435  35 1 0 3.277464 5.807588 -1.446554  36 1 0 2.856885 -2.490279 -2.143525  37 1 0 1.899285 -3.967306 -2.056340  38 1 0 0.395691 -2.875451 0.405180  39 1 0 -4.575168 0.916004 -1.286451  40 1 0 -5.832400 -0.344095 2.644037  41 1 0 -3.749217 -1.713526 2.901174  42 1 0 -8.452675 3.130001 -1.094141  43 1 0 -8.950054 1.776530 -0.057063  44 1 0 -8.111540 3.173131 0.648436  45 1 0 -6.044320 2.598258 -0.660700  46 1 0 -6.884315 1.198743 -1.371492  --------------------------------------------------------------------- |

| **Compound 4g** | **Compound 4h** |
| --- | --- |
| ---------------------------------------------------------------------  Center Atomic Atomic Coordinates (Angstroms)  Number Number Type X Y Z  ---------------------------------------------------------------------  1 6 0 6.175884 -0.142255 0.089274  2 6 0 5.527857 0.566972 1.114277  3 6 0 4.235420 1.036497 0.942097  4 6 0 3.546336 0.814412 -0.258731  5 6 0 4.193971 0.109011 -1.285671  6 6 0 5.483744 -0.360823 -1.116349  7 7 0 7.484989 -0.570539 0.240247  8 6 0 2.190986 1.292030 -0.455599  9 7 0 1.402864 1.135927 -1.473030  10 7 0 0.204490 1.791125 -1.166797  11 6 0 0.360189 2.287034 0.017588  12 8 0 1.586401 2.023927 0.541695  13 16 0 -0.725569 3.216625 1.026071  14 6 0 -2.190431 3.284912 -0.101772  15 6 0 -3.439940 2.827302 0.641848  16 7 0 -3.785194 1.497649 0.566030  17 8 0 -4.116510 3.625697 1.264336  18 6 0 -3.035660 0.428855 0.035447  19 6 0 -2.375007 -0.562104 0.875733  20 7 0 -1.815953 -1.478734 -0.046883  21 7 0 -2.212951 -1.138463 -1.357297  22 6 0 -2.908888 0.053409 -1.269255  23 6 0 -1.531995 -2.835751 0.263209  24 8 0 -2.333498 -0.665367 2.089431  25 6 0 -0.718759 -3.117796 1.365381  26 6 0 -0.445916 -4.441752 1.695487  27 6 0 -0.957313 -5.487015 0.927160  28 6 0 -1.762717 -5.199157 -0.172726  29 6 0 -2.061547 -3.879299 -0.502718  30 6 0 -1.171973 -1.309473 -2.386253  31 6 0 -3.385694 0.755351 -2.494722  32 1 0 6.046442 0.751104 2.049249  33 1 0 3.753788 1.578007 1.746859  34 1 0 3.671017 -0.064947 -2.218017  35 1 0 5.970659 -0.899885 -1.922308  36 1 0 7.804946 -1.301989 -0.375435  37 1 0 7.839199 -0.648793 1.180941  38 1 0 -2.335589 4.325807 -0.381537  39 1 0 -1.943494 2.686659 -0.974106  40 1 0 -4.524018 1.255843 1.217679  41 1 0 -0.325305 -2.305147 1.959468  42 1 0 0.177507 -4.655058 2.556378  43 1 0 -0.733234 -6.515506 1.185393  44 1 0 -2.174804 -6.003591 -0.771669  45 1 0 -2.712755 -3.655343 -1.338222  46 1 0 -0.330537 -0.631800 -2.222234  47 1 0 -0.826691 -2.341772 -2.368744  48 1 0 -1.621653 -1.115366 -3.359232  49 1 0 -4.142885 1.490898 -2.220241  50 1 0 -2.565999 1.277666 -2.998794  51 1 0 -3.827625 0.052232 -3.204402  --------------------------------------------------------------------- | ---------------------------------------------------------------------  Center Atomic Atomic Coordinates (Angstroms)  Number Number Type X Y Z  ---------------------------------------------------------------------  1 6 0 -5.609742 -1.174267 -0.551602  2 6 0 -4.989759 -1.710783 0.592403  3 6 0 -3.791633 -1.196863 1.053404  4 6 0 -3.167073 -0.128569 0.389422  5 6 0 -3.782549 0.407314 -0.750820  6 6 0 -4.982823 -0.105640 -1.215110  7 7 0 -6.782884 -1.721885 -1.041158  8 6 0 -1.911744 0.393226 0.893847  9 7 0 -1.242197 0.028280 1.948702  10 7 0 -0.091451 0.807109 1.984000  11 6 0 -0.150912 1.599244 0.964373  12 8 0 -1.280028 1.403683 0.218682  13 16 0 0.979649 2.874973 0.554042  14 6 0 1.487064 2.373353 -1.146946  15 6 0 2.458090 1.194216 -1.346948  16 7 0 2.826590 0.519426 -0.209988  17 8 0 2.823262 0.954584 -2.475830  18 6 0 3.648951 -0.624079 -0.109173  19 7 0 3.651153 -1.492206 -1.113931  20 6 0 4.420385 -2.577921 -0.992144  21 6 0 5.192543 -2.858628 0.130318  22 6 0 5.165976 -1.947329 1.185488  23 6 0 4.390156 -0.803989 1.068422  24 1 0 -5.455468 -2.540460 1.113844  25 1 0 -3.322400 -1.616408 1.934727  26 1 0 -3.318210 1.232682 -1.276396  27 1 0 -5.442222 0.317924 -2.101949  28 1 0 -7.330550 -1.159276 -1.673288  29 1 0 -7.333718 -2.281321 -0.408954  30 1 0 0.597473 2.174116 -1.744495  31 1 0 1.963523 3.261533 -1.565445  32 1 0 2.534160 0.933192 0.666062  33 1 0 4.404292 -3.261698 -1.836096  34 1 0 5.789579 -3.760945 0.176871  35 1 0 5.747843 -2.120338 2.083663  36 1 0 4.355386 -0.065477 1.860989  --------------------------------------------------------------------- |
